# Functional insights from the GC-poor genomes of two aphid parasitoids, *Aphidius ervi* and *Lysiphlebus fabarum*

**DOI:** 10.1186/s12864-020-6764-0

**Published:** 2020-05-29

**Authors:** Alice B. Dennis, Gabriel I. Ballesteros, Stéphanie Robin, Lukas Schrader, Jens Bast, Jan Berghöfer, Leo W. Beukeboom, Maya Belghazi, Anthony Bretaudeau, Jan Buellesbach, Elizabeth Cash, Dominique Colinet, Zoé Dumas, Mohammed Errbii, Patrizia Falabella, Jean-Luc Gatti, Elzemiek Geuverink, Joshua D. Gibson, Corinne Hertaeg, Stefanie Hartmann, Emmanuelle Jacquin-Joly, Mark Lammers, Blas I. Lavandero, Ina Lindenbaum, Lauriane Massardier-Galata, Camille Meslin, Nicolas Montagné, Nina Pak, Marylène Poirié, Rosanna Salvia, Chris R. Smith, Denis Tagu, Sophie Tares, Heiko Vogel, Tanja Schwander, Jean-Christophe Simon, Christian C. Figueroa, Christoph Vorburger, Fabrice Legeai, Jürgen Gadau

**Affiliations:** 1 Department of Aquatic Ecology, Eawag, 8600 Dübendorf, Switzerland; 2 Institute of Integrative Biology, ETH Zürich, 8092 Zürich, Switzerland; 3 Institute of Biochemistry and Biology, University of Potsdam, 14476 Potsdam, Germany; 4 Instituto de Ciencias Biológicas, Universidad de Talca, Talca, Chile; 5 Centre for Molecular and Functional Ecology in Agroecosystems, Universidad de Talca, Talca, Chile; 6 Laboratorio de Control Biológico, Instituto de Ciencias Biológicas, Universidad de Talca, Talca, Chile; 7 IGEPP, Agrocampus Ouest, INRAE, Université de Rennes, 35650 Le Rheu, France; 8 Université de Rennes 1, INRIA, CNRS, IRISA, 35000 Rennes, France; 9 Institute for Evolution and Biodiversity, Universität Münster, Münster, Germany; 10 Department of Ecology and Evolution, Université de Lausanne, 1015 Lausanne, Switzerland; 11 Institute of Zoology, Universität zu Köln, 50674 Köln, Germany; 12 Groningen Institute for Evolutionary Life Sciences, University of Groningen, Groningen, The Netherlands; 13 Aix-Marseille Univ, CNRS, INP, Inst Neurophysiopathol, PINT, PFNT, Marseille, France; 14 Department of Environmental Science, Policy, & Management, University of California, Berkeley, Berkeley, CA 94720 USA; 15 Université Côte d’Azur, INRAE, CNRS, ISA, Sophia Antipolis, France; 16 Department of Sciences, University of Basilicata, 85100 Potenza, Italy; 17 Department of Biology, Georgia Southern University, Statesboro, GA 30460 USA; 18 Department of Environmental Systems Sciences, D-USYS, ETH Zürich, Zürich, Switzerland; 19 INRAE, Sorbonne Université, CNRS, IRD, UPEC, Université Paris Diderot, Institute of Ecology and Environmental Sciences of Paris, iEES-Paris, F-78000 Versailles, France; 20 Department of Biology, Earlham College, Richmond, IN 47374 USA; 21 Department of Entomology, Max Planck Institute for Chemical Ecology, Jena, Germany

**Keywords:** Parasitoid wasp, Aphid host, *Aphidius ervi*, *Lysiphlebus fabarum*, GC content, de novo genome assembly, DNA methylation loss, Chemosensory genes, Venom proteins, Toll and Imd pathways

## Abstract

**Background:**

Parasitoid wasps have fascinating life cycles and play an important role in trophic networks, yet little is known about their genome content and function. Parasitoids that infect aphids are an important group with the potential for biological control. Their success depends on adapting to develop inside aphids and overcoming both host aphid defenses and their protective endosymbionts.

**Results:**

We present the de novo genome assemblies, detailed annotation, and comparative analysis of two closely related parasitoid wasps that target pest aphids: *Aphidius ervi* and *Lysiphlebus fabarum* (Hymenoptera: Braconidae: Aphidiinae)*.* The genomes are small (139 and 141 Mbp) and the most AT-rich reported thus far for any arthropod (GC content: 25.8 and 23.8%). This nucleotide bias is accompanied by skewed codon usage and is stronger in genes with adult-biased expression. AT-richness may be the consequence of reduced genome size, a near absence of DNA methylation, and energy efficiency. We identify missing desaturase genes, whose absence may underlie mimicry in the cuticular hydrocarbon profile of *L. fabarum*. We highlight key gene groups including those underlying venom composition, chemosensory perception, and sex determination, as well as potential losses in immune pathway genes.

**Conclusions:**

These findings are of fundamental interest for insect evolution and biological control applications. They provide a strong foundation for further functional studies into coevolution between parasitoids and their hosts. Both genomes are available at https://bipaa.genouest.org.

## Background

Parasites are ubiquitously present across all of life [1, 2]. Their negative impact on host fitness can impose strong selection on hosts to resist, tolerate, or escape potential parasites. Parasitoids are a special group of parasites whose successful reproduction is fatal to the host [3, 4]. The overwhelming majority of parasitoid insects are hymenopterans that parasitize other terrestrial arthropods, and they are estimated to comprise up to 75% of the species-rich insect order Hymenoptera [4–7]. Parasitoid wasps target virtually all insects and developmental stages (eggs, larvae, pupae, and adults), including other parasitoids [4, 8–10]. Parasitoid radiations appear to have coincided with those of their hosts [11], and there is ample evidence that host-parasitoid relationships impose strong reciprocal selection, promoting a dynamic process of antagonistic coevolution [12–14].

Parasitoids of aphids play an economically important role in biological pest control [15, 16], and aphid-parasitoid interactions are an excellent model to study antagonistic coevolution, specialization, and speciation [17, 18]. While parasitoids that target aphids have evolved convergently several times, their largest radiation is found in the braconid subfamily Aphidiinae, which contains at least 400 described species across 50 genera [9, 19]. As koinobiont parasitoids, their development progresses initially in still living, feeding, and developing hosts, and ends with the aphids’ death and the emergence of adult parasitoids. Parasitoids increase their success with a variety of strategies, including host choice [20, 21], altering larval development timing [22], injecting venom during stinging and oviposition, and developing special cells called teratocytes to circumvent host immune responses [23–27]. In response to strong selection imposed by parasitoids, aphids have evolved numerous defenses, including behavioral strategies [28], immune defenses [29], and symbioses with heritable endosymbiotic bacteria whose integrated phages can produce toxins to hinder parasitoid success [12, 30, 31].

**Fig. 1:**
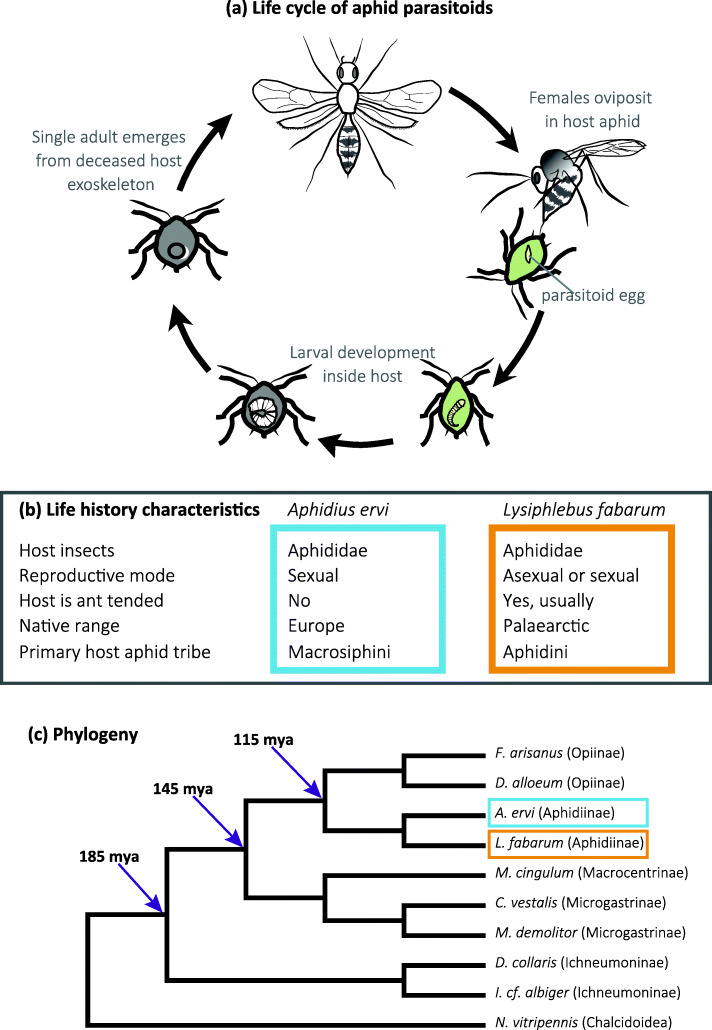
Life history characteristics of two aphid parasitoids*.* **a** Generalized life cycle of *Aphidius ervi *and *Lysiphlebus fabarum*, two parasitoid wasp species that infect aphid hosts. Figure by Alice Dennis. **b** Life history characteristics of the two species. **c** Phylogenetic relationships of the Ichneumonoidea species listed in Table 2, rooted with *Nasonia vitripennis* (Chalcidoidea). Average divergence times between major groups and phylogenetic relationships have been modified, after Supplemental Figure S1 in [9, 11], *Ichneumon cf. albiger* is also included to better match dating available from [11]. The subfamily for each species is given after the species name

The parasitoid wasps *Lysiphlebus fabarum* and *Aphidius ervi* (Braconidae: Aphidiinae) are closely related endoparasitoids of aphids (Fig. 1) [9, 11, 38]. In the wild, both species are found infecting a wide range of aphid species although their host ranges differ, with *A. ervi* more specialized on aphids in the Macrosiphini tribe and *L. fabarum* on the Aphidini tribe [39, 40]. Experimental evolution studies in both species have shown that wild-caught populations can counter-adapt to cope with aphids and the defenses of their endosymbionts, and that the coevolutionary relationships between parasitoids and the aphids’ symbionts likely fuel diversification of both parasitoids and their hosts [41–43]. While a number of parasitoid taxa are known to inject viruses and virus-like particles into their hosts, there is thus far no evidence that this occurs in parasitoids that target aphids; recent studies have identified two abundant RNA viruses in *L. fabarum* [44, 45], but whether this impacts their ability to parasitize is not yet clear.

*Aphidius ervi* and *L. fabarum* differ in several important life history traits, and are expected to have experienced different selective regimes as a result. *Aphidius ervi* has been successfully introduced as a biological control agent in Nearctic and Neotropic regions. Studies on both native and introduced populations of *A. ervi* have shown ongoing evolution with regard to host preferences, gene flow, and other life history components [46–49]. *Aphidius ervi* is known to reproduce only sexually, whereas *L. fabarum* is capable of both sexual and asexual reproduction. In fact, wild *L. fabarum* populations are more commonly composed of asexually reproducing (thelytokous) individuals [50], and this asexuality is not due to infection with endosymbionts like *Wolbachia* [51]. In asexual populations of *L. fabarum*, diploid females produce diploid female offspring via central fusion automixis [52]. While they are genetically differentiated, sexual and asexual populations appear to maintain gene flow; both reproductive modes and genome-wide heterozygosity are maintained in the species as a whole [50, 53, 54]. *Aphidius ervi* and *L. fabarum* have also experienced different selective regimes with regard to their cuticular hydrocarbon profiles and chemosensory perception. *Lysiphlebus* target aphid species that are ant-tended, and ants are known to prevent parasitoid attacks on “their“ aphids [55]. To counter ant defenses, *L. fabarum* has evolved the ability to mimic the cuticular hydrocarbon profile of the aphid hosts [56, 57]. This enables the parasitoids to circumvent ant defenses and access this challenging ecological niche, from which they also benefit nutritionally; they are the only parasitoid species thus far documented to behaviorally encourage aphid honeydew production and consume this high-sugar reward [55, 58, 59].

We present here the genomes of *A. ervi* and *L. fabarum*, assembled de novo using a hybrid sequencing approach. The two genomes are strongly biased towards AT nucleotides. We have examined GC content in the context of host environment, nutrient limitation, and gene expression. By comparing these two genomes, we identify key functional specificities in genes underlying venom composition, oxidative phosphorylation (OXPHOS), cuticular hydrocarbon (CHC) composition, sex determination, development (Osiris), and chemosensory perception. In both species, we identify putative losses in key immune genes and an apparent lack of key DNA methylation machinery. These are functionally important traits associated with success infecting aphids and the evolution of related traits across all of Hymenoptera.

## Results

### Two de novo genome assemblies

The genome assemblies for *A. ervi* and *L. fabarum* were constructed using hybrid approaches that incorporated high-coverage short read (Illumina) and long-read (Pac Bio) sequences, and were assembled with different strategies (Supplementary Tables 1 and 2). This produced two high quality genome assemblies (N50 in *A. ervi*: 581 kb, in *L. fabarum*: 216 kb) with similar total lengths (*A. ervi*: 139Mbp, *L. fabarum*: 141Mbp) but different ranges of scaffold-sizes (Table 1, Supplementary Table 3). The length of these assemblies is in range of that predicted by a kmer analysis with the K-mer Analysis Toolkit (KAT) (Supplementary Figure 1) [60], which predicted *A. ervi* at 142.83Mbp and *L. fabarum* at 99.26Mbp. However, the *L. fabarum* assembly is larger than the estimate from KAT; we suspect that this may be due to duplications in the assembly, and future work should address these duplications. These assembly lengths are also within previous estimates of 110-180Mbp for braconids, including *A. ervi* [61, 62] and are on par with those predicted in other hymenopteran genomes (Table 2). Both genomes were screened for potential contamination (Supplementary Figures 2 and 3, Supplementary Table 6, Additional files 1 and 2) based on BLAST [63] matches to host aphids and results of the program blobtools [64], which jointly examines GC content and sequencing depth. In addition to identifying likely bacterial scaffolds (*A. ervi*: 35 scaffolds/ 106Kbp removed, no scaffolds removed from *L. fabarum*), blobtools revealed one outlier scaffold in *L. fabarum* with high coverage and low GC content (tig00001511, 10,205 bp, 11.1% GC). A BLASTn search against the NCBI *nt* database matched this to the mitochondrial genome of *Aphidius gifuensis*. In this and other parasitoids, the mitochondrial genome has been shown to be highly enriched with AT repeats, with GC contents that are nearly as low as the 11.1% found in this *L. fabarum* scaffold (13.5–17.5%) [65]. The assemblies are available in NCBI (PRJNA587428, SAMN13190903–4) and can be accessed via the BioInformatics Platform for Agroecosystem Arthropods (BIPAA, https://bipaa.genouest.org), which contains the full annotation reports, predicted genes, and can be searched via both keywords and BLAST.

**Table 1:** Assembly and draft annotation statistics

	*A. ervi*	*L. fabarum*
Assembly statistics		
Total length (bp)	138,845,131	140,705,580
Longest scaffold (bp)	3,671,467	2,183,677
scaffolds	5743	1698
scaffolds ≥3000 bp	1503	1698
N50 (bp)	581,355	216,143
GC %	25.8%	23.8%
Annotation statistics		
Exons	95,299	74,701
Introns	74,971	59,498
CDS	20,328	15,203
% genome covered by CDS	17.8%	14.9%
GC % in CDS	31.9%	29.8%
GC % of 3rd position in CDS	15.5%	10.7%
CDS with transcriptomic support	77.8%	88.3%

**Table 2:** Assembly summary statistics compared to other parasitoid genomes. All species are from the family Braconidae, except for *N. vitripennis* (Pteromalidae) and *D. collaris* (Ichneumonidae). Protein counts from the NCBI genome deposition

Parasitoid species	Assembly	Total Length (Mbp)	Scaffold Count (N50, Kbp)	Contig count (N50, Kbp)	Predicted genes (CDS)	GC (%)	NCBI BioProject
*Aphidius ervi*	A. ervi_v3	138.8	5743 (581.4)	12,948 (25.2)	20,344	25.8	*This paper*
*Lysiphlebus fabarum*	L. fabarum_v1	140.7	na	1698 (216.1)	15,203	23.8	*This paper*
*Cotesia vestalis*	ASM95615v1	178.55	1437 (2609.6)	6820 (51.3)	11,278	29.96	PRJNA307296 [32]
*Diachasma alloeum*	Dall2.0	384.4	3313 (657.0)	24,824 (45.5)	na	38.3	PRJNA284396 [33]
*Fopius arisanus*	ASM80636v1	153.6	1042 (980.0)	8510 (51.9)	18,906	39.4	PRJNA258104 [34]
*Macrocentrus cingulum*	MCINOGS1.0	132.36	5696 (192.4)	13,289 (64.9)	11,993	35.66	PRJNA361069 [35]
*Microplitis demolitor*	Mdem 2	241.2	1794 (1140)	27,508 (14.12)	18,586	33.1	PRJNA251518 [36]
*Diadromus collaris*	ASM939471v1	399.17	2731 (1030.3)	20,676 (25,941)	15,328	37.37	PRJNA307299 [32]
*Nasonia vitripennis*	Nvit_2.1	295.7	6169 (709)	26,605 (18.5)	24,891	40.6	PRJNA13660 [37]

We constructed linkage groups for *L. fabarum* using phased SNPs from the haploid sons of a single female wasp from a sexually reproducing population. This placed the 297 largest scaffolds (> 50% of the nucleotides, Supplementary Table 7, Supplementary Figure 4, Additional file 3) onto the expected six chromosomes [52]. With this largely contiguous assembly, we identified stretches of syntenic sequence between the two genomes, with > 60 k links in alignments made by NUCmer [66] and > 350 large syntenic blocks that match the six *L. fabarum* chromosomes to 28 *A. ervi* scaffolds (Supplementary Figures 5 and 6).

The Maker2 annotation pipeline predicted coding genes (CDS) in both genomes separately, and these were functionally annotated against the NCBI *nr* database [67], gene ontology (GO) terms [68, 69], and predictions for known protein motifs, signal peptides, and transmembrane domains (Supplementary Table 5). In *A. ervi* there were 20,328 predicted genes comprising 24.7Mbp, whereas in *L. fabarum* there were 15,203 genes across 21.9Mbp (Table 1). Matches to the BUSCO (Benchmarking Universal Single-Copy Orthologs) genes assessed completeness against the Insecta database genes at both the nucleotide level (*A. ervi*: 94.8%, *L. fabarum*: 76.3%, Supplementary Table 4) and protein level in the predicted genes (*A. ervi*: 93.7%, *L. fabarum*: 95.9%). These protein level matches are close to those found in other assembled parasitoid genomes, which report between 96 and 99% total coverage of BUSCO genes [32–37]. In both species, there was also high transcriptomic support for the predicted genes (77.8% in *A. ervi* and 88.3% in *L. fabarum*).

A survey of transposable Elements (TEs) identified a similar overall number of putative TE elements in the two assemblies (*A. ervi*: 67,695 and *L. fabarum*: 60,306, Supplementary Table 8). Despite this similarity, the overall coverage by repeats is larger in the assembly of *L. fabarum* (41%, 58Mbp) than in *A.* *ervi* (22%, 31Mbp) and both assemblies differ in the TE classes that they contain (Supplementary Table 8, Supplementary Figures 7 and 8). This could be the product of their different assembly methods. However, direct estimates from unassembled short read data suggest even higher repeat content in *L. fabarum* (49.1% vs. 29.3% in *A. ervi*), largely explained by differences in simple repeats and low-complexity sequences (Supplementary Table 9).

To examine genes that may underlie novel functional adaptation, we identified sequences that are unique within the predicted genes in the *A. ervi* and *L. fabarum* genomes. We defined these orphan genes as predicted genes with transcriptomic support and with no identifiable homology based on searches against the NCBI *nr*, *nt*, and Swissprot databases. We identified 2568 (*A. ervi*, Additional file 4) and 968 (*L. fabarum*, Additional file 5) putative orphans.

### GC content

**Fig. 2:**
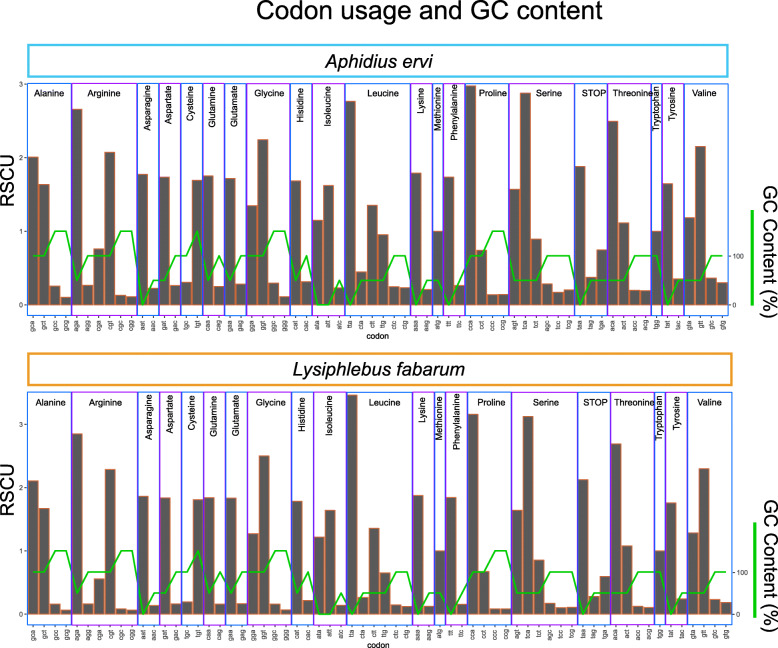
Codon usage and GC content in predicted genes. Proportions of all possible codons, as used in the predicted genes in *A. ervi* (top) and *L. fabarum* (bottom). Codon usage was measured as relative synonymous codon usage (RSCU), which scales usage to the number of possible codons for each amino acid. Codons are listed at the bottom and are grouped by the amino acid that they encode. The green line depicts GC content (%) of the codon

The *L. fabarum* and *A. ervi* genomes are the most GC-poor of insect genomes sequenced to date (GC content: 25.8 and 23.8% for *A. ervi* and *L. fabarum*, respectively, Table 1, Supplementary Figure 9, Additional file 6). This nucleotide bias is accompanied by strong codon bias in the predicted genes, meaning that within the possible codons for each amino acid, the two genomes are almost universally skewed towards the codon(s) with the lowest GC content (measured as Relative Synonymous Codon Usage, RSCU, Fig. 2). We examined potential constraints in codon usage between our two species’ genomes and taxa associated with this parasitoid-host-endosymbiont system (Supplementary Table 10). We found no evidence of similarity in codon usage (scaled as RSCU) nor nitrogen content (scaled per amino acid) between parasitoids and host aphids, the primary endosymbiont *Buchnera,* or the secondary endosymbiont *Hamiltonella* (Supplementary Figures 10, 11 and 12).

As selective pressure for translational efficiency, stability, and secondary structure should be higher in more highly expressed genes [70–73], we examined GC content in relation to expression level. We first explored constraints by looking at overall expression levels. In both species, the most highly expressed 10% of genes had significantly higher GC and higher nitrogen contents, although the higher number of nitrogen molecules in Guanine and Cytosine means that these two measures cannot be entirely disentangled (Additional file 7, Supplementary Figure 13). This is in line with observations across many taxa, and with the idea that GC-rich mRNA has increased expression via its stability and secondary structure [72, 73].

**Fig. 3:**
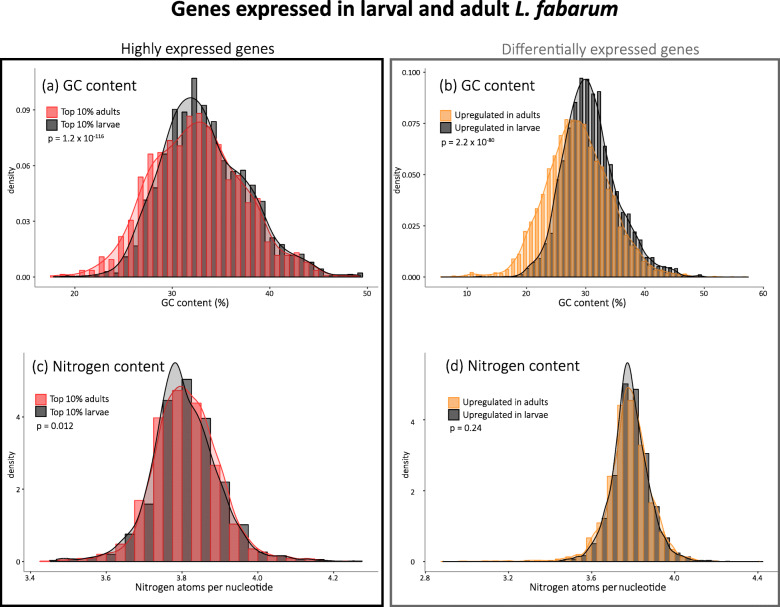
GC and nitrogen content of expressed genes. We observe significant differences in the GC content of genes biased towards adult or larval *L. fabarum* in: (**a**) the 10% most highly expressed genes and (**b**) genes that are significantly differentially expressed between adults and larvae. In contrast, there is no difference in the nitrogen content of the same set of genes (**c**, **d**). *P*-values are from a two-sided t-test

We next utilized available transcriptomic data from adult and larval *L. fabarum* to examine life-stage specific constraints. We found higher GC content in larvae-biased genes in *L. fabarum* (Fig. 3). This was true when we compared both the 10% most highly expressed genes in adults (32.6% GC) and larvae (33.2%, *p* = 1.2e-116, Fig. 3, Additional file 7), and this pattern holds even more strongly for genes that are differentially expressed between adults (upregulated in adults: 28.7% GC) and larvae (upregulated in larvae: 30.7% GC, *p* = 2.2e-80). Note that the most highly expressed genes overlap partially with those that are differentially expressed (Additional file 7). At the same time, nitrogen content did not differ in either comparison (Fig. 3).

### Gene family expansions

To examine gene families that may have undergone expansions in association with functional divergence and specialization, we identified groups of orthologous genes that have increased and decreased in size in the two genomes, relative to one another. We identified these species-specific gene-family expansions using the Orthologous MAtrix (OMA) standalone package [74]. OMA predicted 8817 OMA groups (strict 1:1 orthologs) and 8578 Hierarchical Ortholog Groups (HOGs, Additional file 8). Putative gene-family expansions would be found in the predicted HOGs, because they are calculated to allow for > 1 member per species. Among these, there were more groups in which *A. ervi* possessed more genes than *L. fabarum* (865 groups with more genes in *A. ervi*, 223 with more in *L. fabarum*, Supplementary Figure 14, Additional file 8). To examine only the largest gene-family expansions, we looked further at the HOGs containing > 20 genes (10 HOG groups, Supplementary Figure 15). Strikingly, the four largest expansions were more abundant in *A. ervi* and were all identified as F-box proteins/Leucine-rich-repeat proteins (*LRR*, total: 232 genes in *A. ervi* and 68 in *L. fabarum*, Supplementary Figure 15, Additional file 8). This signature of expansion does not appear to be due to fragmentation in the *A. ervi* assembly; the size of scaffolds containing *LRR*s is on average larger in *A. ervi* than in *L. fabarum* (Welch two-sample t-test, *p* = 0.001, Supplementary Figure 16). The six largest gene families that were expanded in *L. fabarum*, relative to *A. ervi,* were less consistently annotated. Interestingly, they contained two different histone proteins: Histone H2B and H2A (Supplementary Figure 15).

### Venom proteins

**Fig. 4:**
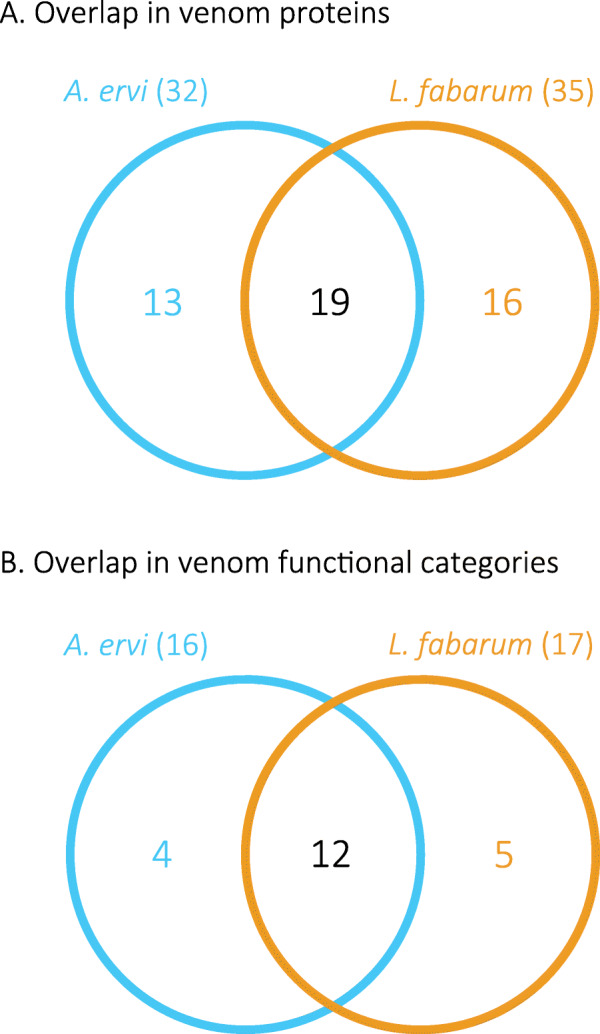
Overlap in venom proteins and functional categories between *A. ervi* and *L. fabarum*. Venn diagrams show the number of (**a**) venom proteins and (**b**) venom functional categories that are shared or unique to *A. ervi* and *L. fabarum*

We examined the venom of both species using evidence from proteomics, transcriptomics, and manual gene annotation. The venom gland of *L. fabarum* is morphologically different from that of *A. ervi* (Supplementary Figure 17). A total of 35 *L. fabarum* proteins were identified as putative venom proteins by 1D gel electrophoresis and mass spectrometry, combined with transcriptomic and the genomic data (Supplementary Figure 18, Additional file 9) [42]. These putative venom proteins were identified based on predicted secretion (for complete sequences) and the absence of a match to typical cellular proteins (e.g. actin, myosin). To match the analysis between the two taxa, previously generated *A. ervi* venom protein data [24] were analyzed using the same criteria as for *L. fabarum*. This identified 32 putative venom proteins in *A. ervi* (Additional file 9). More than 50% of the proteins are shared between species (Fig. 4a and Additional file 9), corresponding to more than 70% of the predicted putative functional categories (Fig. 4b and Additional file 9). Among the venom proteins shared between both parasitoids, a gamma glutamyl transpeptidase (GGT1) was the most abundant protein in the venom of both *A. ervi* [24] and *L. fabarum* (Additional file 9). As previously reported for *A. ervi* [24], a second GGT venom protein (GGT2) containing mutations in the active site was also found in the venom of *L. fabarum* (Supplementary Figures 19 and 20).

**Fig. 5:**
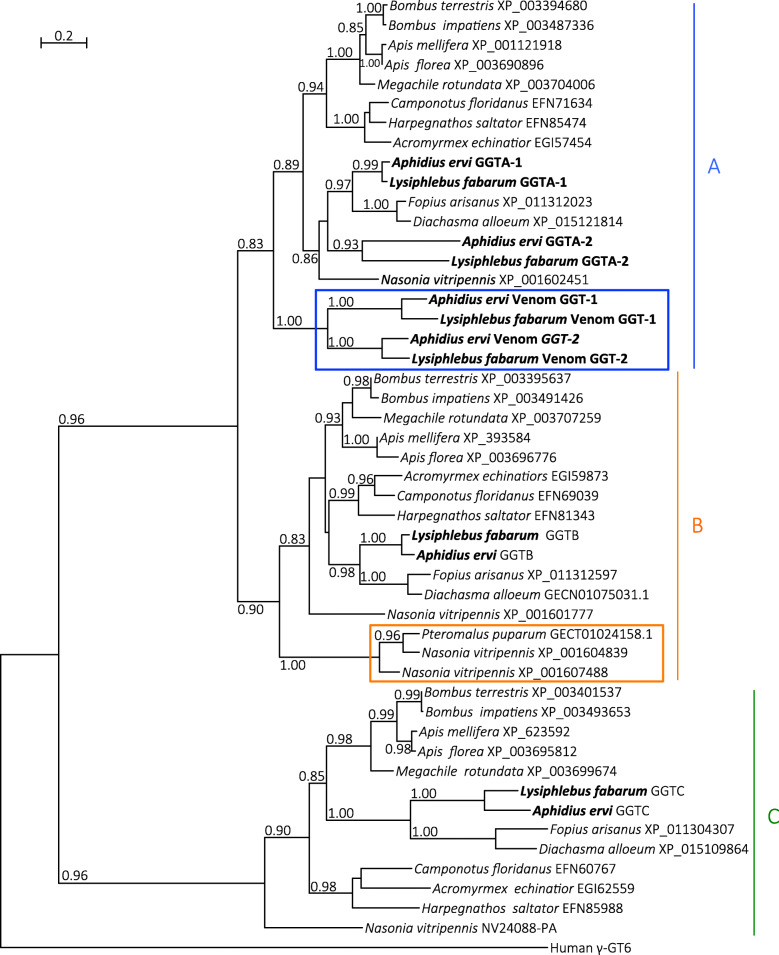
Phylogeny of hymenopteran GGT sequences. Phylogeny depicting gamma glutamyl transpeptidase (GGT) sequences across Hymenoptera. Numbers correspond to accessions (NCBI protein, NCBI TSA, and NasoniaBase for NV24088-PA). *A. ervi/L. fabarum* and *Nasonia vitripennis/ Pteromalus puparum* venom GGT sequences are marked with blue and orange rectangles respectively. Letters A, B and C indicate the major clades observed for hymenopteran GGT sequences. Numbers at corresponding nodes are aLRT values. Only aLRT support values greater than 0.8 are shown. The outgroup is human GGT6 sequence

Phylogenetic analysis (Fig. 5) showed that the *A. ervi* and *L. fabarum* GGT venom proteins occur in a single clade in which the GGT1 venom proteins group separately from GGT2 venom proteins, thus suggesting that they originated from a duplication that occurred prior to the split from their most recent common ancestor. As previously shown for *A. ervi*, the GGT venom proteins of *A. ervi* and *L. fabarum* are found in one of the three clades described for GGT proteins of non-venomous hymenopterans (clade “A”, Fig. 5) [24]. Within this clade, venomous and non-venomous GGT proteins had a similar exon structure, except for exon 1 that corresponds to the signal peptide only being present in venomous GGT proteins (Supplementary Figure 19). Several *LRR* proteins were found in the venom of *L. fabarum* as well, although these results should be interpreted with caution since the sequences were incomplete and the presence of a signal peptide could not be confirmed (Additional file 9). Moreover, these putative venom proteins were only identified from transcriptomic data of the venom apparatus and we could not find any corresponding annotated gene in the genome. This supports the idea that gene-family expansions in putative F-box/*LRR* proteins identified in the analysis with OMA are not related to venom production.

Approximately 50% of the identified venom proteins were unique to either *A. ervi* or *L. fabarum* (Additional file 9). However, many of these proteins had no predicted function, making it difficult to hypothesize their possible role in parasitism success. Among those that could be identified was apolipophorin in the venom of *L. fabarum*, but not in *A. ervi.* Apolipophorin is an insect-specific apolipoprotein involved in lipid transport and innate immunity, and is not commonly found in venoms. Among parasitoid wasps, apolipophorin has been described in the venom of the ichneumonid *Hyposoter didymator* [75] and the encyrtid *Diversinervus elegans* [76], but its function is yet to be deciphered. Apolipophorin is also present in low abundance in honeybee venom where it could have antibacterial activity [77, 78]. In contrast, we could not find *L. fabarum* homologs for any of the three secreted cysteine-rich toxin-like peptides that are highly expressed in the *A. ervi* venom apparatus (Additional file 9).

### Key gene families

We manually annotated 719 genes in *A. ervi* and 642 in *L. fabarum* (Table 3) using Apollo, hosted on the BIPAA website: bipaa.genouest.org [79–81].

**Table 3:** Summary of manual curations of select gene families in the two parasitoid genomes

Category	*A. ervi*	*L. fabarum*
Venom proteins	32	35
Desaturases	14	11
Immune genes	270	264
Osiris genes	21	25
Mitochondrial Oxidative Phosphorylation System (OXPHOS)^a^	75	74
Chemosensory group		
Chemosensory: Odorant receptors (ORs)	228	156
Chemosensory: Ionotropic chemosensory receptors (IRs)	42	40
Chemosensory: Odorant-binding proteins (OBPs)	14	14
Chemosensory: Chemosensory proteins (CSPs)	11	13
Sex determination group		
Sex determination: Core (transformer, doublesex)	4	3
Sex determination: Related genes	6	5
DNA methylation genes	2	2
TOTALS	**719**	**642**

^a^Note: includes possible assembly duplicates

### Desaturases

Annotation of desaturase genes found that *L. fabarum* has three fewer desaturase genes than *A. ervi* (Table 3, Supplementary Table 12, Supplementary Figure 24). Examination of the cuticular hydrocarbon (CHC) profiles of *L. fabarum* and *A. ervi* identified several key differences. The CHC profile of *L. fabarum* is dominated by saturated hydrocarbons (alkanes), contains only trace alkenes, and is completely lacking dienes (Supplementary Figures 21 and 23). In contrast, *A. ervi* females produce a large amount of unsaturated hydrocarbons, with a substantial amount of alkenes and alkadienes in their CHC profiles (app. 70% of the CHC profile are alkenes/alkadienes, Supplementary Figures 22 and 23).

### Immune genes

We searched for immune genes in the two genomes based on a list of 373 immunity related genes, collected primarily from the *Drosophila* literature (Additional file 10). We found and annotated > 70% of these in both species (*A. ervi*: 270, *L. fabarum*: 264 genes). We compared these with the immune genes used to define the main *Drosophila* immune pathways (Toll, Imd, and JAK-STAT, Supplementary Table 13) and conserved in a number of insect species [82–84]. In the genome of both wasps, some of the genes encoding proteins of the Imd and Toll pathways were absent (Supplementary Table 13, Supplementary Figure 25, Additional file 10). Only one GNBP (Gram Negative Binding Protein) involved in Gram positive bacteria and fungi recognition was found in *A. ervi* and *L. fabarum*, compared to the three known from *Drosophila* and 2 from *Apis* (Supplementary Table 13). PGRPs (Peptidoglycan Recognition Proteins) are involved in the response to Gram-positive bacteria [85], and we did not find any significant matches to these, although two short matches did not meet our selection criteria (blast matches >1e-5). Similarly, the only match to *imd* itself was very poor in *A. ervi* (e-value: 0.058, Additional file 10), and we could not find any match in *L. fabarum*. The components of the Toll and JAK/Stat pathways appear to be less affected than those of the Imd pathway, although in all cases the output effectors remained mainly unknown.

### Osiris genes

The Osiris genes are an insect-specific gene family that underwent multiple tandem duplications early in insect evolution. These genes are essential for proper embryogenesis [86] and pupation [87, 88], and are also tied to immune and toxin-related responses (e.g.) [87, 89] and developmental polyphenism [90, 91].

We found 21 and 25 putative Osiris genes in the *A. ervi* and *L. fabarum* genomes, respectively (Supplementary Tables 14 and 15, Supplementary Figure 26). In insects with well assembled genomes, there is a consistent synteny of approximately 20 Osiris genes; this cluster usually occurs in a ~ 150kbp stretch and gene synteny is conserved in all known Hymenoptera genomes. The Osiris cluster is also largely devoid of non-Osiris genes in most of the Hymenoptera, but the assemblies of *A. ervi* and *L. fabarum* suggest that if the cluster is actually syntenic in these species, there are interspersed non-Osiris genes (black boxes in Supplementary Figures 27 and 28).

In support of their role in defense (especially metabolism of xenobiotics and immunity), these genes were much more highly expressed in larvae than in adults (Supplementary Table 15). We hypothesize that their upregulation in larvae is an adaptive response to living within a host. Because of the available transcriptomic data, we could only make this comparison in *L. fabarum*. Here, 19 of the 26 annotated Osiris genes were significantly upregulated in larvae over adults (Supplementary Table 15, Additional file 11). In both species, transcription in adults was very low, with fewer than 10 raw reads per cDNA library sequenced, and most often less than one read per library (Supplementary Tables 14 and 15).

### OXPHOS

In most eukaryotes, mitochondria provide the majority of cellular energy (in the form of adenosine triphosphate, ATP) through the oxidative phosphorylation (OXPHOS) pathway. OXPHOS genes are an essential component of energy production, and their amino acid substitution rate in Hymenoptera is higher relative to any other insect order [92]. We identified 69 out of 71 core OXPHOS genes in both genomes, as well as five putative duplication events that are apparently not assembly errors (Supplementary Table 16, Additional file 12). The gene sets of *A. ervi* and *L. fabarum* contained the same genes and the same genes were duplicated in each, implying duplication events occurred prior to the split from their most recent common ancestor. One of these duplicated genes appears to be duplicated again in *A. ervi*, or the *L. fabarum* copy has been lost.

### Chemosensory genes

Genes underlying chemosensory reception play important roles in parasitoid mate and host localization [93, 94]. Several classes of chemosensory genes were annotated separately (Table 3). With these manual annotations, further studies can now be made with respect to life history characters including reproductive mode, specialization on aphid hosts, and mimicry.

#### Chemosensory: soluble proteins (OBPs and CSPs)

Odorant-binding proteins (OBPs) and chemosensory proteins (CSPs) are possible carriers of chemical molecules to sensory neurons. Hymenoptera have a wide range of known OBP genes, with up to 90 in *N. vitripenis* [95]. However, the numbers of these genes appear to be similar across parasitic wasps, with 14 in both species studied here and 15 recently described in *D. alloeum* [33]. Similarly, CSP numbers are in the same range within parasitic wasps (11 and 13 copies here, Table 3). Interestingly, two CSP sequences (one in *A. ervi* and one in *L. fabarum*) did not have the conserved cysteine motif, characteristic of this gene family. Further work should investigate if and how these genes function.

#### Chemosensory: odorant receptors (ORs)

Odorant receptors (ORs) are known to detect volatile molecules. In total, we annotated 228 putative ORs in *A. ervi* and 156 in *L. fabarum* (Table 3). This is within the range of OR numbers annotated in other hymenopteran parasitoids, including: 79 in *M. cingulum* [96], 225 in *N. vitripennis* [97], and 187 in *D. alloeum* [33]. Interestingly, we annotated a larger set of ORs in *A. ervi* than in *L. fabarum*. One explanation is that *A. ervi* generally has more annotated genes than *L. fabarum*, and whatever broad pattern underlies the reduction in the gene repertoire of *L. fabarum* also affected OR genes. Another possibility is that the switch to an asexual reproduction has also led to a reduction in the number of OR genes, because pheromones linked to mate finding, recognition and courtship behavior are no longer necessary in an asexually reproducing species.

#### Chemosensory: ionotropic chemosensory receptors (IRs)

Ionotropic receptors (IRs) are involved in both odorant and gustatory molecule reception. In total, we annotated 38 putative IRs in *A. ervi* and 37 in *L. fabarum* (Table 3). Three putative co-receptors (IR 8a, IR 25a and IR 76b) were annotated in both species, one of which (IR 76b) was duplicated in *A. ervi*. This brings the total for the IR functional group to 42 and 40 genes for *A. ervi* and *L. fabarum*, respectively. This is within the range of IRs known from other parasitoid wasps such as *Aphidius gifuensis* (23 IRs identified in antennal transcriptome, Braconidae) [98], *D. alloeum* (51 IRs, Braconidae) [33] and *N. vitripennis* (47 IRs, Pteromalidae) [97]. A phylogenetic analysis of these genes showed a deeply rooted expansion in the IR genes (Supplementary Figure 29). Thus, in contrast to the expansion usually observed in hymenopteran ORs compared to other insect orders, IRs have not undergone major expansions in parasitic wasps, which is generally the case for a majority of insects with the exception of Blattodea [99].

### Sex determination

The core sex determination genes (*transformer*, *doublesex*) are conserved in both species (Supplementary Table 17, Additional file 13). Notably, *A. ervi* possesses a putative *transformer* duplication. This scaffold carrying the duplication (scaffold2824) is only fragmentary, but a *transformer* duplicate has also been detected in the transcriptome of a member of the *A. colemani* species complex, suggesting a conserved presence within the genus [11]. In *A. ervi*, *transformer* appears to have an internal repeat of the CAM-domain, as is seen in the genus *Asobara* [100]. In contrast, there is no evidence of duplication in sex determination genes in *L. fabarum*. This supports the idea that complementary sex determination (CSD) in sexually reproducing *L. fabarum* populations is based on up-stream cues that differ from those known in other CSD species [101], whereas the CSD locus known from other hymenopterans is a paralog of *transformer* [102].

In addition to the core sex determination genes, we identified homologs of several genes related to sex determination (Supplementary Table 18). We identified *fruitless* in both genomes, which is associated with sex-specific behavior in taxa including *Drosophila* [103]. Both genomes also have homologs of *sex-lethal* which is the main determinant of sex in *Drosophila* [104], but not other insects. *Drosophila* has two homologs of this gene, and the single version in Hymenoptera may have more in common with the non-sex-lethal copy, called *sister-of-sex-lethal*. We identified homologs of the gene *CWC22*, including a duplication in *A. ervi*; this duplication is interesting because a duplicated copy of *CWC22* is the primary signal of sex determination in the house fly *Musca domestica* [105]. Lastly, there was a duplication of *RBP1* in both genomes. The duplication of *RBP1* is not restricted to these species, nor is the duplication of *CWC22*, which appears sporadically in Braconidae. Together, these annotations add to our growing knowledge of duplications of these genes and provide possibilities for further examinations of the role of duplications and specialization in association with sex determination.

### DNA methylation genes

DNA methyltransferase genes are thought to be responsible for the generation and maintenance of DNA methylation. In general, DNA methyltransferase 3 (*DNMT3*) introduces de novo DNA methylation sites and DNA methyltransferase 1 (*DNMT1*) maintains and is essential for DNA methylation [106, 107]. A third gene, *EEF1AKMT1* (formerly known as *DNMT2*), was once thought to act to methylate DNA but is now understood to methylate tRNA [107]. In both *A. ervi* and *L. fabarum*, we successfully identified homologs *DNMT3* and *EEF1AKMT1*. In contrast, *DNMT1* was not detected in either species (Table 4, Supplementary Table 19).

**Table 4:** Summary of annotation of putative DNA methylation genes

Species	Gene	Scaffold	e-value (*Nasonia*)
*A. ervi*	EEF1AKMT1 homolog	scaffold94	1.00E-66
*L. fabarum*		tig00000449	5.00E-63
*A. ervi*	DNA methyltransferase 3	scaffold45	5.00E-138
*L. fabarum*		tig00002022	9.00E-117
*A. ervi*	DNA methyltransferase 1	*no homolog detected*	
*L. fabarum*		*no homolog detected*	

This adds to growing evidence that these genes are not conserved across Braconidae, as *DNMT1* appears to be absent in several other braconid taxa, including *Asobara tabida*, *A. japonica*, and *F. arisanus* [108, 109]. However, *DNMT1* is present in some braconids, including *M. demolitor* and *Cotesia vestalis*, and outside of Braconidae these genes are otherwise strongly conserved across insects [109]. In contrast, DNMT3, present here, is more often lost in insects [107].

This absence of *DNMT1* helps to explain previous estimates of very low DNA methylation in *A. ervi* (0.5%) [108]*.* We confirmed these low levels of methylation in *A. ervi* by mapping previously generated bisulfite sequencing data (Supplemental Figure 30) [108] to our genome assembly. We aligned > 80% of their data (total 94.5Mbp, 625,765 reads). The sequence coverage of this mapped data was low: only 63,554 methylation-available cytosines were covered and only 1216 were represented by two or more mapped reads. Nonetheless, of these mapped cytosines, the vast majority (63,409) were never methylated, just 143 sites were always methylated, and two were variably methylated. Methylation-available cytosine classes were roughly equally distributed among three cytosine classes (CG: 0.154%, CHG: 0.179%, and CHH: 0.201%). This methylation rate is less than the 0.5% estimated by Bewick et al. 2017 [108] and confirms a near absence of DNA methylation in *A. ervi*. Given the parallel absence of DNMT1 in *L. fabarum*, it seems likely that both species sequenced here may have very low levels of DNA methylation, and that this is not a significant mechanism in these species.

This stark reduction in DNA methylation is interesting, given that epigenetic mechanisms are likely important to insect defenses, including possible responses to host endosymbiont-dependent mechanisms [110–112]. As with the immune pathways discussed above, this could reflect a loss that is an adaptive response to developing within endosymbiont-protected hosts. It is also interesting that while one epigenetic mechanism seems to be absent in both *A. ervi* and *L. fabarum*, we see an increase in histone variants in *L. fabarum* (based on the OMA analysis of gene family expansion), and these histones could function in gene regulation. However, whether there is a functional or causal link between these two observations is yet to be tested.

## Discussion

We have used two new, high quality genome assemblies to investigate the basis of infectivity and specificity in two parasitoid wasps that infect aphids. Within this, we have found more predicted genes in *A. ervi* than in *L. fabarum* (Tables 1 and 2). Comparisons with other parasitoids suggest that the lower number of predicted genes in *L. fabarum* is more likely due to their loss than to a gene gain in *A. ervi*. However, it is important to recognize that predictive annotation is imperfect and any missing genes should be specifically screened with more rigorous methods. Importantly, we found relatively high BUSCO scores in the predicted genes, suggesting that our gene prediction was largely successful and was not impacted by the low GC-content. One contribution to the overall difference in gene numbers could be in the larger number of orphan genes that were identified in *A. ervi*. The evolutionary origin of these orphan genes is not known [113, 114], but their retention or evolution could be important to understanding specific functions or traits in this taxon.

The two genomes contained different patterns of predicted TE content. The spread of reported TE coverage in arthropods is quite large, even among *Drosophila* species (ca. 2.7–25%) [115], and variation in genome size has been broadly attributed to TE content [35, 116]. The variation we observe here suggests that differences in predicted TE content may be evolutionary quite labile, even within closely related species with the same genome size. However, this could also be a consequence of the assembly methods, and this should be further studied.

### GC content

The GC content of these two parasitoids is lower than any insect sequenced to date. This bias is accompanied by very strong codon bias (Fig. 2), which has functional consequences in terms of both expression efficiency and mRNA stability [117]. There is reason to expect that environment could contribute to the low GC content of these genomes; in taxa including bacteria [118] and plants [119] the environment has been shown to influence GC content via limitation in elements such as nitrogen. These two wasps parasitize aphids exclusively, and aphids themselves have relatively low genome-wide GC content, perhaps related to their high-sugar, low-nitrogen, sap diet. This includes the pea aphid (*Acyrthosiphon pisum,* Additional file 6), which is a frequent host of *A. ervi* and also has notably low GC content (29.8%) [120]. We did not observe a relationship in codon usage nor nitrogen content between wasps and other taxa in this system (i.e. aphids and their symbionts, Supplementary Figures 10–12). This suggests that the environment does not constrain these genomes in a way that drives lower GC content in these two parasitoids. However, it seems plausible that they were broadly limited by the environment, and that GC content was further constrained by other factors.

GC content in these two genomes could be low because of their relatively small size. Genome size and GC content are positively correlated in a diverse set of taxa including bacteria [121, 122], plants [119, 123], and vertebrates [124]. This widespread pattern may be driven by GC-rich repetitive elements that are more abundant in larger genomes, stronger selection on thermal stability in larger genomes, or thermal stability associated with the environment [119, 124]. Additionally, the apparent lack of DNA methylation in this system may contribute to low GC content (see below, and [108]). Methylation is a stabilizing factor with regard to GC content [125], so its absence could relax selection on GC content and allow it to decline. However, neither the absence of methylation nor codon bias are unique to these taxa, suggesting that some additional selective factors or genetic drift may have further shaped the composition of these two genomes.

Our finding that genes with adult-biased expression have lower GC content suggests that selection at different life-history stages may help shape genome content. Although the magnitude of these differences is not very large, subtle differences in gene content are hypothesized to be the result of selection in other systems [126]. It seems plausible that GC content differences among genes expressed at different life history stages could be selected in a process analogous to the small changes in gene expression that are linked to large phenotypic differences within and between species [127]. Lower GC content in adult-biased genes could be the consequence of differing energy demands and availability of resource across life stages. Expressing genes that best match their biased codon usage should be more efficient and accurate, resulting in lower energy consumption and faster turnover [128–131]. Expressing AT-rich genes is slightly more energy-efficient in itself, and this could favor otherwise neutral mutations from GC to AT [132]. There is good motivation for adults to have a greater demand for energy efficiency. Adult parasitoids usually feed on carbohydrate rich but protein and lipid poor resources like nectar, while performing costly tasks including flying, mating, and laying eggs. Meanwhile, parasitoid larvae are feeding on their aphid host’s tissue, and likely benefit further from nutrients coming from the aphids’ endosymbionts, while their only task is to grow as fast as possible [133–135].

Further explanations to be considered for low GC content include effective population size, translational efficiency, and mutational bias [131, 136, 137]. Although we do not have the power to test for GC-biased gene conversion with two taxa, the even lower third position GC content (*A. ervi*: 15.5% and *L. fabarum*: 10.7%, Table 1) suggests that this should be tested in relation to other parasitoids [131]. Altogether, these patterns raise important questions about how codon biases impact genome content, and whether synonymous mutations are always functionally neutral [73, 138].

### Gene family expansions

The most striking result of our gene family comparison was the expansion of F-box/*LRR* proteins in *A. ervi* relative to *L. fabarum* (232 vs. 68 genes across four orthologous groups, Supplementary Figure 16). The *LRR*s are a broad class of proteins associated with protein-protein interactions, including putative venom components in these parasitoids [24]. *LRRs* belong to a larger category of leucine rich repeat pattern recognition receptor proteins, which are an important component of innate immunity and cell-surface recognition of bacterial intruders and include Toll-like receptors in insects [139, 140]. While the functions of these proteins are diverse, expansion in F-box/*LRR* proteins has been shown to have specific function in immunity in parasitic insects. In the Hessian fly (*Mayetiola destructor*), fly-encoded F-box/*LRR* proteins bind with plant-encoded proteins to form a complex that blocks the plant’s immune defenses against the parasitic fly [141]. Thus, we hypothesize that the expansion of this class of proteins in both species is related to recognizing the diverse bacterial defenses of their broad aphid hosts. We argue that expansion of F-box/*LRR* proteins in *A. erv*i relative to *L. fabarum*, may be associated with a recent arms race with respect to the immune defenses and protective endosymbionts of their host aphids.

Evidence for expansion of histone genes in *L. fabarum* may also have important functional consequence. All eukaryotic genomes examined to date contain multiple histone genes for the same histone variants found in humans (e.g. 22 genes for H2B or 16 genes for H2A in humans) [142], and it has recently been suggested that these histone variants are not functionally equivalent but rather play a role in chromatin regulation [142]. Hence, these variants could also play a role in several *L. fabarum* specific traits, including the switch from sexual to asexual reproduction (thelytoky); in mammals, sex determination has been linked to regulation via histone modification [143]. Alternatively, the expansion of histone genes could be related to their rapid development in hosts, as has been suggested for similar histone expansions in the parasitoid *Diadromus collaris* [32].

### Venom

Venom injected at oviposition is crucial for successful reproduction in most parasitoid wasp species [23, 144]. Although these two species differ in their host range [40], comparison of venom proteins between species revealed that more than 50% of the proteins are shared between species (Fig. 4). Glutamyl transpeptidases are the most abundant proteins in the venom of both *A. ervi* and *L. fabarum*. Phylogenetic analysis suggests that they are the result of the duplication of a gene encoding a “classical” glutamyl transpeptidase prior to the separation of the two species (Fig. 5). These venom proteins have been suggested to be involved in the castration of the aphid host after parasitism [145]. *LRR* proteins, which were previously reported in the venom of *A. ervi* [24] and other Braconidae [146], are likely important for evading the hosts immune response.

### Key gene families

We manually annotated more than 1000 genes (719 for *A. ervi* and 644 for *L. fabarum*, Table 3) from functional categories that are key to parasitoid life history and adaptation. This is especially important for large gene families, which are usually poorly annotated by automatic prediction [147]. Expansion and reductions in such gene families potentially underlie key adaptive differences between the two parasitoids, and necessitate accurate annotation.

#### Desaturases

Desaturases are an important gene family that introduce carbon-carbon double bonds in fatty acyl chains in insects [148, 149]. While these function broadly across taxa, a subset of these genes (specifically acyl-CoA desaturases) have been implicated in insect chemical recognition for roles including alkene production and modification of fatty acids [150]. This gene family is particularly interesting because it has been shown that *Lysiphlebus cardui*, a close relative of *L. fabarum* [38]*,* have no unsaturated cuticular hydrocarbons (CHCs), just as is seen in its aphid host. This allows the parasitoid to go undetected in aphid colonies that are ant-tended and therefore better parasitize them [56]. We showed that the CHC profile of *L. fabarum* is also missing alkens and alkadiens (Supplementary Figure 21)*.* In contrast, *A. ervi* produces a large amount of unsaturated hydrocarbons (Supplementary Figures 22 and 23). The loss of three annotated desaturase genes in *L. fabarum* compared to *A. ervi* might explain these differences in the composition of their CHC profiles, especially their apparent inability to synthesize dienes. Further work is needed to verify these losses in *L. fabarum*, identify the orthologs of the missing copies in *A. ervi*, and test if these potentially lost desaturase genes in *L. fabarum* are involved in the generation of unsaturated CHCs in *A. ervi*.

#### Immune genes

We found that some genes from the Imd and Toll pathways are absent from the genomes of *A. ervi* and *L. fabarum*, possibly including the gene *imd*, which has been present in other hymenopteran genomes analyzed to date. Strikingly, all of the genes in the Imd pathway, including those encoding GNBP- and PGRP, *imd*, *FADD*, *Dredd* and *Relish* are missing in some aphid genomes (*Acyrthosiphon pisum, Aphis gossypii* and *Diuaphis noxia*) [151, 152], and *imd* is absent in the genomes of *A. glycines*, *M. persicae, M. cerisae,* and *R. padi*, some of which are hosts for *A. ervi* and *L. fabarum* [40]. The lack of an Imd pathway in aphids has been suggested to be an adaptation to tolerate the obligate bacterial symbiont, *Buchnera aphidicola*, as well as their facultative endosymbionts that are mostly gram-negative gamma-proteobacteria (e.g. *Hamiltonella defensa*). Some of these facultative symbionts exhibit defensive activities against microbial pathogens and insect parasitoids [30, 153–155] and may at least partially compensate for innate immune functions in aphids. A recent study has demonstrated the absence or incompleteness of the Imd pathway in some other Hemiptera species, suggesting repeated and independent losses of components of this pathway [156]. These authors also suggest that cross-talk could occur between the Imd and Toll pathways to target wider and overlapping arrays of microbes [156]. Whether similar cross-talk occurs in these two Aphidiidae (*A. ervi* and *L. fabarum*) warrants further study. Further work investigating this should formally verify their loss, as our lack of detection could be due to low homology or assembly errors.

Overall, our results suggest a possible convergent loss of some immunity genes, and possibly immune function, between these parasitoids and their aphid hosts. One reason could be that parasitoid larvae require basic nutrients during the early stages of development, which are supplied by the host and its symbionts, and thus an immune response from the parasitoid larvae might impair this function. Alternatively, but not exclusively, mounting an immune response against bacteria by the parasitoid larvae may be energetically costly and divert resources from its development. This idea of energy conservation would be especially relevant if the GC-reduction in these genomes is a consequence of energy conservation. It is still unclear whether other unrelated aphid parasitoids lack *imd*, as well as the upstream activators and downstream effectors of the immune pathways. This altered immunity might lead to either a decrease in the wasps’ responses to pathogenic bacteria, or they may use other defensive components to fight bacterial infections (perhaps some in common with aphids) that are yet to be discovered. For example, in *L. fabarum* recent transcriptomic work has shown that detoxifying genes may be a key component of parasitoid success [157], and these could play a role in immunity.

## Conclusions

These two genomes have provided insight into adaptive evolution in parasitoids that infect aphids. Both genomes are extremely GC-poor, and the accompanying codon bias provides an excellent system for examining the chemical biases and selective forces that may overshadow molecular evolution in eukaryotes. We have also highlighted several groups of genes that are key to functional evolution across insects, including venom, sex determination, response to bacterial infection (F-box/*LRR* proteins), and near absence of DNA methylation. Moreover, the absence of certain immune genes (e.g. from the Imd and Toll pathways) in these two species is similar to losses in host aphids, and raises intriguing questions related to the effects of aphids’ symbiosis on both aphid and parasitoid genomics. Lastly, differences between the two species including their OR gene repertoires and missing desaturase genes could help explain fundamental differences in their life history like the switch from sexual to asexual reproduction or aphid mimicry in *L. fabarum*.

Parasitoid wasps provide an excellent model for studying applied and basic biological questions, including host range (specialist vs generalist), reproductive mode (sexual vs asexual), antagonistic coevolution, genome evolution, and epigenetic regulation, to mention just a few. Our new genomic resources will open the way for future research, including work to understand mechanisms underlying host specialization, and adaptive changes associated with climate change [158, 159]. Lastly, the genomes of these two non-social Hymenoptera provide a valuable comparison for understanding processes specific to social insects with complex caste structure, and are a first but essential step to better understand the genetic architecture and evolution of traits that are important for a parasitic life style and their use in biological control.

## Methods

*More complete methods are available in the Supplementary Material (Additional file *14**)*.

### Insect collection and origin

#### Aphidius ervi

*Aphidius ervi* samples used for whole-genome sequencing came from two different, sexually reproducing, isofemale lines established from parasitized aphids (recognizable as mummies) from fields of cereals and legumes in two different geographic zones in Chile: Region de Los Rios (S 39° 51′, W 73° 7′) and Region del Maule (S 35° 24′, W 71° 40′). Mummies (parasitized aphids) of *Sitobion avenae* aphids were sampled on wheat (*Triticum aestivum* L*.*) while mummies of *Acyrthosiphon pisum* aphids were sampled on *Pisum sativum* L. (pea aphid race). Aphid mummies were isolated in Petri dishes until adult parasitoids emerged. These two parasitoid lineages were separated in two cages with *H. defensa*-free hosts* ad libitum* and were propagated for approximately 75 generations under controlled conditions as described elsewhere [160, 161]. A further reduction of genetic variation was accomplished by establishing two isofemale *A. ervi* lines, which were maintained as described previously and propagated for approximately 10 generations before adult parasitoids (male and female) were collected live and stored in 1.5ml centrifuge tubes containing ethanol (95%) at − 20 °C.

*Aphidius ervi* samples used for CHC analysis (below) were purchased from Katz Biotech AG (Baruth, Germany). Species identification was confirmed with COI barcoding following Hebert et al. [162]. Wasps sacrificed for CHC analysis were sampled from the first generation reared in the lab on *Acyrthosiphon pisum* strain LL01 [163], which were mass-reared on *Vicia faba* cv. *Dreifach Weisse*.

#### Lysiphlebus fabarum

*Lysiphlebus fabarum* samples used for whole-genome sequencing came from a single, asexually reproducing, isofemale line (IL07–64). This lineage was first collected in September 2007 from Wildberg, Zürich, Switzerland (47°25′31″N 8°49′00″E) as mummies of the aphid *Aphis fabae fabae*, collected from the host plant *Chenopodium album*. In the lab, parasitoids were reared on *H. defensa*-free *A. f. fabae* raised on broad bean plants (*Vicia faba*) under controlled conditions [16 h light: 8 h dark, 20 °C] until sampling in September 2013, or approximately 150 generations. Every lab generation was founded by ca. 10 individuals that were transferred to fresh host plants containing wasp-naïve aphids. Approximately 700 individuals were collected for whole-genome sequencing from a single generation in December 2013 and flash frozen at − 80 °C. To avoid sequencing non-wasp DNA, samples were sorted over dry ice to remove any contaminating host aphid or plant material.

For linkage group construction, separate *L. fabarum* collections were made from a sexually reproducing lineage. Here, we collected all sons produced by a single virgin female, sampled from the control lineage in a recently employed evolution experiment (*Hamiltonella defensa*-free lineage) [42]. Wasps were stored on ethanol until RAD-seq library construction. Lastly, a third population was sampled for the proteomic analysis of the venom-apparatus (below); these females came from the genetically-diverse starting population used to found the evolution experiment of Dennis et al. [42], and were sampled in December 2014.

### DNA extraction and library preparation

#### Aphidius ervi

DNA was extracted from adult haploid males of *A. ervi* in seven sub-samples (ca. 120 males each), reared on *S. avenae*. Total DNA was extracted using the DNEasy Plant Mini Kit (QIAGEN) following the manufacturer’s instructions. DNA was quantified by spectrophotometry (Epoch Microplate Spectrophotometer, Biotek) and fluorometry (Qubit 3.0; Qubit DNA High sensitivity Assay Kit, Invitrogen), and quality was assessed using 1% agarose gel electrophoresis. DNA samples were sent on dry ice to MACROGEN (Seoul, South Korea) and were used to produce Illumina paired-end (PE) and mate-pair (MP) libraries for sequencing. A PE library was constructed from one of the seven sub-samples (120 individuals, 1 μg DNA) sheared by ultrasonication (Covaris) company, average sheared insert size: 350 bp). The remaining DNA samples were pooled (6 samples, 720 individuals) and used for MP sequencing (3 kb, 5 kb and 8 kb insert sizes), which were prepared with the Nextera mate-pair protocol (Illumina). All libraries were sequenced using an Illumina HiSeq 2000 sequencer (MACROGEN).

Long read PacBio (Pacific Biosciences) RS II sequencing was performed from a single DNA extraction of 270 *A. ervi* females, reared on *A. pisum*. Genomic DNA was extracted using the Wizard genomic DNA purification kit (Promega) according to manufacturer instructions and quantified spectrophotometrically using a NanoDrop 2000 (Thermo Scientific). Input DNA was mechanically sheared to an average size distribution of 10Kb (Covaris gTube, Kbiosciences) and the resulting library was size selected on a Blue Pippin Size Selection System (Cat #BLU0001, Sage Science) to enrich fragments >8Kb. Quality and quantity were checked on Bioanalyzer (Agilent Technologies) and Qubit, respectively. Four SMRT RSII cells with P6 chemistry were sequenced at GenoScreen, France.

#### Lysiphlebus fabarum

DNA was extracted from adult female *L. fabarum* in 10 sub-samples (50–100 wasps each) using the QIAmp DNA mini Kit (Qiagen) according to the manufacturer’s instructions, with the inclusion of an overnight tissue digestion at 56 °C. Extracted DNA was then pooled and used to produce Illumina PE and MP, and PacBio libraries. The PE library was prepared using the Illumina Paired-End DNA protocol; the average fragment size was 180 base pairs (bp). The MP library (5 kb insert) was generated with the Nextera mate-pair protocol (Illumina). Both libraries were sequenced on the Illumina MiSeq in Paired-End mode at the Functional Genomics Center Zürich.

Long-read libraries for PacBio RS II sequencing were produced using the DNA Template Prep Kit 2.0 (Pacific Biosciences). Input DNA was mechanically sheared to an average size distribution of 10Kb (Covaris gTube, Kbiosciences) and the resulting library was size selected on a Blue Pippin Size Selection System (Sage Science) machine to enrich fragments >8Kb; quality and quantity were checked on the Bioanalyzer and Qubit, respectively. Ten SMRT Cells were sequenced at the Functional Genomics Center Zürich.

### Genome assembly

#### Aphidius ervi

Library quality was checked with FastQC ver. 0.11.3 [164]. Paired-end libraries were processed with Trimmomatic ver. 0.35 [165] for trimming Illumina adapters/primers, low quality bases (Q < 25, 4 bp window) and discarding sequences shorter than 50 bp or without its mate-pair. In the case of Mate-Pair libraries, removal of improperly oriented read-pairs and removal of Nextera adapters was performed using NextClip [166]. Filtered PE and MP libraries were used for genome assembly with Platanus ver. 1.2.1 with default parameters [167], gap closing was performed with GapCloser [168]. Scaffolding with PacBio reads was performed using a modified version of SSPACE-LR v1.1 [169], with the maximum link option set by –a 250. Finally, the gaps of this last version were filled with the Illumina reads using GapCloser.

#### Lysiphlebus fabarum

Library quality was also checked with FastQC [164]. Illumina reads were filtered using Trimmomatic ver 0.33 to remove low quality sequences (Q < 25, 4 bp window), to trim all Illumina primers, and to discard any sequence shorter than 50 bp or without its mate-pair. NextClip was used to remove all improperly oriented read pairs.

Raw PacBio reads were error-corrected using the quality filtered Illumina data with the program Proovread [170]. These error-corrected reads were then used for de novo assembly in the program *canu* v1.0 [171]. Since our PacBio reads were expected to have approximately 30X coverage (based on the presumed size of 128Mbp), *canu* was run with the recommended settings for low coverage data (corMhapSensitivity = high corMinCoverage = 2 errorRate = 0.035), and with the specification that the genome is approximately 128Mbp. The resulting assembly was polished using a single iteration of Pilon [172] to correct for both single nucleotide and small indel errors, using mapping of both the MP and PE data, generated with bwa-mem [173].

### Linkage map construction in *L. fabarum*

For linkage map construction, we followed the methodology described in Wang et al. [174] and Purcell et al. [175]. In brief, we genotyped 124 haploid male offspring from one sexual female using ddRADseq. Whole-body DNA was high-salt extracted [176], digested with the *EcoRI* and *MseI* restriction enzymes, and ligated with individual barcodes [177, 178]. Barcoded samples were purified and amplified with Illumina indexed primers by PCR [178] and quality-checked on an agarose gel.

Pooled samples were sequenced on the Illumina HiSeq2500. Raw single-end libraries were quality filtered and de-multiplexed using the process_radtags routine within Stacks v1.28 with default parameters [179], and further filtered for possible adapter contamination using custom scripts. Genotyping was performed by mapping all samples against the *L. fabarum* draft genome assembly using bowtie2 [180] with rg-id, sensitive and end-to-end options. Genotypes were extracted using samtools mpileup [181] and bcftools (haploid option) [182]. We filtered the resulting genotypes for a quality score > 20 and removed loci with > 20% missing data and/or a minor allele frequency < 15% using VCFtools v0.1.12b [183]. After filtering, 1319 biallelic SNPs in 90 offspring remained.

For constructing linkage groups, we followed Gadau 2009 [184] to account for the unknown phase of the maternal genotype. In short, we duplicated the haploid male genotypes and reversed the phase for one duplicated set and removed one of the mirror linkage group sets after mapping. We generated the map using MSTmap [185] on the data with following parameters: population_type DH, distance_function kosambi, no_map_dist 15.0, no_map_size 2, missing_threshold 1.00, and the cut_off_p_value 1e-6. The cut-off *p*-value was adjusted to create a linkage map of five linkage groups, however the biggest group had a gap of > 70 cM, indicating a false fusion of two groups, which we split in two groups. This result corresponded to the six chromosomes previously described for *L. fabarum* [52], these were visualized with AllMaps [186]. Initial mapping showed that 14 SNPs at one end of tig0000000 mapped to Chromosome1, while the majority of the contig (> 150,000 bp) mapped to Chromosome 2. Thus, these SNPs were removed from the linkage maps, and it is advised that subsequent drafts of the *L. fabarum* genome should split this contig around position 153,900.

### Genome completeness and synteny

Completeness of the two assemblies was assessed by identifying Benchmarking Universal Single-Copy Orthologs (BUSCOs) using the BUSCO v3.0.2 pipeline in genome mode [187]. We identified single copy orthologs based on the insecta_db9 (1066 genes, training species: *Nasonia vitripennis*).

Synteny between the two genomes was assessed using the NUCmer aligner, which is part of the MUMmer v3.23 package [66]. For this, we used the *L. fabarum* chromosomes as the reference, and included the scaffolds not incorporated into chromosomes (total 1407 pieces). The *A. ervi* assembly was mapped to this using the default settings of NUCmer.

### Predictive gene annotation

For both assembled genomes, gene predictions were generated using MAKER2 [188]. Within MAKER2, predictive training was performed in a three step process. A first set of genes was predicted by similarity to known proteins or contigs from RNAseq in the same species (described below). This gene set was used thereafter for training both Augustus [189] and SNAP [190], in two steps, with the results of the first training re-used to train the software in the second round. Transcriptomic evidence was provided separately for each species. For *A. ervi*, six separate de novo transcriptome assemblies from Trinity [191] were constructed, one each for the adults reared on different hosts (NCBI PRJNA377544) [160]. For each transcript, we only included variants based on filtering with RSEM v 1.2.21 using the option –fpkm_cutoff 1.0, −-isopct_cutoff = 15.00. This resulted in 452,783 transcripts. For *L. fabarum*, we utilized a joint transcriptome, built using RNAseq data (NCBI PRJNA290156) collected from adults [42] and 4–5 day old larvae [157]. Peptide evidence came from the Hymenoptera genomes database (http://hymenopteragenome.org, *Acromyrmex echiniator* v3.8, *Apis mellifera* v3.2, *Nasonia vitripennis* v1.2), from the BioInformatics Platform of Agroecosystems Arthropod database (https://bipaa.genouest.org, *Hyposoter didymator* v1.0), and *Drosophila melanogaster* (http://flybase.org, v6.13), and SwissProt (October 2016) databases. Summary statistics were generated with GAG [192]. Transcriptomic support for the predicted genes was estimated by mapping available transcriptomic data (same as above) to the respective genomes using STAR [193] in the “quantMode”.

### Contamination filtering

We screened for contamination in two steps. First, we used blobtools [64] to examine scaffolds based on sequencing coverage (from the paired-end reads mapped with bwa-mem), GC content, and top BLAST hit (cutoff 1e-25). We further screened the predicted genes to assign them to either host aphid (*Acyrthosiphon pisum* and *Aphis glycines*) or to the other parasitoid. Scaffolds that did not match to Arthropoda, and predicted genes that matched to aphid were manually examined. In both cases, we retained genes and regions with no known match, as these warrant future investigation.

### Functional annotation

The putative functions of the proteins predicted by the above pipeline were identified based on BLASTp (v2.5.0) matches against Genbank *nr* (non-redundant GenBank CDS translations+PDB + SwissProt+PIR + PRF) release 12/2016 and interproscan v5 against Interpro (1.21.2017). GO terms associations were collected from BLAST *nr* and interproscan results with blast2GO (v2.2). Finally, transmembrane domains were identified with Hidden Markov Models (HMM) in tmhmm v2.0c, and peptide signals with signal (euk v4.1) [194, 195].

### Transposable elements

Transposable elements (TE) were predicted using the REPET pipeline [196], combining de novo and homology-based annotations. Repetitive elements were identified de novo across all scaffolds larger than the scaffold N50 for each species, following recommendations from the developers of REPET for draft genome assemblies. Within these, repetitive elements were identified using a BLAST-based alignment of each genome to itself followed by clustering with Recon [197], Grouper [198] and Piler [199]. For each cluster, a consensus sequence was generated by multiple alignment of all clustered elements with MAP [200]. The resulting consensus was then scanned for conserved structural features or homology to nucleotide and amino acid sequences from known TEs (RepBase 20.05) [201, 202] using BLASTER (tblastx, blastx) [196] or HMM profiles of repetitive elements (Pfam database 27.0) using hmmer3 [203]. Based on identified features, repeats were classified using Wicker’s TE classification as implemented in the PASTEclassifier [204]. The resulting de novo TE library for the genome was then filtered to retain only the elements with at least one perfect match in the genome. Subsequently, all TEs in the full genomes were annotated with REPET’s TE annotation pipeline. Reference TE sequences were aligned to the genome using BLASTER, Repeat Masker [205] and CENSOR [206]. The resulting HSPs were filtered using an empirical statistical filter implemented in REPET [196] and combined using MATCHER [198]. Short repeats were identified using TRF [207] and Mreps [208]. Elements in genomic sequences with homology with known repbase elements (RepBase 20.05) were identified with BLASTER (BLASTx, tBLASTx) and curated by MATCHER. Finally, redundant TEs and spurious SSR annotations were filtered and separate annotations for the same TE locus were combined using REPET’s “long join procedure”. DNApipeTE [209] was run to estimate repeat content from unassembled raw reads. For this, we first sampled 1,000,000 paired-end reads (100 bp) from each species and filtered these for adapter contamination with Trimmomatic 0.39. DNApipeTE was run with “-sample_number 3“, “-genome_coverage 0.5 “against the arthropod-specific RepeatMasker library.

### GC content and codon usage

We examined several measures of nucleotide composition, at both the nucleotide and protein level. Whole genome GC content was calculated by totaling the numbers of A, C, T, and G in the entire assembly. In the predicted coding sequences, this was also calculated separately for each predicted gene and third position GC composition was calculated separately in the predicted coding sequences. In all cases, this was done with the sscu package in R [210]. Relative Synonymous Codon Usage (RSCU) was extracted from the entire CDS using the seqinR package in R [211], and visualized with a PCA (R packages factoextra, reshape, and ggplot2) [212–214]. To examine GC content in coding genes of other insects, we downloaded the 118 available CDS in the RefSeq database of NCBI (date: October 2018) and again calculated per-gene GC content.

To examine the GC content of life-stage biased transcripts, we compared GC content in the genes that were significantly differentially expressed between adults and larvae, and in the most highly expressed genes in this data. We utilized previously generated transcriptomes from 43 pools of adult females [42] and 24 individual larval [157] *L. fabarum*. Differential expression was calculated using DESeq2 [215], and genes with an FDR <0.05 were deemed significantly differentially expressed. The full models of expression accounted for aphid host and replicate; the full analysis pipeline is detailed in the Supplemental Materials. GC content between these pools of genes was compared with a two-sided t-test, implemented in R.

### Orphan genes

We identified orphan genes as those for which we could not find orthologs in any other sequenced genomes (Supplementary Table 11). To do this, we first used OrthoFinder [216] to generate clusters of orthologous and paralogous genes among the predicted genes (CDS) from the genomes of *A. ervi* and *L. fabarum*, as well as five other sequenced parasitoids (*Diachasma alloeum*, *Fopius arisanus*, *Macrocentrus cingulum*, *Microplitis demolitor* and *Nasonia vitripennis*). OrthoFinder produces a set of genes that were not assigned to any orthogroup. We identified species specific genes, which we are calling orphan genes, by removing all genes that had hits to any other genes in the *nt*, *nr*, and *swissprot* NCBI database (June 2019). Within these putative orphans, we only retained those with transcriptomic support.

### Gene family expansions

We examined gene families that have expanded and contracted in *A. ervi* and *L. fabarum* relative to one another using the OMA standalone package (v2.2.0, default values) [74]. OMA was used to compute orthologs (OMA groups) and Hierarchical Orthologous Groups (HOGs) for the predicted proteins of *L. fabarum* (OGS1) and *A. ervi* (OGS3): 15,203 and 20,344, respectively. While OMA groups consist of strict 1:1 orthologs between OGS1 and OGS3, HOGs contain all orthologs and paralogs of a given predicted gene family. HOGs were parsed with a custom Perl script to identify all gene families in which one of the wasp species contained more members than the other. We focused on only the groups that contained more than 20 genes (10 groups, Supplementary Figure 15). These were identified by BLASTx against the *nr* database in NCBI.

### Venom proteins

The *L. fabarum* venom proteomic analysis was performed from 10 extracted venom glands (Supplementary Figure 17). The 16 most visible bands in 1D gel electrophoresis were cut, digested with trypsin and analyzed by mass spectrometry. All raw data files generated by mass spectrometry were processed to generate mgf files and searched against: (i) the *L. fabarum* proteome predicted from the genome (*L. fabarum* annotation v1.0 proteins) and (ii) the *L. fabarum* de novo transcriptome [42] using the MASCOT software v2.3 [217]. The mass spectrometry proteomics data are deposited in the ProteomeXchange Consortium (proteomecentral.proteomexchange.org) via the PRIDE partner repository [62], with the ID PXD015758.

Sequence annotation was performed based on BLAST similarity searches. Signal peptide prediction was performed with SignalP [194, 195]. Searches for protein domains was performed with PfamScan [218] and venom protein genes were identified using the BLAST tools in Apollo [79, 81]. Multiple amino acid sequence alignments were made with MUSCLE [219, 220]. Phylogenetic analysis was performed using maximum likelihood (ML) with PhyML 3.0 [221]. SMS was used to select the best-fit model of amino acid substitution for ML phylogeny [222].

### Manual gene curation

The two genome assemblies were manually curated for a number of gene families of interest. This improved their structural and functional annotation for more in-depth analysis. Manual curation, performed in Apollo included the inspection of stop/start codons, duplications (both true and erroneous), transcriptomic support, and concordance with the predicted gene models.

### Desaturases

Desaturase genes in both genomes were automatically identified and annotated with GeMoMa [223] using desaturase gene annotations from *Diachasma alloeum*, *Fopius arisanus*, and *Microplitis demolitor*, retrieved from NCBI’s protein database as queries (retrieved May 2017). Additionally, all desaturase genes were manually inspected.

To determine the cuticular hydrocarbon (CHC) profiles in *A. ervi*, wasps were freeze-killed and stored separately by sex at − 20 °C. For CHC extraction, single individuals were covered with 50 μl of MS pure hexane (UniSolv) in 2 ml GC vials (Agilent Technologies,) and swirled for 10 min on a Thermo-shaker (IKA KS 130 Basic, Staufen). The hexane extracts where then transferred to a fresh conical 250 μl GC insert (Agilent Technologies), where the hexane was completely evaporated under a constant flow of CO_2_. The dried extract was then resuspended in 5 μl of a hexane solution containing 7.5 ng/μl of n-dodecane (EMD Millipore Corp.) as an internal standard. Three microlitre of the extract were then injected into a GC-QQQ Triple Quad (GC: 7890B, Triple Quad: 7010B, Agilent) with a PAL Autosampler system operating in electron impact ionization mode. The split/splitless injector was operated at 300 °C in Pulsed splitless mode at 20 psi until 0.75 min with the Purge Flow to Split Vent set at 50 mL/min at 0.9 min. Separation of compounds was performed on a 30 m × 0.25 mm ID × 0.25 μm HP-1 Dimethylpolysiloxane column (Agilent) with a temperature program starting from 60 °C, held for 2 min, and increasing by 50 °C per min to 200 °C, held for 1 min, followed by an increase of 8 °C per min to 250 °C, held again for 1 min, and finally 4 °C per min to 320 °C, held for 10 min. Post Run was set to 325 °C for 5 min. Helium served as carrier gas with a constant flow of 1.2 ml per min and a pressure of 10.42 psi. Initially CHC peaks were identified and the chromatogram was generated using the Qualitative Analysis Navigator of the MassHunter Workstation Software (vB.08.00 / Build 8.0.8208.0, Agilent). CHC quantification was performed using the Quantitative Analysis MassHunter Workstation Software (vB.09.00 / Build 9.0.647.0, Agilent). Peaks were quantified using their diagnostic (or the neighboring most abundant) ion as quantifier and several characteristic ions in their mass spectra as qualifiers to allow for unambiguous detection by the quantification software. The pre-defined integrator Agile 2 was used for the peak integration algorithm to allow for maximum flexibility. All peaks were then additionally checked for correct integration and quantification, and, where necessary, re-integrated manually. Percentages were based on the respective averages of four individual female CHC extracts.

### Immune genes

The list of immune genes to be searched against the *A. ervi* and *L. fabarum* genomes was established based on *Drosophila melanogaster* lists from the Lemaitre laboratory (lemaitrelab.epfl.ch/fr/ressources) [224, 225] and from the interactive fly web site [84] (www.sdbonline.org/sites/fly/aignfam/immune.htm). Each *D. melanogaster* protein sequence was used in BLAST similarity searches against the two predicted wasp proteomes (BLASTp) and against the entire assembly (tBLASTn) when no direct match was obtained. The best match was retained, and its protein sequence was used to perform a new BLAST search using the NCBI non-redundant protein sequence database to confirm the similarity with the *D. melanogaster* sequence. When both results were concordant, the retained sequence was then searched for in *Nasonia vitripennis* and *Apis mellifera* proteomes to identify homologous genes in these species.

### Osiris genes

Osiris gene orthologs were determined with a two-part approach: candidate gene categorization followed by phylogenetic clustering. Candidate Osiris genes were generated using HMM with hmmer v3.1b2 [226] and local alignment searching [227]. A custom HMM was derived using all 24 well annotated and curated Osiris genes of *Drosophila melanogaster*. Next, an HMM search was performed on the *A. ervi* and *L. fabarum* proteomes, extracting all protein models with *P* < 0.05. Similarly, all *D. melanogaster* Osiris orthologs were searched in the annotated proteomes of *A. ervi* and *L. fabarum* using protein BLAST (e < 0.05). The top BLAST hit for each ortholog was then searched within each parasitoid genome for additional paralogs (e < 0.001). All unique candidates from the above approaches were then aligned using MAFFT [228], and an approximate maximum-likelihood phylogeny was constructed using FastTree [229] via the CIPRES science gateway of Xsede [230]. The species used were: the fruit fly (*D. melanogaster*), the tobacco hornworm moth (*Manduca sexta*), the silkworm moth (*Bombyx mori*), the flour beetle (*Tribolium castaneum*), the jewel wasp (*Nasonia vitripennis*), the honeybee (*Apis mellifera*), the buff tail bumble bee (*Bombus terrestris*), the red harvester ant (* Pogonomyrmex barbatus*), the Florida carpenter ant (*Camponotus floridanus*), and Jerdon’s jumping ant (*Harpegnathos saltator*).

### OXPHOS

Genes involved in the oxidative phosphorylation pathway (OXPHOS) were identified in several steps. Initial matches were obtained using the nuclear-encoded OXPHOS proteins from *Nasonia vitripennis* [231]; J. D. Gibson unpublished] and *Drosophila melanogaster* (downloaded from www.mitocomp.uniba.it) [232]. These two protein sets were used as queries to search the protein models predicted for *A. ervi* and *L. fabarum* (blastp) [233]. Here, preference was given to matches to *N. vitripennis*. Next, genes from the *N. vitripennis* and *D. melanogaster* reference set that did not have a match in the predicted proteins were used as queries to search the genome-assembly (BLASTn), in case they were not in the predicted gene models. Gene models for all matches were then built up manually, based on concurrent evidence from the matches in both *A. ervi* and *L. fabarum* and their available expression evidence. The resulting protein models were aligned to one another and to *N. vitripennis* using MAFFT [228] to identify missing or extraneous sections. These results were used as queries to search the *N. vitripennis* proteins to ensure that all matches are reciprocal-best-BLAST-hits. Gene naming was assigned based on the existing *N. vitripennis* nomenclature. Potential duplicates were flagged based on BLAST-matches back to *N. vitripennis* (Additional file 12).

### Olfactory genes

#### Odorant-binding proteins (OBPs) and chemosensory proteins (CSPs)

To identify OBPs based on homology to known sequences, we retrieved 60 OBP amino acid sequences from other Braconidae (namely *Fopius arisanus* bw *Microplitis demolitor*) from GenBank. To this, we added seven OBPs found in a previous transcriptome of *A. ervi* (Patrizia Falabella, unpublished, EBI SRI Accessions: ERS3933807- ERS3933809). To identify CSPs, we used CSP amino acid sequences from more Hymenoptera species (*Apis mellifera*, *Nasonia vitripennis*, *Fopius arisanus* and *Microplitis demolitor*). These sets were used as query against *A. ervi* and *L. fabarum* genomes using tBLASTn (e-value cutoff 10e-3 for OBPs and 10e-2 for CSPs). Genomic scaffolds that presented a hit with at least one of the query sequences were selected. To identify precise intron/exon boundaries, the Braconidae OBP and CSP amino acid sequences were then aligned on these scaffolds with Scipio [234] and Exonerate [235]. These alignments were used to generate gene models in Apollo. Gene models were manually curated based on homology with other Hymenoptera OBP and CSP genes and on RNAseq data, when available. Lastly, the deduced amino acid sequences of *A. ervi* and *L. fabarum* OBP and CSP candidates were then used as query for another tBLASTn search against the genomes in an iterative process to identify any additional OBPs. Since both OBPs and CSPs are secreted proteins, the occurrence of a signal peptide was verified using SignalP [194, 195].

#### Odorant receptors (ORs)

ORs were annotated using available OR gene models from *Diachasma alloeum*, *Fopius arisanus*, and *Microplitis demolitor* retrieved from NCBIs protein database (retrieved May 2017). Preliminary OR genes models for *A. ervi* and *L. fabarum* were predicted with exonerate (v2.4.0), GeMoMa v1.4 [223], and combined with EVidence Modeler v1.1.1 [236]. These preliminary models were subsequently screened for the 7tm_6 protein domain (with PfamScan v1.5) and manually curated in WebApollo2.

In an iterative approach, we annotated the IRs using known IR sequences from *Apis melifera*, *Drosophila melanogaster*, *Microplitis demolitor and Nasonia vitripennis* as queries to identify IRs in the genomes of *A. ervi* and *L. fabarum*. The hymenopteran IR sequences served as input for the prediction of initial gene model with Exonerate [235] and GeMoMa [223]. Then, we inspected and edited homologous gene models from each tool in the Apollo genome browser to adjust for proper splice sites, start and stop codons in agreement with spliced RNA-Seq reads. After a first round of prediction, we repeated the whole process and provided the amino acid sequences of curated IR genes as queries for another round of predictions to identify any remaining paralogous IRs.

Multiple sequence alignments of the IRs were computed with hmmalign [237] using a custom IR HMM to guide the alignments [99]. Gene trees were generated with FastTree v2 [238] using the pseudocount option and further parameters for the reconstruction of an exhaustive, accurate tree (options: -pseudo -spr 4 -mlacc 2 -slownni). Resulting trees were visualized with iTOL v4 [239], well supported IR clusters and expansions were highlighted by color (branch support > 0.9).

### Sex determination genes

Ortholog searches were performed with tBLASTn [233] against the genomic scaffolds. Hits with an e-value smaller than 1e-20 were assessed, apart from *transformer* and *doublesex* where any hit was surveyed. Doublesex, Transformer-2 and Transformer peptide sequences of *Asobara tabida* (NCBI accessions MF074326-MF074334) were used as queries for the core sex determination genes. This braconid species is the closest relative whose sex determination mechanism has been examined (Geuverink et al., 2018). The putative *transformerB* sequence of *A. ervi* was blasted for verification against the transcriptome of *Aphidius colemani* [11] and a highly conserved fragment was detected (GBVE01021531). Peptide sequences of sex determination related genes to use as queries were taken from *Nasonia vitripennis*: Fruitless (NP_001157594), Sex-Lethal homolog (XP_016836645), pre-mRNA-splicing factor *CWC22* homolog (XP_001601117) and RNA-binding protein 1-like (XP_008202465). Hidden Markov models were not used as gene models because the ensuing peptide predictions did not contain all putative homologs (e.g. *transformerB* in *A. ervi*) due to fragmentation of the scaffolds containing the candidate genes.

### DNA methylation genes

The genomes were searched with tBLASTn [233] for the presence of potential DNA methyltransferase genes using peptide sequences from *Apis mellifera* and *N. vitripennis* as queries. These species differ in their copy number of *DNMT1*, with two copies (NP_001164522, XP_006562865) in the honeybee *A. mellifera* [240] and three copies (NP_001164521,XP_008217946, XP_001607336) in the wasp *N. vitripennis* [37]. To assess presence of *DNMT1* copies in other braconids, tBLASTn searches with DNMT1 queries were performed on whole genome shotgun assemblies of *M. demolitor*, *F. arisanus* and *C. vestalis* [32, 34, 36] *al.* 2019). *DNMT2*, currently characterized as EEF1AKMT1 (EEF1A Lysine Methyltransferase 1), has become redundant in the list of DNA methyltransferase genes as it methylates tRNA instead, but was surveyed here as a positive control (*N. vitripennis* NP_001123319, *A. mellifera* XP_003251471). *DNMT3* peptide sequences from *N. vitripennis* (XP_001599223) and from *A. mellifera* (NP_001177350) were used as queries for this gene. Low levels of methylation were confirmed by mapping the whole genome bisulfite sequencing data generated by Bewick et al. [108] back to the *A. ervi* genome assembly.

## Supplementary information

**Additional file 1. **Contamination filtering for *A. ervi*. Details of both blast-based and blobtools contamination filtering performed on the *A. ervi* genome.

**Additional file 2. **Contamination filtering for *L. fabarum*. Details of both blast-based and blobtools contamination filtering performed on the *L. fabarum* genome.

**Additional file 3. **Linkage groups in *L. fabarum.* Details of scaffold positions used to construct linkage groups for *L. fabarum*.

**Additional file 4. **Orphan genes in *A. ervi.* Fasta file containing the 2568 orphan genes identified in the *A. ervi* assembly.

**Additional file 5. **Orphan genes in *L. fabarum.* Fasta file containing the 968 orphan genes identified in the *L. fabarum* assembly.

**Additional file 6.** Details of CDS used to compare GC content across taxa. Genbank numbers and taxonomic information for genome (CDS) graphed in Supplementary Figure 9, detailing the GC content in predicted genes of other arthropods.

**Additional file 7. **Analysis of GC content and expression. Full details of analyses that drew from expression data in both taxa. This file details (a) GC content, carbon, and nitrogen for all predicted genes, (b) the most highly expressed genes in both taxa and (c) full analysis of differential expression between adult and larval *L. fabarum*, and corresponding comparison of GC content.

**Additional file 8. **Ortholog MAtrix (OMA) results. Summary of all Hierarchical Ortholog Groups (HOGs) predicted by OMA, including details of *LRR* genes.

**Additional file 9. **Venom gene annotations. Annotation details of venom genes in *L. fabarum* and *A. ervi.*

**Additional file 10. **Immune gene annotations. Details of immune gene annotation in *L. fabarum* and *A. ervi.*

**Additional file 11. **Expression of Osiris genes. Expression details of Osiris genes in *L. fabarum* and *A. ervi*, including comparison of expression between larval and adult *L. fabarum*.

**Additional file 12.** OXPHOS gene annotations. Details of annotated OXPHOS genes, including duplications in the assembly.

**Additional file 13.** Annotation of sex determination genes. Details of sex determination gene annotations.

**Additional file 14.** Supplementary Materials.

## Data Availability

Both genomes are available from the NCBI Genome database (PRJNA587428, *A. ervi*: SAMN13190903, *L. fabarum*: SAMN13190904). The bipaa webpage (https://bipaa.genouest.org) hosts the assemblies (https://bipaa.genouest.org/sp/aphidius_ervi/download/genome/v3.1/ and https://bipaa.genouest.org/sp/lysiphlebus_fabarum/download/genome/v1.0/), predicted genes, and annotations (https://bipaa.genouest.org/sp/aphidius_ervi/download/annotation/ and https://bipaa.genouest.org/sp/lysiphlebus_fabarum/download/annotation/v1.0/). Raw Illumina and PacBio sequence data used to construct genomes are available in NCBI SRA for both *A. ervi* (SAMN12878248) and *L. fabarum* (accessions SAMN10617865, SAMN10617866, SAMN10617867), and further detailed in Supplementary Tables 1 and 2. Venom protein data are available via ProteomeXchange (PXD015758; https://www.ebi.ac.uk/pride/archive/projects/PXD015758).

## Abbreviations

A, T, C, G, and U: Adenine, Thymine, Cytosine, Guanine, and Uracile, nucleotides
bp: Base Pair
BIPAA: BioInformatics Platform for Agroecosystem Arthropods (bipaa.genouest.org)
BLAST: Basic Local Alignment Search Tool
BUSCO: Benchmarking Universal Single-Copy Orthologs
CDS: Predicted Coding Sequence
CSD: Complementary Sex Determination
CHC: Cuticular Hydrocarbons
*DNMT*: DNA Methyltransferase genes
CSP: Chemosensory Protein
GO: Gene Ontology
HMM: Hidden Markov Model
HOG: Hierarchical Ortholog Group
IR: Ionotropic Receptor
*LRR*: Leucine Rich Repeat Proteins
Mbp: Mega Base Pairs, or 1000,000 bp
MP: Mate-pair sequence data
NCBI: National Center for Biotechnology Information
N50: A measure of genome completeness. The length of the scaffold containing the middle nucleotide
OBP: Odorant-binding Protein
OR: Odorant Receptor
OXPHOS: Oxidative Phosphorylation
PE: Paired-end sequence data
RSCU: Relative Synonymous Codon Usage
TE: Transposable Element
