# Supplementary material for: Functional insights from the GC-poor genomes of two aphid parasitoids, Aphidius ervi and Lysiphlebus fabarum
Source: BMC Genomics. 2020 May 29;21:376. doi: 10.1186/s12864-020-6764-0 (PMC7257214; doi:10.1186/s12864-020-6764-0)
Supplement: Supplementary file 14 — Additional file 14. Supplementary Materials. [file 12864_2020_6764_MOESM14_ESM.pdf]

Supplemental Materials

Functional insights from the GC-poor genomes of two aphid parasitoids, *Aphidius ervi* and *Lysiphlebus fabarum*

Alice B. Dennis[§1,2,3]*, Gabriel I. Ballesteros[§4,5,6], Stéphanie Robin[7,8], Lukas Schrader[9], Jens Bast[10,11], Jan Berghöfer[9], Leo Beukeboom[12], Maya Belghazi[13], Anthony Bretaudeau[7,8], Jan Buellesbach[9], Elizabeth Cash[14], Dominique Colinet[15], Zoé Dumas[10], Mohammed Errbii[9], Patrizia Falabella[16], Jean-Luc Gatti[15], Elzemiek Geuverink[12], Joshua D. Gibson[14,17], Corinne Hertäg[18,1], Stefanie Hartmann[3], Emmanuelle Jacquin-Joly[19], Mark Lammers[9], Blas I. Lavandero[6], Ina Lindenbaum[9], Lauriane Massardier-Galata[15], Camille Meslin[19], Nicolas Montagné[19], Nina Pak[14], Marylène Poirié[15], Rosanna Salvia[16], Chris R. Smith[20], Denis Tagu[7], Sophie Tares[15], Heiko Vogel[21], Tanja Schwander[10], Jean-Christophe Simon[7], Christian C. Figueroa[4,5], Christoph Vorburger[1,2], Fabrice Legeai[7,8], and Jürgen Gadau[9]

[1] Department of Aquatic Ecology, Eawag, 8600 Dübendorf, Switzerland
[2] Institute of Integrative Biology, ETH Zürich, 8092 Zürich, Switzerland
[3] Institute of Biochemistry and Biology, University of Potsdam, 14476 Potsdam, Germany
[4] Instituto de Ciencias Biológicas, Universidad de Talca, Talca, Chile
[5] Centre for Molecular and Functional Ecology in Agroecosystems, Universidad de Talca, Talca, Chile
[6] Laboratorio de Control Biológico, Instituto de Ciencias Biológicas, Universidad de Talca, Talca, Chile
[7] IGEPP, Agrocampus Ouest, INRAE, Université de Rennes, 35650 Le Rheu, France
[8] Université de Rennes 1, INRIA, CNRS, IRISA, 35000, Rennes, France
[9] Institute for Evolution and Biodiversity, Universität Münster, Münster, Germany
[10] Department of Ecology and Evolution, Université de Lausanne, 1015 Lausanne
[11] Institute of Zoology, Universität zu Köln, 50674 Köln
[12] Groningen Institute for Evolutionary Life Sciences, University of Groningen, Groningen, The Netherlands
[13] Aix-Marseille Univ, CNRS, INP, Inst Neurophysiopathol, PINT, PFNT, Marseille, France
[14] Department of Environmental Science, Policy, & Management, University of California, Berkeley, Berkeley, CA 94720, USA
[15] Université Côte d'Azur, INRAE, CNRS, ISA, Sophia Antipolis, France
[16] University of Basilicata, Department of Sciences, 85100 Potenza, Italy
[17] Department of Biology, Georgia Southern University, Statesboro, GA 30460, USA
[18] D-USYS, Department of Environmental Systems Sciences, ETH Zürich, Switzerland
[19] INRAE, Sorbonne Université, CNRS, IRD, UPEC, Université Paris Diderot, Institute of Ecology and Environmental Sciences of Paris, iEES-Paris, F-78000 Versailles, France
[20] Department of Biology, Earlham College, Richmond, IN USA 47374
[21] Max Planck Institute for Chemical Ecology, Department of Entomology, Jena, Germany

Contents

1. Supplementary results and figures
    a. Assemblies
    b. Gene predictions
    c. Contamination filtering
    d. Linkage groups
    e. Synteny between genomes
    f. Transposable Elements
    g. GC content analysis
    h. Gene family evolution
    i. Venom apparatus
    j. Cuticular hydrocarbon profiles
    k. Community annotation of individual gene families
2. Extended methods
3. References for Supplementary materials

Supplemental results and figures

Assemblies

The genome assemblies for *A. ervi* and *L. fabarum* were both constructed using hybrid approaches that incorporated high-coverage short read (Illumina) and long-read (Pac Bio) sequencing (Supplementary Tables 1, 2). This produced two high quality genome assemblies (N50 = 581kb and 216kb for *A. ervi* and *L. fabarum,* respectively). The assemblies have similar total lengths (139MB and 141MB), but different ranges of scaffold-sizes (Table 1, Supplementary Table 3). Both genomes can be accessed via the BioInformatics Platform for Agroecosystem Arthropods (BIPAA), which contains the full annotation report and can be searched via both keywords and BLAST (bipaa.genouest.org).

The difference in scaffold size-distribution between the two species is likely the result of the different assembly strategies for the two species: while *A. ervi* was assembled using the Illumina short-reads and then scaffolded with the long-read PacBio data, *L. fabarum* was assembled directly from PacBio sequences that had been error-corrected using the Illumina data. This resulted in fewer small scaffolds for the *L. fabarum* assembly, but both genomes have similar number of long scaffolds (>3,000bp, *A.ervi*: 1,503 and *L. fabarum*: 1,698; Table 1).

The assembly strategies differed between the two assemblies to optimize the available data. In *A. ervi*, short read MP libraries with multiple insert sizes permitted an assembly strategy that utilized the different insert sizes to produce the best assembly. In *L. fabarum*, MP libraries were only produced with ca. 5kb inserts and these insert sizes were not reliable. Therefore, the *L. fabarum* assembly used this data to error correct the long-reads (PacBio), and this produced the best assembly for this species. Both assembled genomes appear to be largely complete, with 97.6% (*A. ervi*) and 85.1% (*L. fabarum*) of the 1,658 core orthologous BUSCO genes for Insecta (insect_odb9) present in both species (Table 2); differences in the number of duplicated BUSCO genes is possibly due to the different assembly strategies, and future work with the *L. fabarum* genome should address possible duplications.

We used the program KAT (Mapleson, Garcia Accinelli et al. 2016) to conduct a kmer analysis on both assemblies (Supplemental Figure 1). In both species, a k-value of 27 was used, and the generated plot has a clear large homozygous peak. The *A. ervi* genome size was estimated at 142.83Mbp, with 92.85% completeness, with a mean overall kmer frequency of 51x, and estimated 0.53% heterozygous rate. The *L. fabarum* genome size was estimated at 99.26Mbp, with 98.03% completeness, with a mean k-mer frequency of 86x, and a heterozygous rate of 1.30%.

Supplementary Table 1: Summary of sequencing data Aphidius ervi. *Coverage based on filtered data and predicted genome size (140MB)(Ardila-Garcia et al. 2010). Hiseq v2000. N/A denotes that PacBio libraries are constructed without inserts.*

Library type	Host Strain	*Platform*	*Filtered reads*	*Read Length (bp)*	*Coverage*	*Insert size*	*SRA Accession*
Paired End	*Sitobion avenae*	Illumina HiSeq	60,828,295	100	86x	350bp	SRR10311786
Mate Paired 3kb	*Sitobion avenae*	Illumina HiSeq	14,959,974	100	21x	3kb	SRR9326705
Mate Paired 5kb	*Sitobion avenae*	Illumina HiSeq	15,122,455	100	22x	5kb	SRR9326706
Mate Paired 8kb	*Sitobion avenae*	Illumina HiSeq	15,138,323	100	22x	8kb	SRR9326707
Long Read Fragments	*Acyrthosiphon pisum*	PacBio RSII	622,140	6074 (mean)	27x	N/A	SRR10208676

Supplementary Table 2: Summary of sequencing data Lysiphlebus fabarum. *Coverage based on filtered data and predicted genome size (128MB) (Belshaw & Quicke 2003). MiSeq reagents v3. N/A denotes that PacBio libraries are constructed without inserts.*

Library Type	*Platform*	*Filtered reads*	*Read Length (bp)*	*Coverage*	*Insert Size (bp)*	*SRA Accession*
Paired End	Illumina MiSeq	20,313,003	300	32x	169 +/-10	SAMN10617865
Mate Pair 5kb	Illumina MiSeq	5,361,287	300	25x	3782 +/- 404	SAMN10617866
Long Read Fragments	PacBio RS	617,092	3658	42x	N/A	SAMN10617867

Supplementary Table 3: Detailed report of genome assemblies and gene predictions.

		A. ervi	*L. fabarum*
Overview	Total length	138,845,131	140,705,580
	n contigs	12,948	na
	n scaffolds	5,743	1,698
	Longest scaffold	3,671,467	2,183,677
	n scaffolds ≥ 1000 bp	5,742	1,698
	n scaffolds ≥ 3000 bp	1,493	1,698
	N50	581,355	216,143
	n "N"'s	207,520	0
	GC %	25.8%	23.8%
Exons	n Exons	95,299	74,701
	Longest exon	13,754	11,848
	Mean exon length	311	317
Introns	n Introns	74,971	59,498
	Longest intron	27,991	19,865
	Mean intron length	395	383
Genes	n Genes	20,210	15,170
	Longest gene	96,195	65,965
	Mean gene length	2,920	3,052
	% genome covered by genes	42.5%	32.9%
CDS	n CDS	20,328	15,203
	Longest CDS	43,731	27,132
	Mean CDS length	1,217	1,381
	% genome covered by CDS	17.8%	14.9%
	GC% in CDS	31.0%	29.8%

Supplementary Table 4: Summary of BUSCO statistics for the two species based on matches to the insecta_odb9 database of 1,658 total BUSCO groups

BUSCO statistics		
Whole genome assembly (nucleotide)		
	A. ervi	***L. fabarum***
Complete, single-copy	1,571 (94.8%)	1,265 (76.3%)
Complete, duplicated	46 (2.8%)	146 (8.8%)
Total Complete	*1,617 (97.3%)*	*1,411 (85.1%)*
Fragmented	13 (0.8%)	12 (0.7%)
Missing	28 (1.6%)	235 (14.2%)
Predicted genes (CDS, protein level)		
Complete, single-copy	1,504 (90.7%)	1,404 (84.7%)
Complete, duplicated	50 (3.0%)	185 (11.2%)
Total Complete	*1,554 (93.7%)*	*1,589 (95.9%)*
Fragmented	32 (1.9%)	14 (0.8%)
Missing	72 (4.4%)	55 (3.3%)

Supplementary Figure 1: Read k-mer frequency vs assembly copy number for the A. ervi and L. fabarum genome assemblies, generated by KAT. Reads in black are absent from the assemblies, red occurs once, purple twice, and green three times. The largest peaks represent homozygous content, mapping only once, at x=50 for A. ervi *and x=86 for* L. fabarum*.*

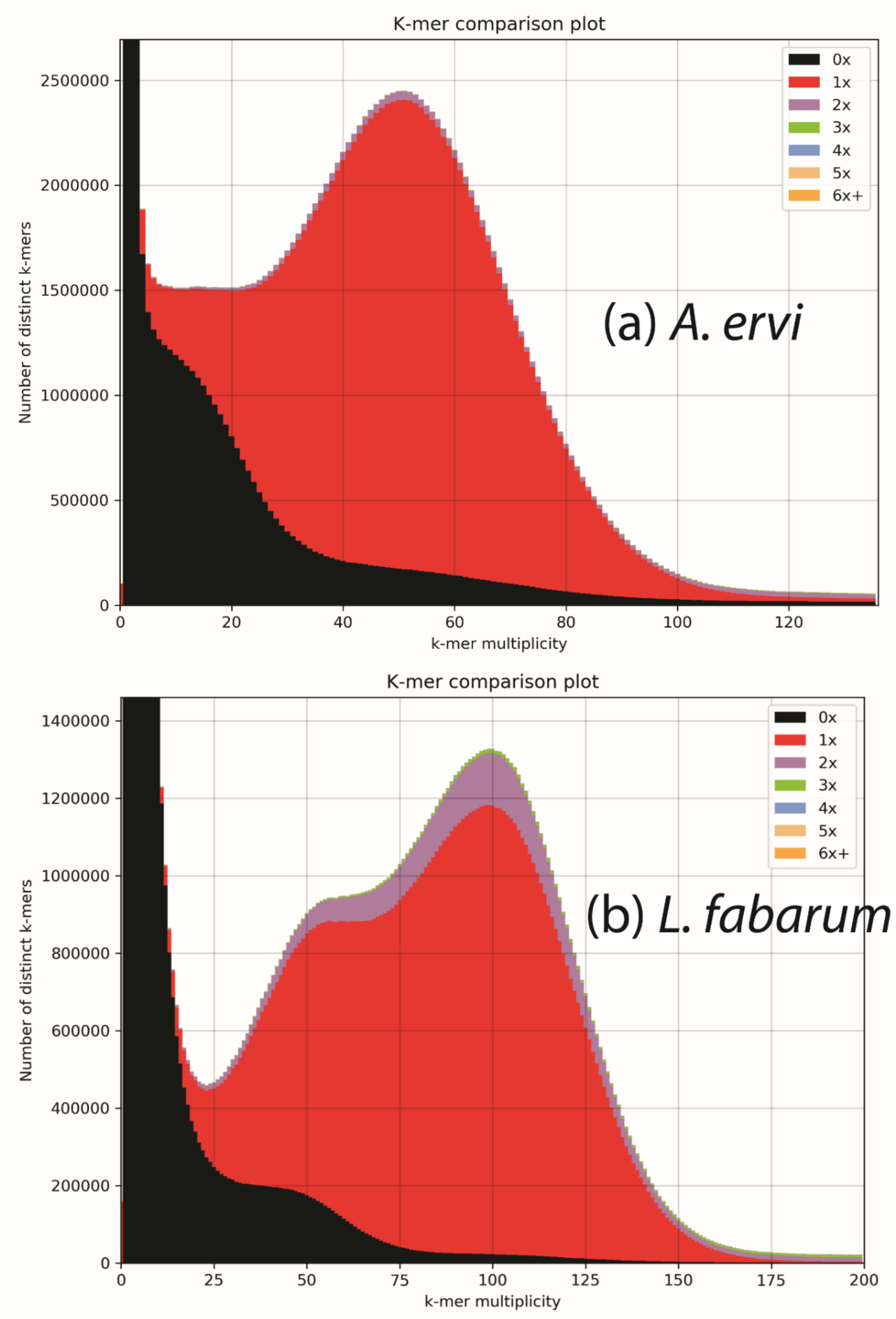

Gene predictions

The Maker2 annotation pipeline predicted different numbers of coding genes (CDS) for the two genomes: in *A. ervi* there were 20,344 predicted genes comprising 27.8Mbp, while in *L. fabarum* there were 15,203 genes across 21.9 Mbp. These numbers are on par with those predicted in other hymenopteran genomes (Table 3) and comparisons with these taxa suggest that the lower number of predicted genes in *L. fabarum* are more likely due to its loss than a gain in genes by *A. ervi*. In parallel with the difference in predicted genes, the predicted number of introns and exons was also higher in *A. ervi* (Table 1). In both species, there was high transcriptomic support for the predicted genes, although this was higher for *L. fabarum* (88.3% of genes) than *A. ervi* (77.8%). This difference likely reflects the available transcriptomic data rather than differences in the success of gene predictions. The resulting peptides were further functionally annotated against the NCBI *nr* database (NCBI), matched to gene ontology (GO) terms, and predictions for known protein motifs, signal peptides, and transmembrane domains (Supplemental Table 5). We also re-ran BUSCO to measure the presence of known, core, orthologous genes within the predicted proteins. At the protein level, BUSCO matches from the predicted genes were increased for *L. fabarum* (*A. ervi*: 93.7%, *L. fabarum*: 95.9%) over the nucleotide-level search of the whole genome (*A. ervi*: 97.6%, *L. fabarum*: 85.1%, Supplementary Table 4).

Supplementary Table 5: Summary of functional annotation of peptides.

Proteins with:	*A. ervi*	*L. fabarum*
BLAST match to *nr*	13, 582	12,832
GO annotations	5,870	5,965
Interproscan domains	14,901	12,792
Signal peptides	1,731	1,474
Transmembrane domains	3,957	3,254

Contamination filtering

We checked the assembled genomes for contamination using two approaches: (1) the program blobtools was used to identify outlier scaffolds based on GC content, sequencing coverage, and top BLAST score, and (2) we used a BLAST-based screen to identify predicted genes that could be possible contamination from host aphids.

Visualization with blobtools showed that there were very few outlier scaffolds. Further examination of *A. ervi* scaffolds that did not match to Arthropods identified 31 scaffolds that matched to bacteria; these were removed from the assembly. The most obvious outlier in *L. fabarum* was a single scaffold with high coverage (tig00001511, 10,205bp, 11.1% GC). A BLASTn search against the NCBI *nt* database matched this to the mitochondrial genome of *Aphidius gifuensis*, and we conclude that this is the mitochondrial genome for *L. fabarum*.

The majority of predicted genes matched best to the other wasp species (Supplementary Table 6). Of those that matched to aphid, the majority of these were single predicted genes that individually matched to aphids, while the rest of the predicted genes on the scaffold (>10, often hundred) matched to wasp (Additional Files 1 and 2) . For remaining ambiguous cases (21 *A. ervi* genes and 8 *L. fabarum*), the entire scaffolds were manually inspected. In every case, the *L. fabarum* scaffolds containing potential contamination were retained in the assembly; these appear to be repetitive regions and highly conserved genes. In *A. ervi*, five scaffolds appeared to come from other taxa. Four of these matched bacteria, but one of these was already identified by blobtools. The fifth scaffold appears to come from aphid. In total, this search resulted in four additional scaffolds that were removed from the assembly.

*Supplementary Table 6: Summary of filtering to remove potential containation from the genome assemblies. *Note, one scaffold was identified by both contamination screens, so a total of 34 scaffolds were removed from the assembly.*

L. fabarum		
Results of BLAST-based filter to separate wasp from aphid contamination of 15,203 genes		
	A. ervi	11,924
	Multi	2,572
	No hit	655
	Aphid	52
	Of these in clear wasp scaffolds	45
	Of these likely conserved or shared repetitive regions	8
	Of these likely contamination	0
Results of blobtools screening of 1,698 scaffolds		
	Scaffolds blasting to Arthropod or nothing	1682
	All other matches (manually examined)	16
	Of these, removed	0
A. ervi		
Results of BLAST-based filter to separate wasp from aphid contamination of 20,344 genes		
	L. fabarum	17,607
	Multi	380
	No hit	2,264
	Aphid	93
	Of these in clear wasp scaffolds	71
	Of these likely conserved or repetitive shared regions	15
	Of these likely contamination, removed	**5***
Results of blobtools screening of 5,778 scaffolds		
	Scaffolds blasting to Arthropod or nothing	5,725
	All other matches (manually examined)	53
	Of these likely contamination, removed	**31***

Supplementary Figure 2: Plot from blobtools for A. ervi*, depicting read-coverage from the mapped PE data against GC content of all scaffolds. Circle diameters are proportional to scaffold length, colors are based on top BLAST hit for the scaffold. All non-Arthropod matches were manually inspected. The marked outliers with low coverage and high GC content were largely bacterial scaffolds and were removed from the assembly.*

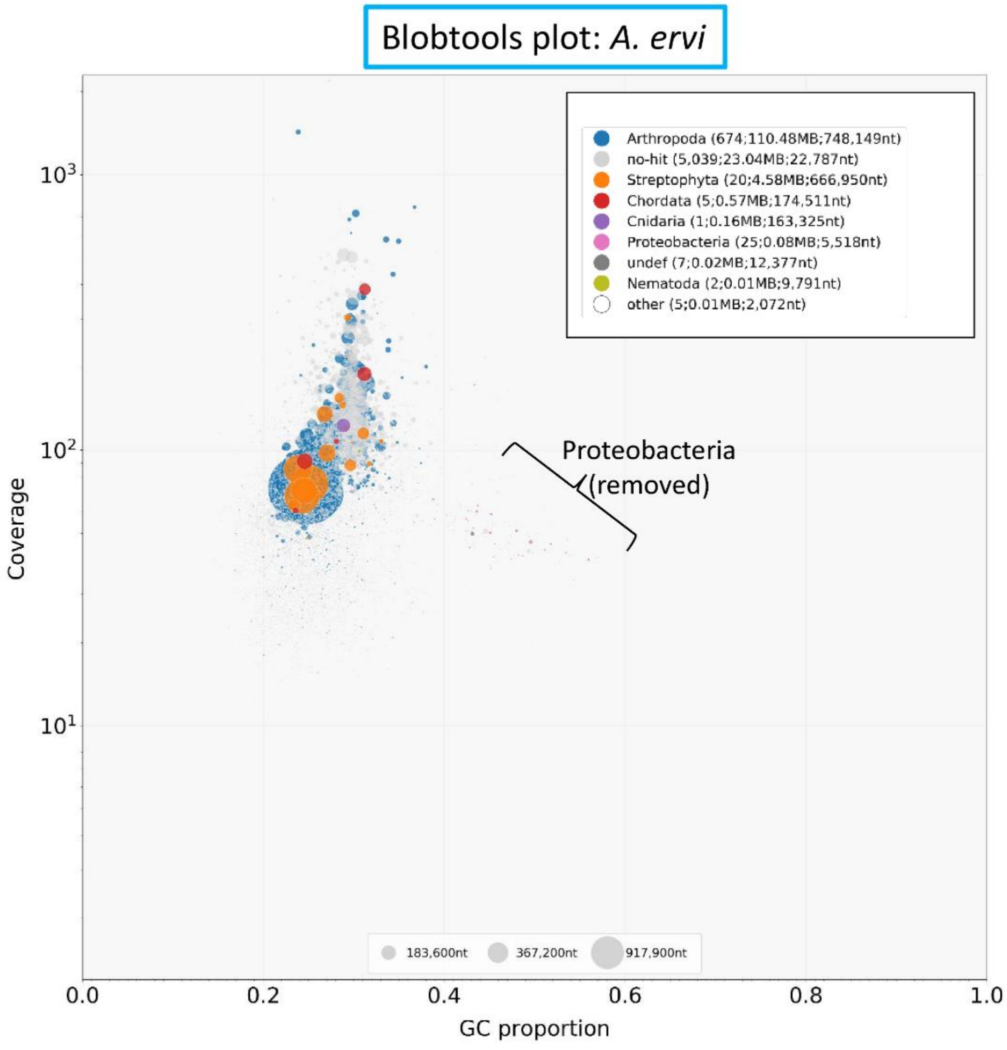

Supplementary Figure 3: Plots from blobtools for L. fabarum*, depicting read-coverage from the mapped PE data against GC content of all scaffolds. Circle diameters are proportional to scaffold length, colors are based on top BLAST hit for the scaffold. All non-Arthropod matches were manually inspected.*

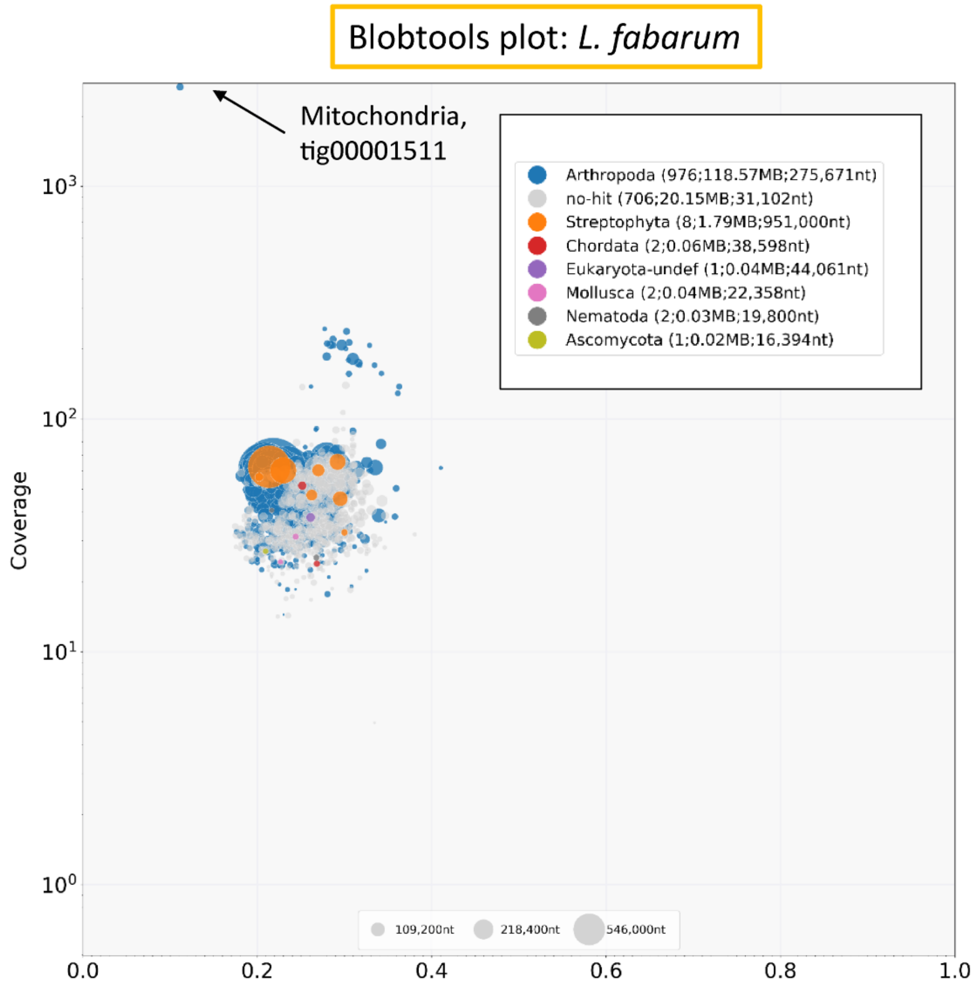

Linkage groups in *L. fabarum*

We constructed linkage groups for the *L. fabarum* scaffolds using phased SNPs from the haploid (male) son of a single female wasp. This placed the 297 largest scaffolds (>50% of the nucleotides, Supplementary table 7, Supplementary Figure 4), into the expected six chromosomes (Belshaw and Quicke 2003).

Supplementary Table 7: Summary statistics of linkage groups built for the L. fabarum *genome. Linkage groups based on 1,319 biallelic SNPs.*

Linkage groups		*n*	bp
	Linkage groups	6	3.7 – 17 Mbp
	Incorporated scaffolds	297	75,424,286
	Unincorporated	1,401	65,310,394

Supplementary Figure 4: Linkage mapping of L. fabarum *scaffolds (colored in black and white). Based on 1,319 biallelic SNPS from 90 haploid males, all from a single mother.*

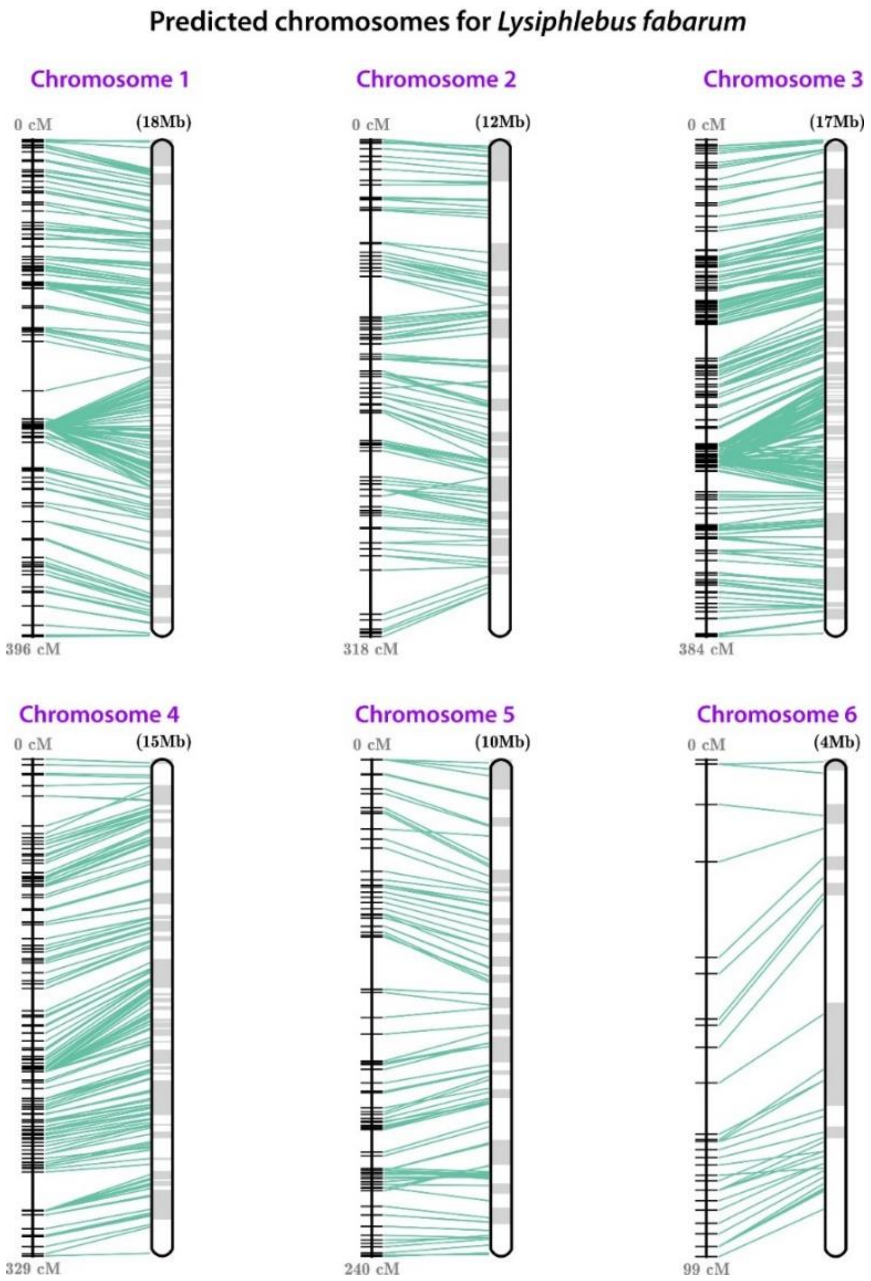

Synteny between the two genomes

With this largely contiguous assembly (the six chromosomes plus unincorporated pieces: 1,407 scaffolds altogether), we can show synteny between the two genomes. We identified syntenic regions between the two genomes by mapping with NUCmer, which is part of the MUMer package (Kurtz, Phillippy et al. 2004). NUCmer identified 67,557 matches among all scaffolds in the two genomes (Supplementary Figure 5). To further examine large synteny blocks within this, we created a plot using only the six predicted *L. fabarum* chromosomes and the *A. ervi* scaffolds longer than 1MBp. The matches between these were filtered to retain only instances with at least three consecutive matches of >250bp. This produced 358 connections (each of which represent multiple NUCmer matches), and linked 28 *A. ervi* scaffolds to the six *L. fabarum* chromosomes (Supplemental Figure 6).

Supplementary Figure 5: Whole-genome alignment generated by NUCmer. Forward matches are shown in red and reverse matches in blue.

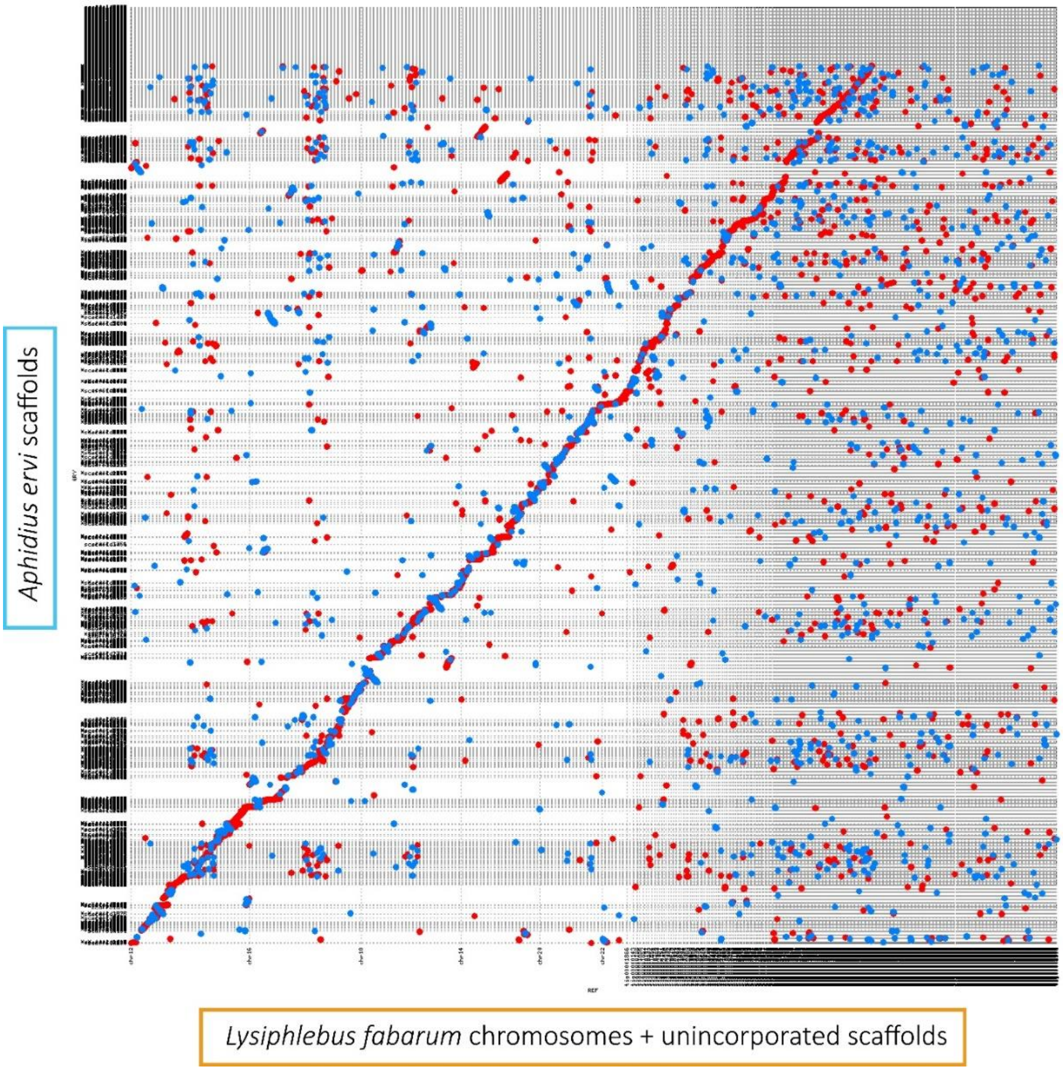

Supplementary Figure 6: Syntenic region between the six predicted chromosomes in the L. fabarum *genome and scaffolds in the* A. ervi *assembly. This alignment only included* A. ervi *scaffolds > 1Mbp and instances of at least three consecutive matches with a minimum of 250bp.*

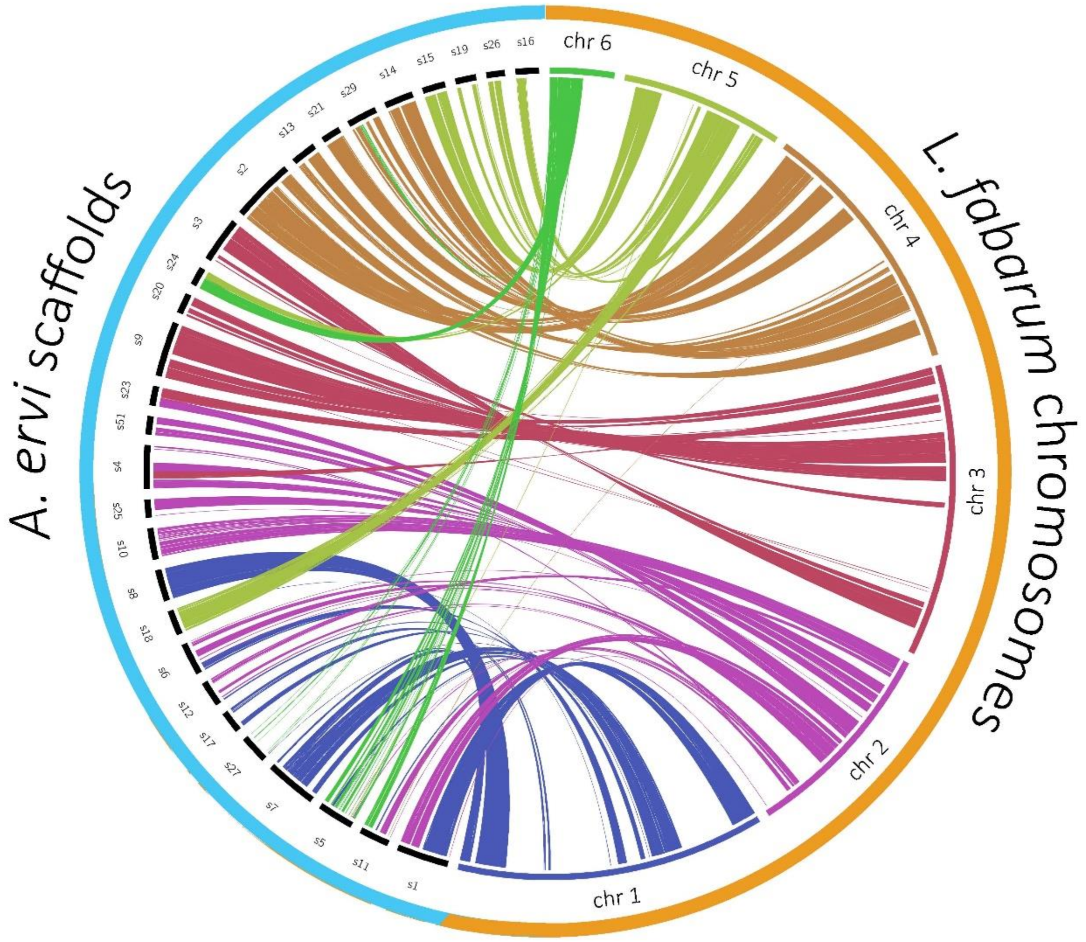

Transposable Elements (TEs)

Predictive annotation of Transposable Elements (TEs) identified a similar overall number of putative TE elements in the two genomes (*A. ervi*: 67,695 and *L. fabarum*: 60,306, Supplementary table 8). Despite this similarity, the overall genomic coverage by TEs is larger in *L. fabarum* (41%, 58 Mbp) than in A. *ervi* (22%, 31 Mbp). The spread of reported TE coverage in arthropods is quite large, even among *Drosophila* species (ca. 2.7% - 25%, Drosophila 12 Genomes, Clark et al. 2007). Within parasitoids, reported TE content also varies, and relatively low coverage in the parasitoid *Macrocentrus cingulum* (ca. 18%, Yin, Li et al. 2018) in comparison to *Nasonia vitripennis* was attributed to the differences in their genome sizes (127.9Mbp and 295.7Mbp, respectively, Table 3). However, the variation we observe here suggests that differences in predicted TE content may be evolutionary quite labile, even within closely related species with the same genome size.

There is also a difference in the overall types of elements composing the TE's in the two genomes, and in their size distributions (Supplemental Figures 7 and 8, Supplementary table 8). This difference could contribute to the lower GC content in *A. ervi* relative to *L. fabarum*. However, this should also be examined in light of the different assembly strategies in these two genomes. Specifically, that the long-read based assembly of the *L. fabarum* genome may have better reconstructed the large elements than the short-read based assembly in *A. ervi*. However, it does not appear that the different assembly strategies are the sole reason for differences in predicted TE's. Estimates of repeat content based directly on unassembled short read data also confirm that the genome of *L. fabarum* has a higher repeat content compared to *A. ervi*. These results from DNApipTE suggest that most of these differences are caused by low-complexity sequence and simple repeats (Supplemental table 9).

Supplementary Table 8: Summary of de novo *TE annotations. nb= number.*

	A. ervi	*L. fabarum*
number of sequences	297	1101
number of matched sequences	297	1100
cumulative coverage	31,015,099 bp	58,003,749 bp
coverage percentage	22.32%	41.22%
total nb of TE fragments	67,695	60,306
total nb full-length fragments	3,740 (5.52%)	5,513 (9.14%)
total nb of TE copies	64,653	49,579
total nb full-length copies	3,869 (5.98%)	5,698 (11.49%)
families with full-length fragments	297 (100.00%)	1,096 (99.55%)
with only one full-length fragment	23	307
with only two full-length fragments	33	377
with only three full-length fragments	27	129
with more than three full-length fragments	214	283
families with full-length copies	297 (100.00%)	1,094 (99.36%)
with only one full-length copy	22	282
with only two full-length copies	27	379
with only three full-length copies	32	134
with more than three full-length copies	216	299
mean of median identity of all families	84.47 +- 8.52	92.71 +- 5.54
mean of median length percentage of all families	49.18 +- 28.57	39.99 +- 34.78

Supplementary Table 9: Summary of direct estimates of relative repeat content from unassembled short reads from DNApipTE

Type	*A. ervi*	*L. fabarum*
**LTR**	1.7%	2.6%
**LINE**	0.9%	1.5%
**SINE**	0.0%	0.0%
**DNA**	4.5%	4.7%
**MITE**	0.0%	0.0%
**Helitron**	0.7%	0.8%
**rRNA**	0.2%	0.8%
**Low_Complexity**	2.7%	6.4%
**Satellite**	0.1%	0.1%
**Tandem_repeats**	0.0%	0.0%
**Simple_repeat**	9.5%	25.9%
**others**	0.2%	0.7%
**na**	8.8%	5.8%
**Others**	0.0%	0.0%

Supplementary Figure 7: Distribution of TE classes in A. ervi

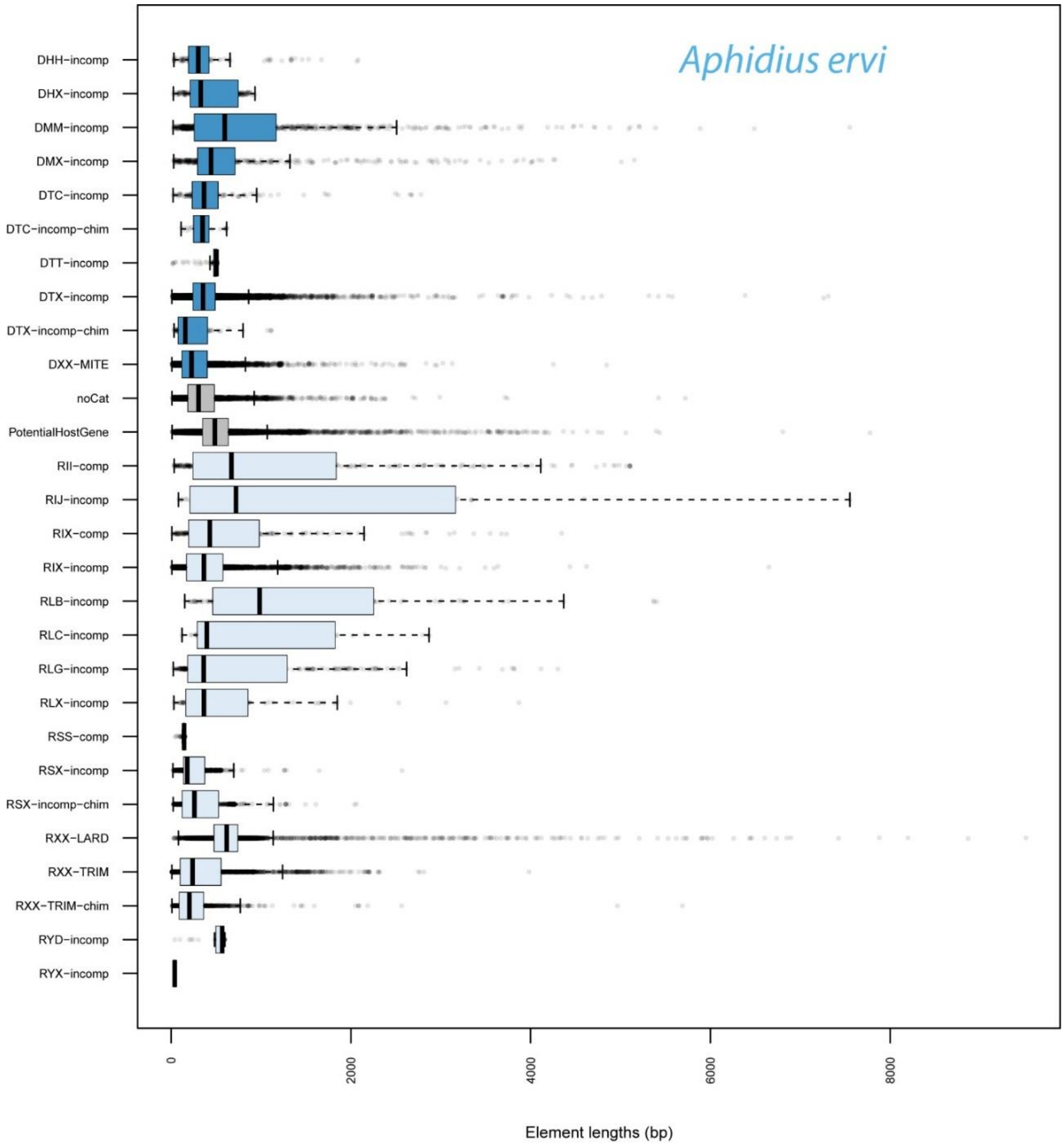

Supplementary Figure 8: Distribution of TE classes in L. fabarum

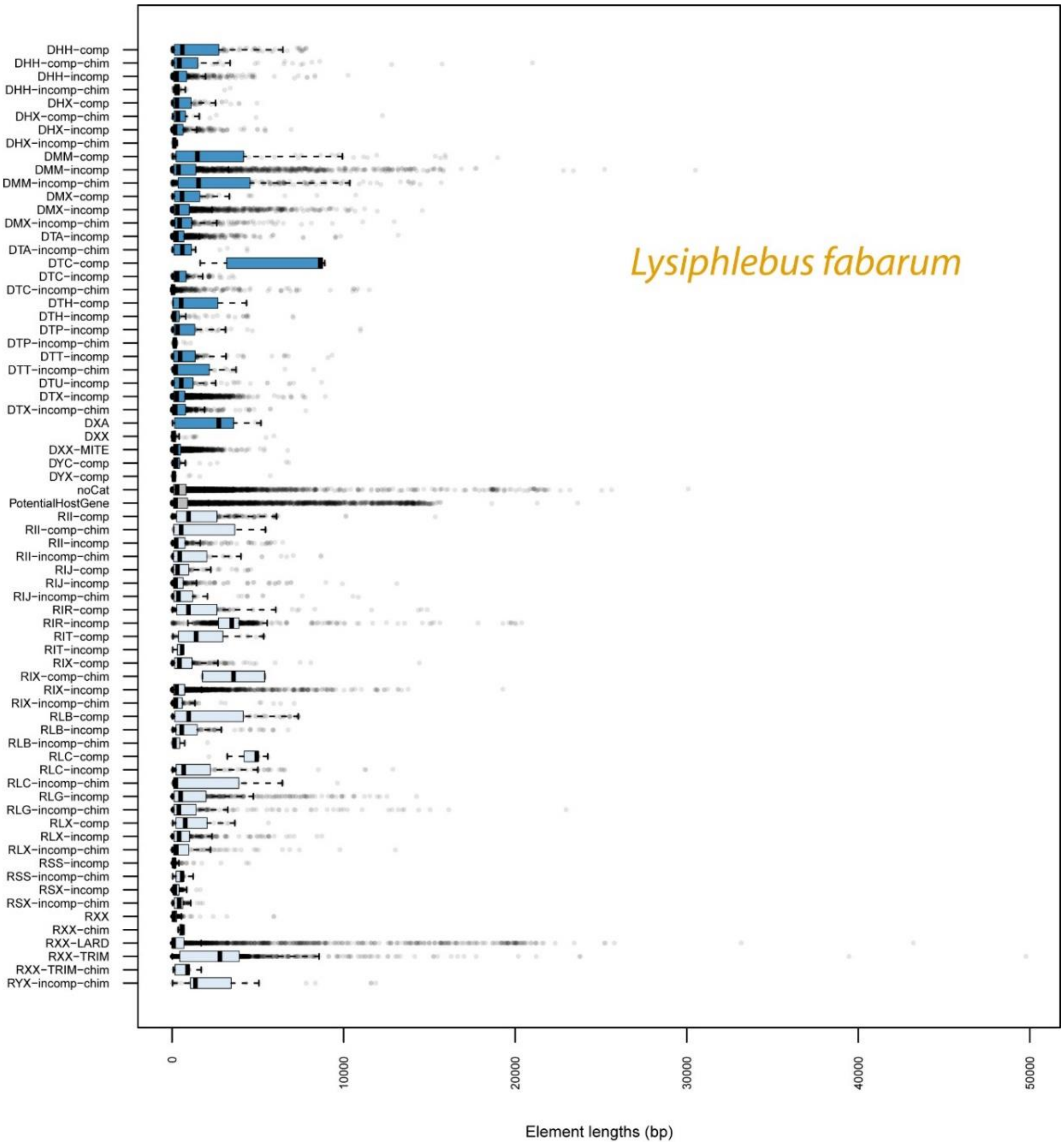

GC Content – in relation to other taxa

We compared GC content within the predicted genes (CDS) of all insect taxa for which this information was available in NCBI (October 2018). These taxa are listed in Supplemental Data File 6.

Supplementary Figure 9: Boxplot of GC content in all predicted genes. Boxes represent the upper and lower quartiles of the data and their midline depicts the median. Vertical whiskers depict 1.5 the interquartile range. Outliers are shown as black dots. Taxa represented are all Insecta available in the RefSeq RNA database downloaded from NCBI in October 2018, plus the two genomes presented in this paper (far right, colored).

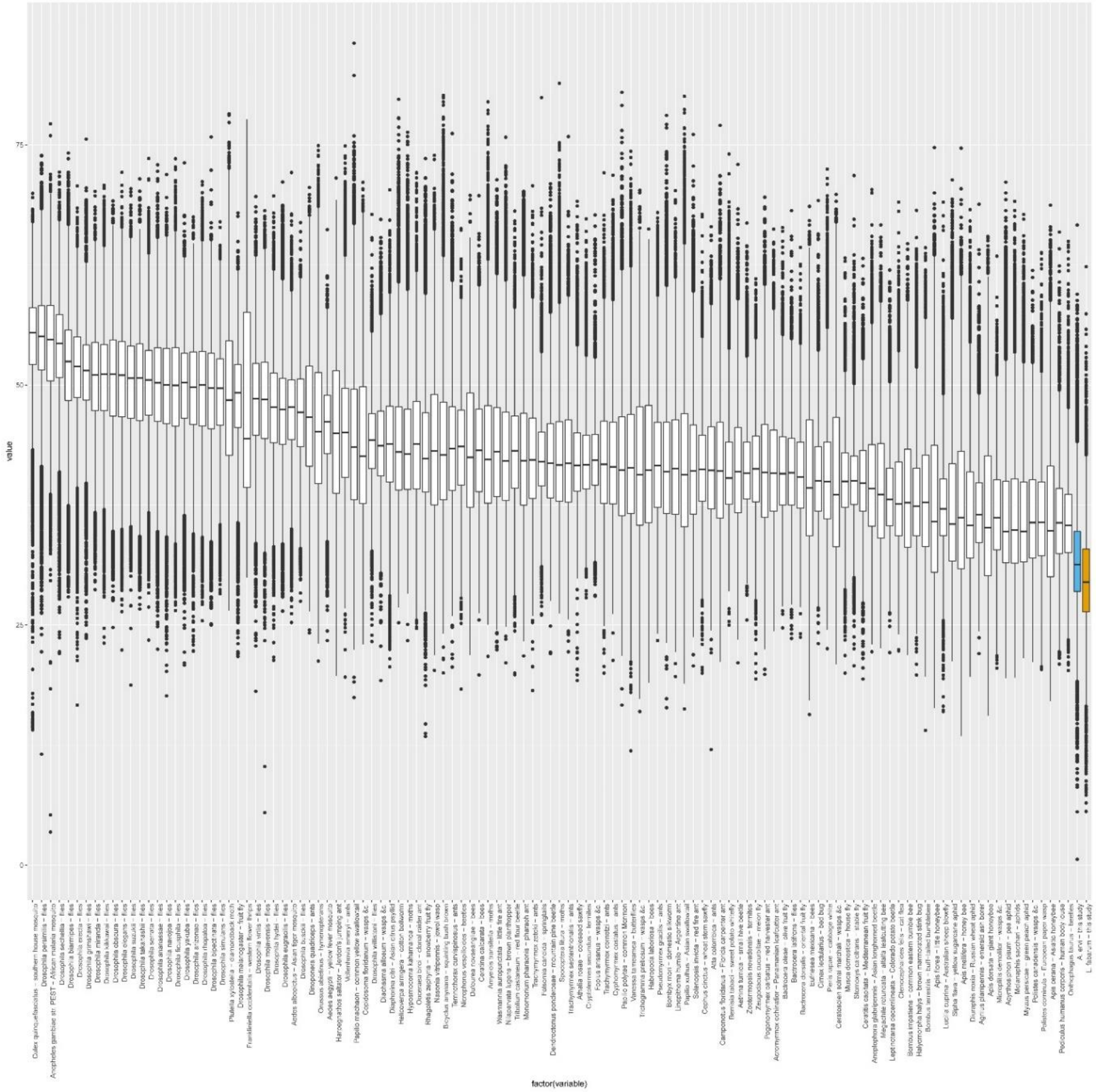

GC content – in relation to the environment

To examine potential impacts of nutrient availability in the environment (namely nitrogen and carbon), we calculated several aspects of the chemical composition of expressed genes in both genomes. We compared the most highly (10%) and lowly (10%) expressed genes in both genomes, based on the idea that highly expressed genes would have higher material costs (Bragg and Wagner 2009).

We measured:

1) GC content of the expressed genes

The GC content of the most highly expressed genes was significantly higher in both species (Supplementary figure 13, panels A and B). This pattern has been reported broadly in other taxa (Chaney and Clark 2015), and does not suggest that overall expression level is selecting for lower GC content in these two taxa.

2) N-atoms per gene

There is evidence that ecologically limiting nitrogen can select for lower nitrogen content in expressed genes (Acquisti, Elser et al. 2009). We calculated nitrogen content in the expressed genes by calculating the total N-atoms in the expressed genes. This calculation (i.e. the RNA from our gene predictions) was performed using the weighting used in Acquisti et al (2009), and was conducted as follows: n_A= 5, n_T = 2, n_C= 3, n_G= 5. These comparisons showed that the most highly expressed genes had significantly higher N-content (Supplemental Figure 13, panels C and D). There is a strong relationship between nitrogen content and GC content, because higher GC genes implicitly have higher N-content. Thus, we believe that this pattern is the result of the GC- nitrogen relationship, rather than evidence of selection based on nitrogen limitation.

3) Carbon: nitrogen ratios

As another comparison of nitrogen content, we scaled the nitrogen content by the carbon content. This was intended to correct for overall size of the amino acid and aid visualization (Elser, Fagan et al. 2006). As with the total N-atoms per gene, we see no evidence for selection on lower-nitrogen content. Instead, the most highly expressed genes have the a lower ratio of carbon:nitrogen (Supplemental Figure 13, panels E and F), meaning that they have higher nitrogen content. Therefore, this does not support the idea that more highly expressed genes are under selection to conserve nitrogen.

<u>A note about our assembly and GC content:</u>

We do not believe the low GC content in these two genome assemblies is a byproduct of an incomplete assembly or some other bias (as has been previously observed in other hymenoptera, see: Elsik, Worley et al. 2014) for several reasons. Principal among these: (1) we have predicted a high presence of core BUSCO genes, (2) the numbers of genes are similar to those predicted in hymenoptera with higher GC content (Table 3), (3) we have incorporated long-read technology (PacBio), which should not have the same GC biases as Illumina-reads (Rhoads and Au 2015; Gan, Linton et al. 2019), and (4) separate transcriptomic studies in both species have produced transcripts with similar GC content (Ballesteros, Gadau et al. 2017; Dennis, Patel et al. 2017; Dennis, Käch et al. in revision)

GC content- in relation to other taxa

To examine the possibility that codon usage in this system is related to their environment and/or host, we examined RSCU, nitrogen content, and nitrogen:carbon ratios in *A. ervi* and *L. fabarum*, in comparison to other taxa from this system: namely four different aphid hosts and their endosymbionts, as well as the model parasitoid *N. vitripennis*. All calculations used only the predicted coding genes. Calculations of RSCU, nitrogen (N) and carbon (C) content, and PCA were performed as detailed above for the two parasitoid genomes.

Supplementary Table 10: Summary of taxa used in comparative analysis of codon usage, GC-content, and C:N ratios

Group	*Species*	*Source*
Host aphid	*Aphis glycines*	Wenger *et al.* (2017)
	Acyrthosiphon pisum	Thorpe *et al.* (2018)
	Myzus persicae – clone G006	Legeai *et al.* (2010)
	Myzus persicae – clone O	Legeai *et al.* (2010)
1° endosymbiont	*Buchnera aphidicola* from *A. glycines*	Cassone *et al.* (2015)
	B. aphidicola from *A. pisum*	Shigenobu *et al.* (2000)
	B. aphidicola from *M. persicae* strain W106	Jiang *et al.* (2013)
2° endosymbiont	*Hamiltonella defensa* from *A. pisum*	Degnan *et al.* (2009)
Parasitoid wasp	*Nasonia vitripennis v2*	Rago *et al.* (2016)
	Lysiphlebus fabarum	***this study***
	Aphidius ervi	***this study***

Supplementary Figure 10: Relative Synonymous Codon Usage (RSCU) is not similar to other taxa in this system, nor to the parasitoid wasp Nasonia vitripennis*. Note: two of the date points for* Buchnera *overlap completely.*

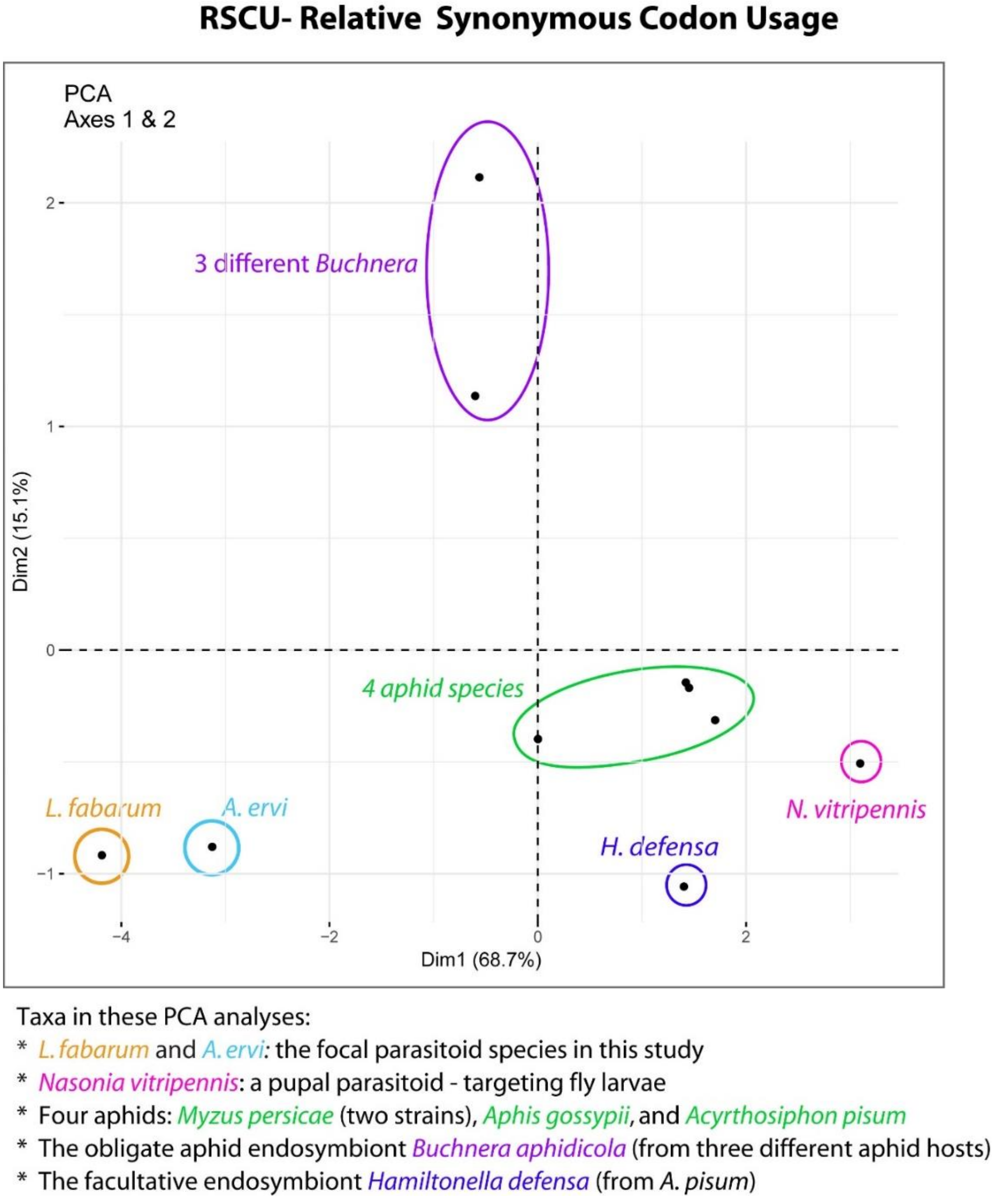

Supplementary Figure 11: Ratio of carbon: nitrogen in the CDS of all predicted genes, shows little relationship among taxa in this system, nor to the parasitoid wasp Nasonia vitripennis*.*

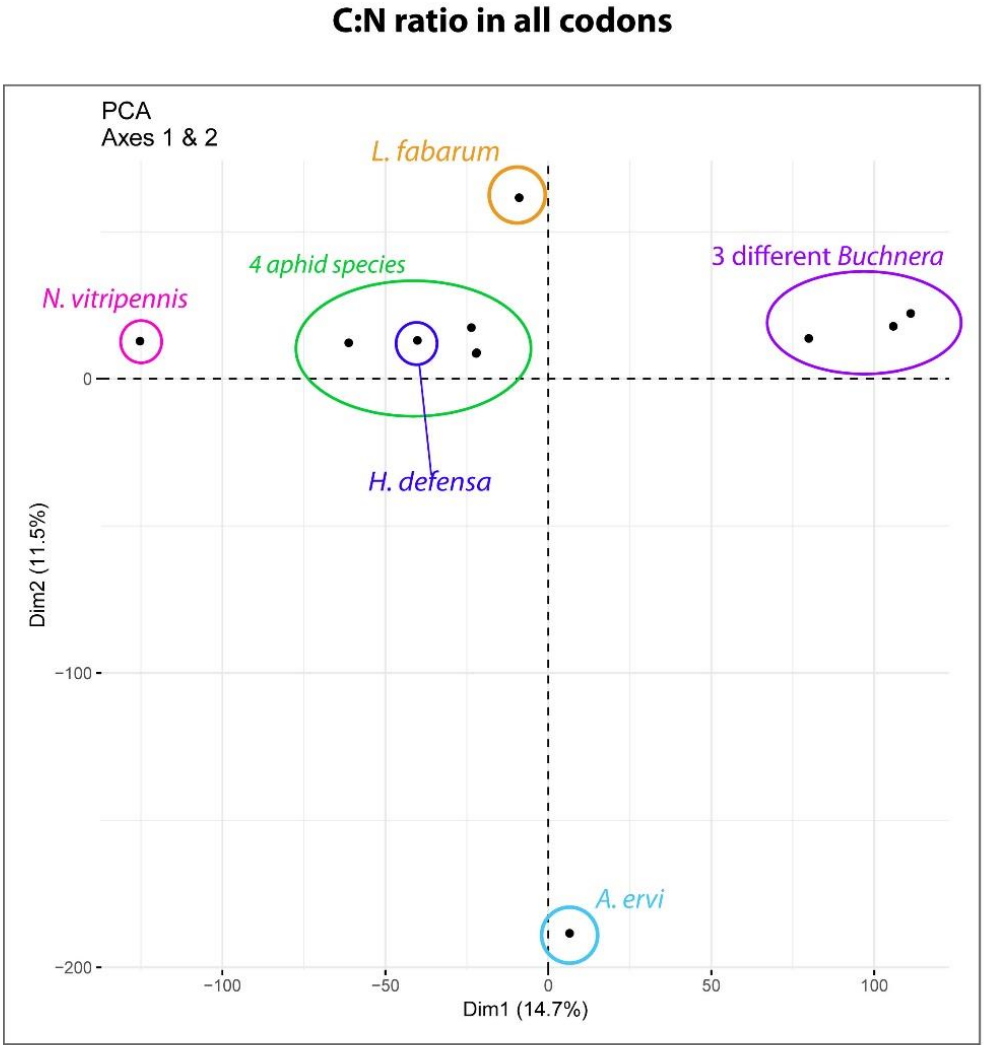

Taxa in these PCA analyses:
* *L. fabarum* and *A. ervi*: the focal parasitoid species in this study
* *Nasonia vitripennis*: a pupal parasitoid - targeting fly larvae
* Four aphids: *Myzus persicae* (two strains), *Aphis gossypii*, and *Acyrthosiphon pisum*
* The obligate aphid endosymbiont *Buchnera aphidicola* (from three different aphid hosts)
* The facultative endosymbiont *Hamiltonella defensa* (from *A. pisum*)

Supplementary Figure 12: Count of total nitrogen, scaled to the number of amino acids (AA) in each predicted gene, for available genomes in this system, and the parasitoid wasp Nasonia vitripennis.

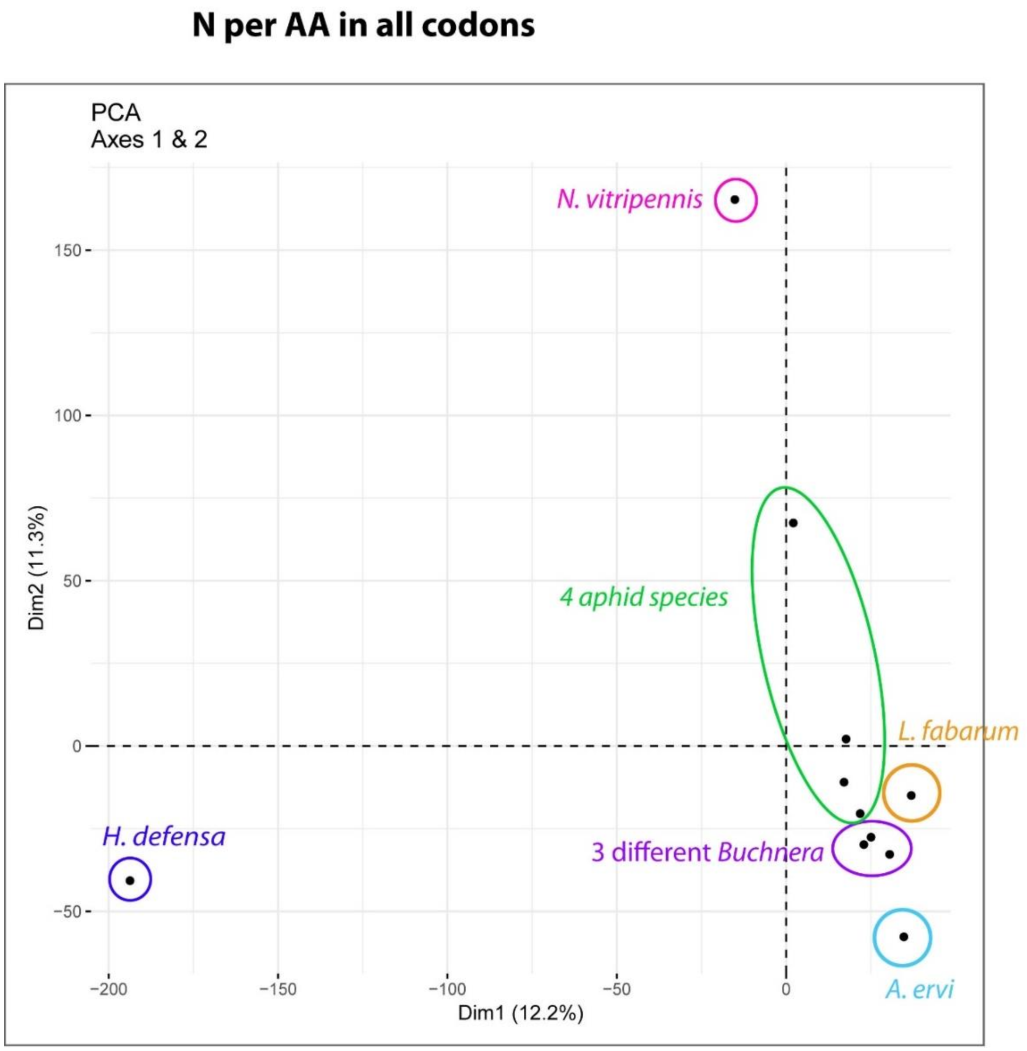

Taxa in these PCA analyses:
* *L. fabarum* and *A. ervi:* the focal parasitoid species in this study
* *Nasonia vitripennis*: a pupal parasitoid - targeting fly larvae
* Four aphids: *Myzus persicae* (two strains), *Aphis gossypii*, and *Acyrthosiphon pisum*
* The obligate aphid endosymbiont *Buchnera aphidicola* (from three different aphid hosts)
* The facultative endosymbiont *Hamiltonella defensa* (from *A. pisum*)

Supplementary Figure 13: Content of top 10% of expressed genes in L. fabarum *(n=1,374) and* A. ervi *(n=2,035)*

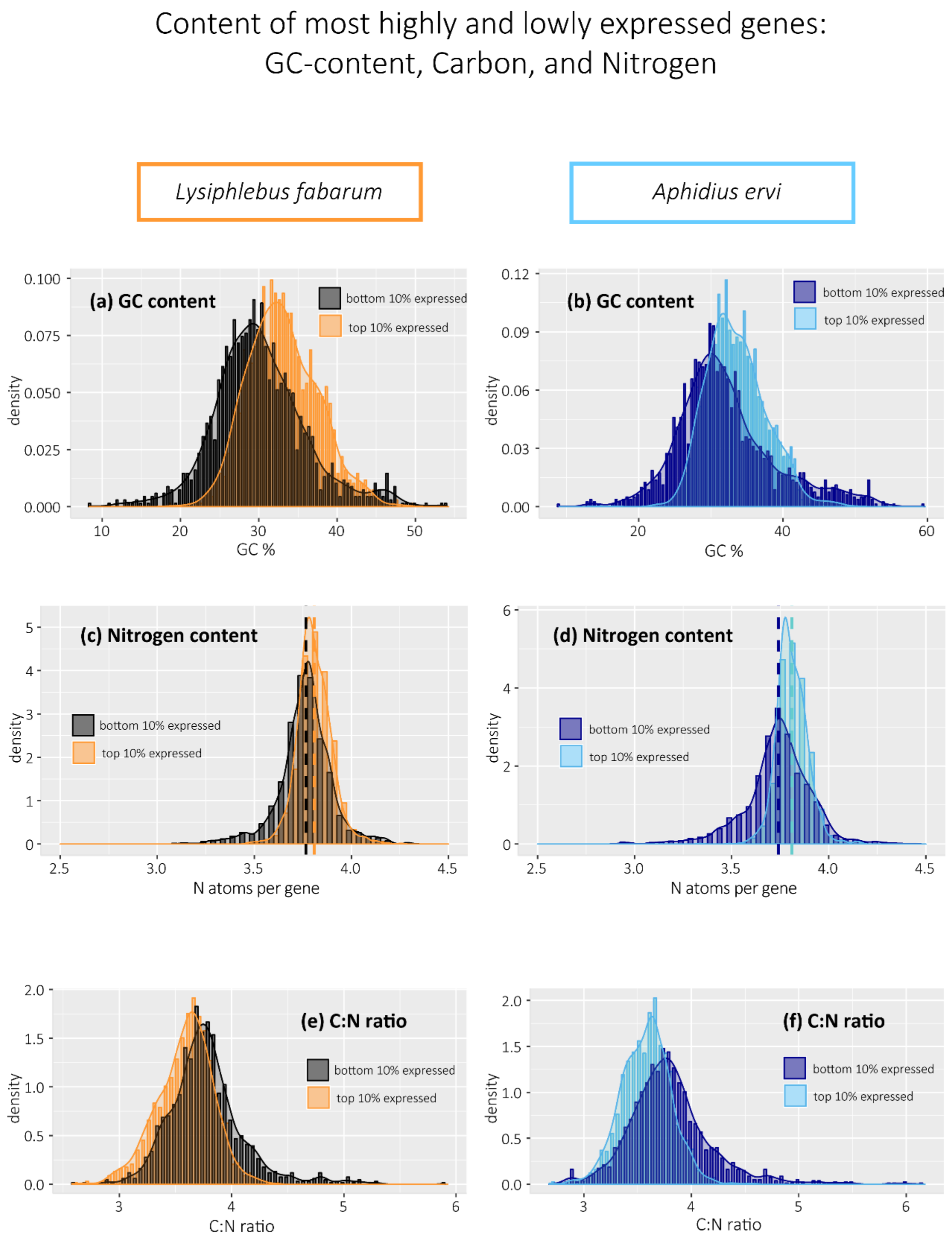

Gene family evolution

Results from orphan gene analysis. Fasta files of these nucleotide sequences are available in Additional Files 4 and 5.

Supplementary Table 11: Summary of orphan gene analysis

	A. ervi	*L. fabarum*
Unassigned genes from Orthofinder	8,717	4,016
Unassigned genes with no hits in the nr/nt/swissprot *databases (June 2019)*	4,306	1,280
Orphan genes with transcriptomic support	2,568	968
Maximum length	5,844 bp	2,514 bp

Comparative orthology with OMA

Results of OMA analysis, which identified putative expansions in gene families in *L. fabarum* and *A. ervi*, relative to another. To examine only the groups with putative expansions or contractions, we looked at the Hierarchical Ortholog Groups (HOGs) with unequal sizes between the two taxa. There were 865 of these groups in *A. ervi* and 223 in *L. fabarum* (Supplemental Figure 14). Among these, only a few (ten groups) had more than 20 genes. We further examined these as putatively interesting in association with adaptive evolution in the two groups (Supplemental Figure 15). Among these, the four largest groups were identified as F-box homologs / Leucine-rich-repeats (Hereafter: F-box/*LRR*), and each of these groups was larger in *A. ervi* than in *L. fabarum*.

Among these, there is some spatial clustering across the genome. The greatest number of these genes appear on Chromosome 1 of *L. fabarum* (26 genes), with 12 of gene less than 10kb apart. In *A. ervi* there are 166 *LRR*s in the scaffolds that are syntenic to Chromosome 1, and these are often highly clustered (59 are less than 100kbp apart, ten are less than 1,000bp apart, Additional File 6). Additional members of this functional group were identified on 125 scaffolds in *A. ervi* and 43 scaffolds in *L. fabarum* (Additional File 6).

Because the *A. ervi* assembly contains more small pieces than *L. fabarum*, we wanted to exclude the possibility that this explains the differences in the number of predicted F-box/LRR genes in these two genomes. To explore this, we examined the size of scaffolds holding the genes predicted to encode F-box/LRR proteins. We conclude that the most abundant F-box/LRRs in *A. ervi* do not appear to be the product of its more fractured assembly. In fact, scaffolds containing LRRs in *A. ervi* are significantly larger than those in *L. fabarum* (Supplemental Figure 16, Welch two-sampled t-test, p=0.001018)

Supplementary Figure 14: Size of HOGs and identity of those containing >20 genes

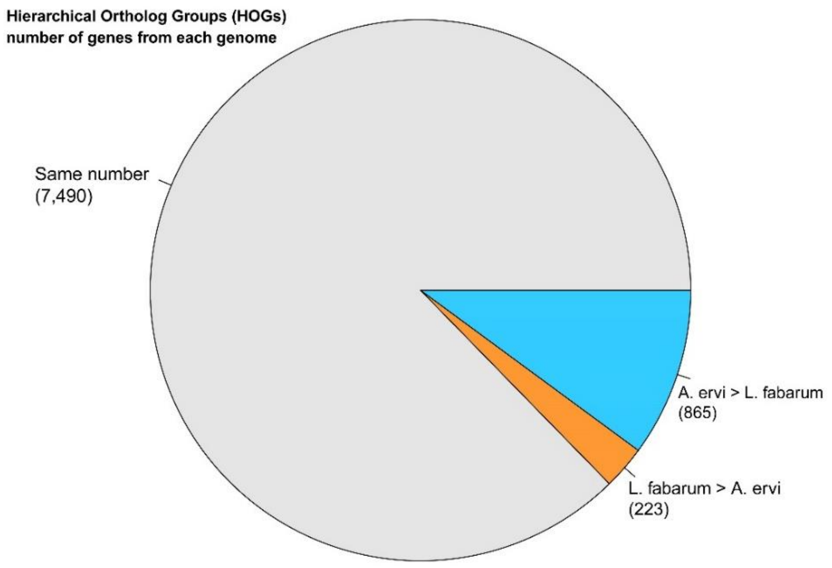

Supplementary Figure 15: Summary of size of HOGs, and annotation information for the ten largest groups (i.e. all groups with more than 20 genes)

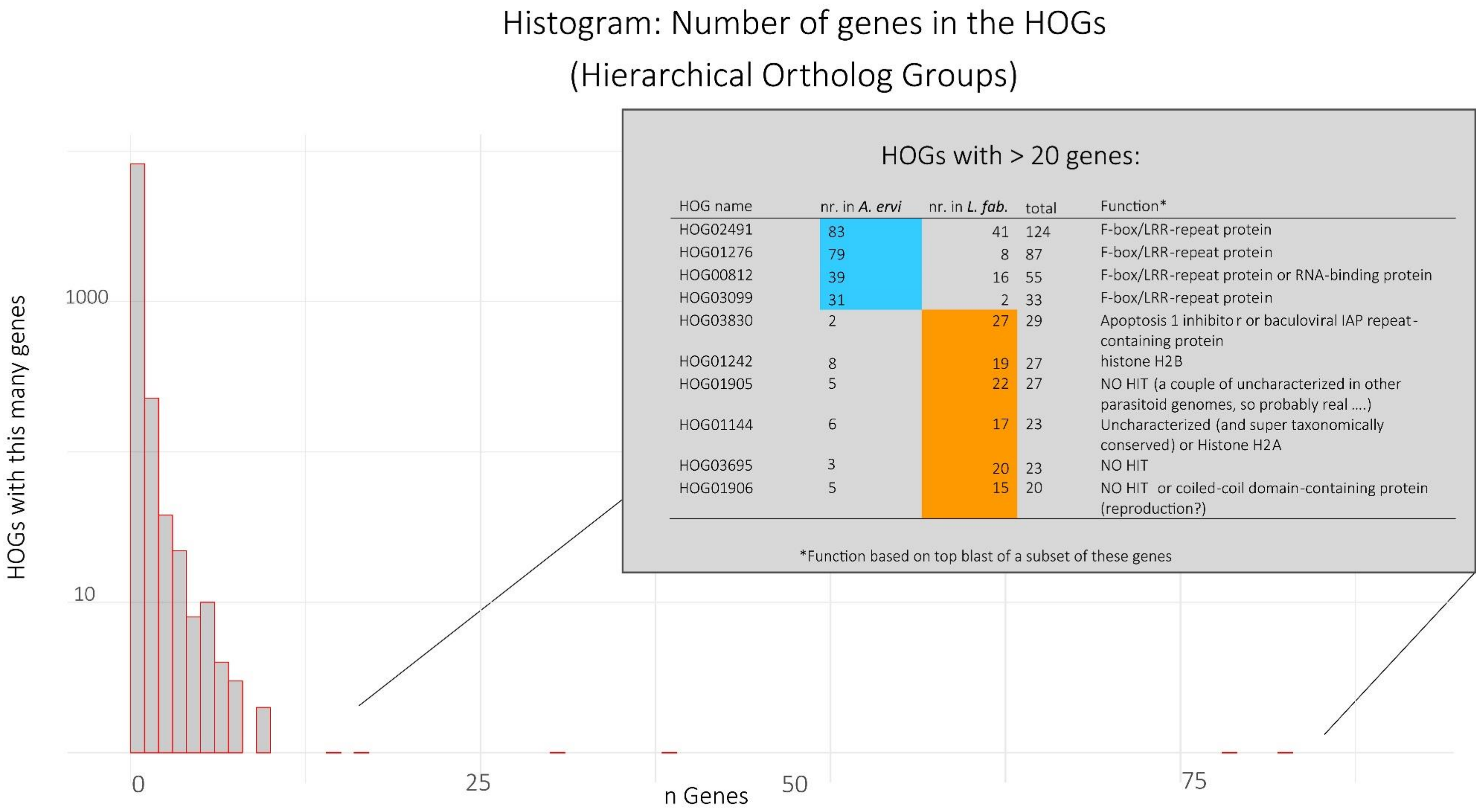

HOG name	nr. in *A. ervi*	nr. in *L. fab.*	total	Function*
HOG02491	83	41	124	F-box/LRR-repeat protein
HOG01276	79	8	87	F-box/LRR-repeat protein
HOG00812	39	16	55	F-box/LRR-repeat protein or RNA-binding protein
HOG03099	31	2	33	F-box/LRR-repeat protein
HOG03830	2	27	29	Apoptosis 1 inhibitor or baculoviral IAP repeat-containing protein
HOG01242	8	19	27	histone H2B
HOG01905	5	22	27	NO HIT (a couple of uncharacterized in other parasitoid genomes, so probably real ....)
HOG01144	6	17	23	Uncharacterized (and super taxonomically conserved) or Histone H2A
HOG03695	3	20	23	NO HIT
HOG01906	5	15	20	NO HIT or coiled-coil domain-containing protein (reproduction?)

*Function based on top blast of a subset of these genes

Supplementary Figure 16: Size distribution of scaffolds containing F-box/LRR proteins

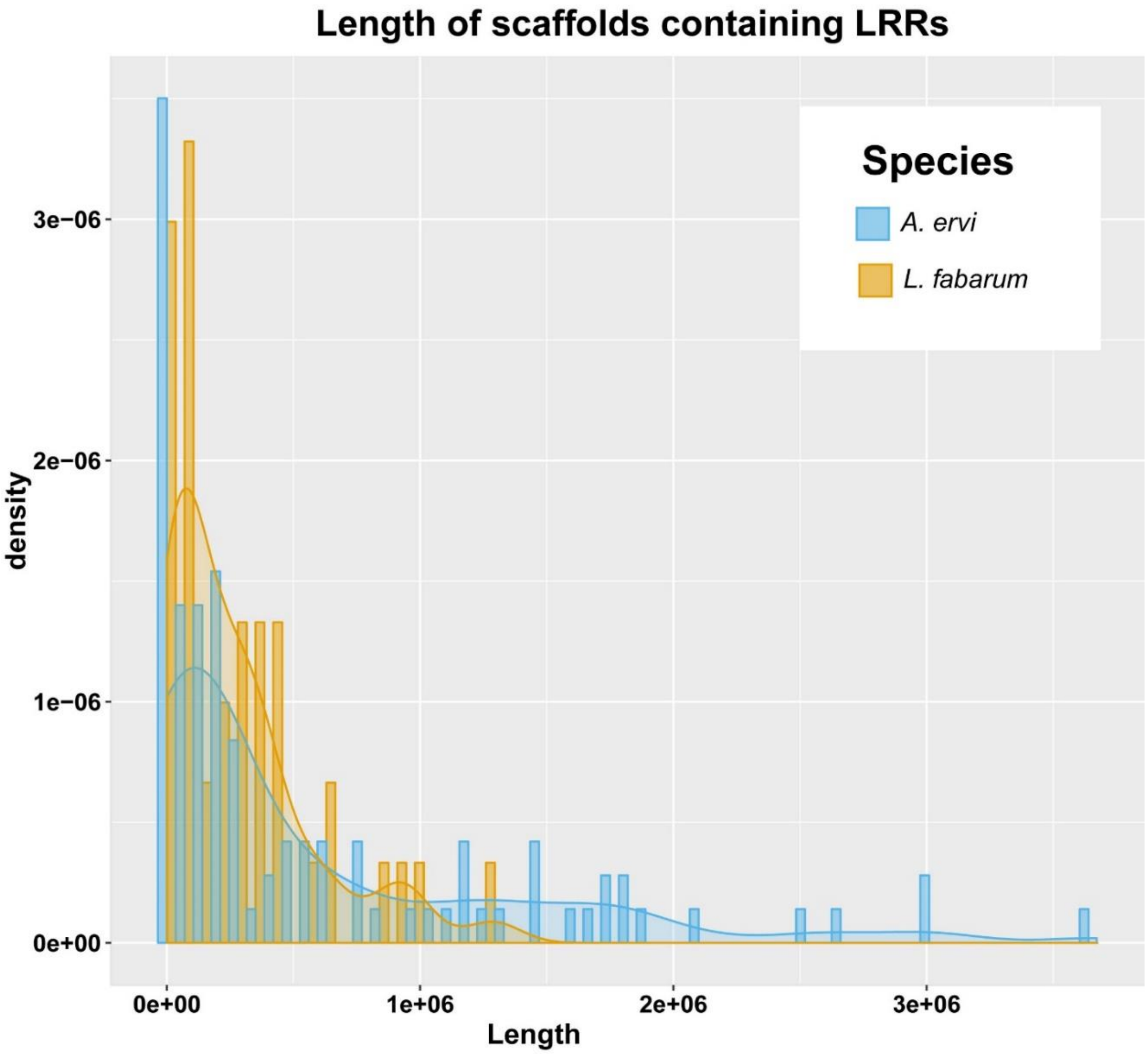

Venom apparatus

We analyzed the venom proteome in *L. fabarum* using the complete venom glands, including tissues (Supplementary Figure 17). After 1D gel electrophoresis, the 16 major bands (Supplementary Figure 18) were excised and analyzed by mass spectrometry. A combined analysis of the genomic/transcriptomic and proteomic data resulted in the identification of 35 putative venom proteins (Supplementary Data 9). Since a number of typical cellular proteins (e.g. actin or myosin) that were identified probably came from venom gland tissues, we only considered putative venom proteins to be the sequences that were: (1) found in proteomics of the venom glands, and (2) either predicted to be secreted or for which the presence of a signal peptide was not tested due to the incompleteness of the sequence. Only 16 putative venom proteins were found in previous work that analyzed of venom proteins for *A. ervi*, but the former approach was more conservative since a protein was considered as venom only if it was found in proteomics not only of the venom glands but also of the reservoir (Colinet *et al.* 2014). In this work, we applied the less conservative criteria described above to the *A. ervi* previous data. This resulted in the identification of 32 putative venom proteins, a similar number to that found for *L. fabarum*. Therefore, we have retained these putative venom proteins for both species (32 in *A. ervi* and 35 in *L. fabarum*).

Comparison of *L. fabarum* venomics data with that of *A. ervi* (Colinet, Anselme et al. 2014) revealed that more than 50% of putative venom proteins are shared between the two species when taking into account these less conservative criteria (Figure 4, Supplementary Data 9). Interestingly, a gamma glutamyl transpeptidase (GGT1) is the most abundant protein in venom of both *A. ervi* (Colinet, Anselme et al. 2014) and *L. fabarum* (Supplementary Data 9). As in *A. ervi*, a second GGT venom protein (GGT2) containing mutations in the active site was found in *L. fabarum* (Supplementary Figure 20). Phylogenetic analysis revealed that GGT1 and GGT2 venom proteins of *A. ervi* and *L. fabarum* GGT1 occur in a single clade in which the venom proteins GGT1 and GGT2 group separately, thus suggesting that they originated from two successive duplication events that occurred prior to speciation of both species (Figure 5). As previously shown for *A. ervi* only, GGT venom proteins from *A. ervi* and *L. fabarum* grouped with clade A, among the three distinct phylogenetic clades formed by non-venomous GGT of Hymenoptera (Figure 5, and Colinet, Anselme et al. 2014). In agreement with this, a similar exon structure was observed between the venomous and non-venomous GGT proteins in clade A, with the exception of exon 1 corresponding to the signal peptide of the venomous GGT proteins (Supplementary Figure 19).

Supplementary Figure 17: Venom apparatus of L. fabarum. Photo by Jean-Luc Gatti.

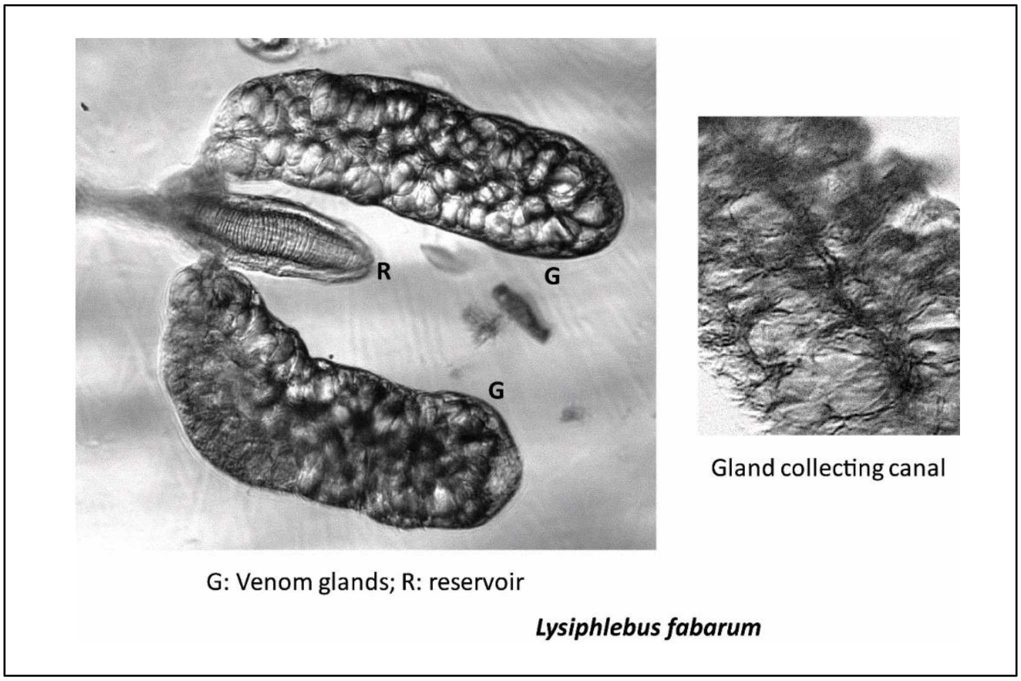

Supplementary Figure 18: SDS PAGE of the L. fabarum *venom gland (G) extract. SDS PAGE 12 %, MW, molecular weight standard in kDa. Numbers 1-16 (right side) indicate the 16 most visible bands, which were analyzed by mass spectrometry.*

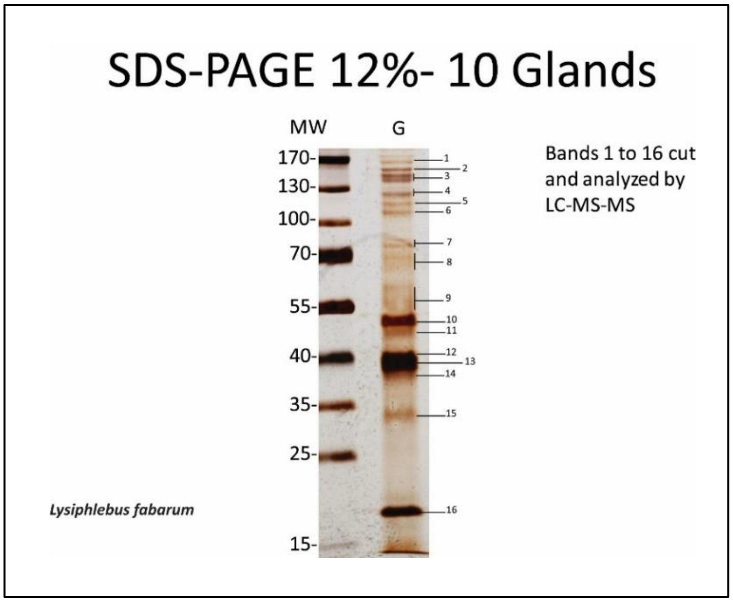

Supplementary Figure 19: Exon structure of gamma glutamyl transferase (GGT) genes (venom components)

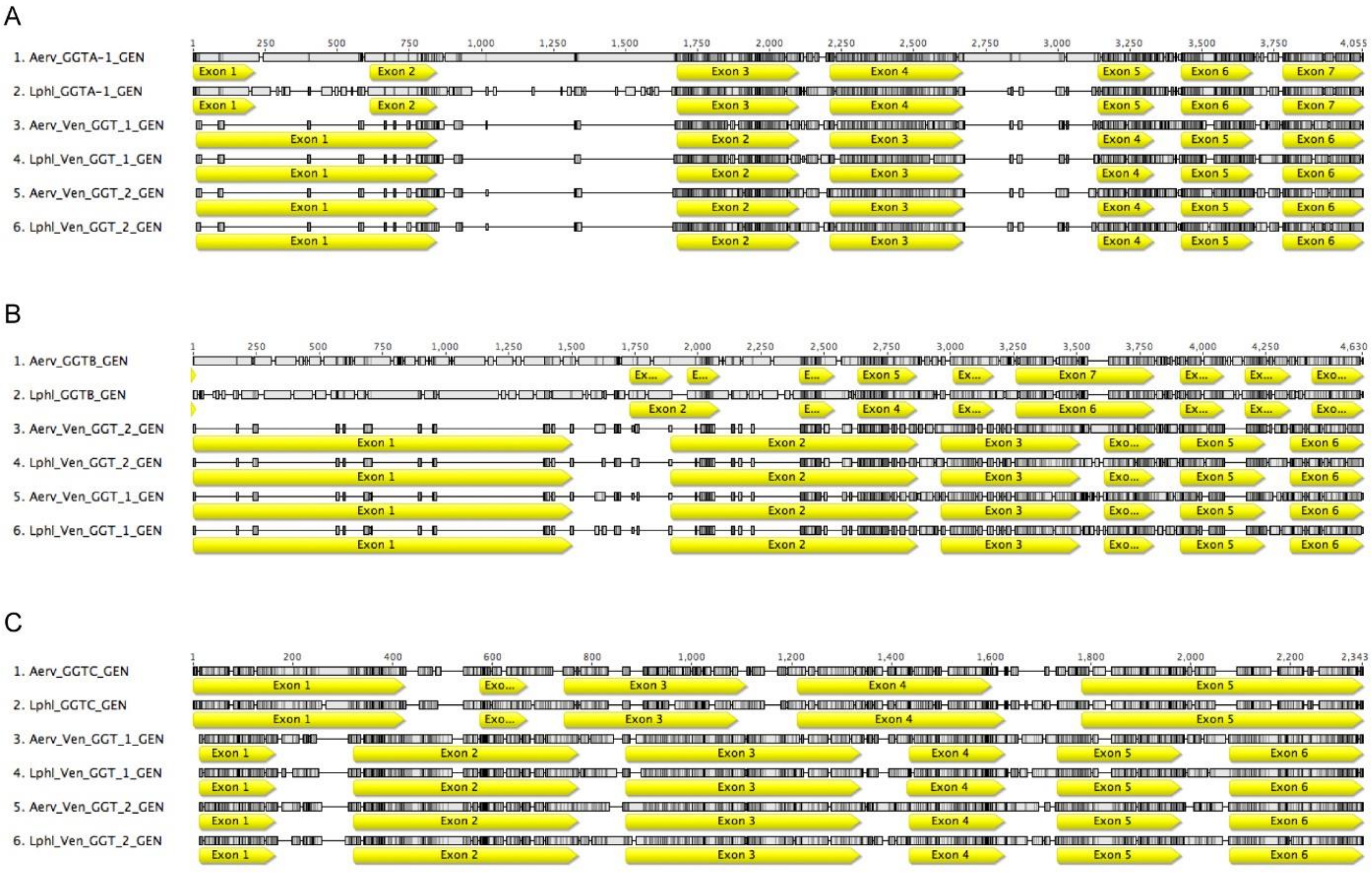

Supplementary Figure 20: Multiple sequence alignment of venom GGT sequences with the human GGT1 sequence. Stars indicate mutations in the venom GGT_2 sequences described to affect the enzymatic activity of human GGT1. The red rectangle indicates the location of the predicted signal peptide in the venom GGT sequence.

```
Aerv_Ven_GGT_1    1 MFSKYILTTTVALVLLKYQCY------SADP-SVSAQFKKSAVCAGANKCAEIGSSTLNN
Lphl_Ven_GGT_1    1 MFSKYILTTTVALVLLKYQCY------SADP-SVSGQFKKSAVCAGANKCAEIGSSTLNN
Aerv_Ven_GGT_2    1 MITKFLLS-MIALIFLKYQCN------CETPESVSRNFKKAAVCAGVDECAEIGLSILKK
Lphl_Ven_GGT_2    1 MIRKLILL-IITLMFLKHQCY------CETTSSISRNFKKAAVCAGADQCADIGLLILKK
HSAP_GGT1         1 MKKKLVVLGLLAVVLVLVIVGLCLWLPSASKEPDNHVYTRAAVAADAKQCSKIGRDALRD

Aerv_Ven_GGT_1   54 GGSAVDAAIATMICNNLVHPHLAGYGGGFFMTVYDRANKNVDFLNAREKAP--------G
Lphl_Ven_GGT_1   54 GGSAVDAAIATMICNNLVHPHLAGYGGGFFMTIYDRSSKNVDFLNAREKAP--------S
Aerv_Ven_GGT_2   54 GGTAVDSAIATMICNGLIHMHIAGYGGGFFMTIYQSDTKKVSFLNAKEKSPKETSEYSYK
Lphl_Ven_GGT_2   54 GGTAVDTAIATMICNGLIHMHMAGYGGGFFMTIYQSDTKKVSFLNAKEISPNDTDEISYK
HSAP_GGT1        61 GGSAVDAAIAALLCVGLMNAHSMGIGGGLFLTIYNSTTRKAEVINAREVAPRLAFATMFN
                                                                 *

Aerv_Ven_GGT_1  106 DISKASKTGVNSIAVPGEIAGYGVAHKKYGKLSWEKLFEPTIELCESGYQISKALAKAIA
Lphl_Ven_GGT_1  106 DVSKATKSGVNSIAVPGEIAGYELAHKKYGKLSWEKLFEPTIQLCENGFQVSKALKKAID
Aerv_Ven_GGT_2  114 QLPLDEKTGL-RIAVPGEIAGYAKAHEKFGKIPWSDLFQPTIDLCKKGFKITKTLHDALE
Lphl_Ven_GGT_2  114 DIEPNERNGL-KIAVPGEIAGYAEAHKKFGKILWSELFQPTIDLCKKGYTITKTLSDALK
HSAP_GGT1       121 SSEQSQKGGL-SVAVPGEIRGYELAHQRHGRLPWARLFQPSIQLARQGFPVGKGLAAALE

Aerv_Ven_GGT_1  166 SSADTIKQDDTLTALYT----NGGALKKEGDTVNPGKLCDTLKTIASGGADEFYKGDLAS
Lphl_Ven_GGT_1  166 ESKDIINANENLKSLYA--------SKNEGDLIKPGKLCETLKKIASGGANAFYKGEVGT
Aerv_Ven_GGT_2  173 KSQSDIENDETLRELFMDKETNSQSLKREGDLVKPTALCETLVTIASKGAGEFYNGTLAK
Lphl_Ven_GGT_2  173 KSQEVIMKDETLRELFMDK--NTELLKKQGDTVNPTNICDTLNTIAKEGANAFYNGTLSK
HSAP_GGT1       180 NKRTVIEQQPVLCEVFC----RDRKVLREGERLTLPQLADTYETLAIEGAQAFYNGSLTA

Aerv_Ven_GGT_1  222 SIIGDLSKKSSALTKDDLSSYTAKWAS-PLKTTLLNDLTLYTANAPGGGAPLALALNIID
Lphl_Ven_GGT_1  218 SIAGDIRKKSGALTKDDLSSYTAEWST-SLKTTLLNDLKLHTANAPGGGATLALCLNIID
Aerv_Ven_GGT_2  233 TIVEDLQSQSSIITEDDFSDYEAEWLE-PLSIKLSNDLTLHTSSVPSSGGLLTLIMNILN
Lphl_Ven_GGT_2  231 TIIEDLKNENSIITEDDLSNYNAEWMK-PLSISLSNNLTLHTSNVPSSGGLLGLMMNVLD
HSAP_GGT1       236 QIVKDIQAAGGIVTAEDLNNYRAELIEHPLNISL-GDVVLYMPSAPLSGPVLALILNILK

Aerv_Ven_GGT_1  281 EYGNDLVSSDAA----LKLHRLSEIWKYSVAAKSKLGDPDVSS--LDELLKTITSDAYAK
Lphl_Ven_GGT_1  277 ELKLNPSSLNLP----STLNSLAEALKHCVARKLSLGDPKSNAQ-VEKEVKTMISDALAK
Aerv_Ven_GGT_2  292 EFNFTPNSLNGTDNTSLTYHKIMESFKWSFPQKYKLGDSKFSKKILDLLVNEFTSKDYAK
Lphl_Ven_GGT_2  290 DINFNSSSLNGTKNTALTYHLIMETWKWTSAQLYKLGDPKFSDKMFDLLAQEFTSKDFAK
HSAP_GGT1       295 GYNFSRESVESPEQKGLTYHRIVEAFRFAYAKRTLLGDPKFVD--VTEVVRNMTSEFFAA

Aerv_Ven_GGT_1  335 EIKSKINDKETSHEASHYGVDVDVQDE-GTAQVSIIDADGNAVSATSSLNGVFGSGVVSE
Lphl_Ven_GGT_1  332 EIRSKINDEKTSNDPKHYGADVDVQDD-GTAQVSIIDSNGNAVSATSSLNGPFGSGVVSE
Aerv_Ven_GGT_2  352 SIKKKINEKKTSNKESDYGGKESVEDQ-GTAQVSVIDSNGNAVSATSSINAPFGSGIVSK
Lphl_Ven_GGT_2  350 SIKNKINYEKTSNTAKTYGGKESVQDH-GTAQVSVIDSDGNAVSATSSINSPFGSGIVSK
HSAP_GGT1       353 QLRAQISDD-TTHPISYYKPEFYTPDDGGTAHLSVVAEDGSAVSATSTINLYFGSKVRSP

Aerv_Ven_GGT_1  394 STGIILNSALSDF-----------SPKSKANEIAPNKRPLSSMAPSIIVDSNDNVKLVIG
Lphl_Ven_GGT_1  391 STGIILNSALSDF-----------TPKSSANSLAPGKRPLSSMAPSIIIDSSDNVRLVIG
Aerv_Ven_GGT_2  411 RTGLIFNNAMDGFWTPTVTGPTGGEETQDGNRIDAQKKPLSSMLPTIITDSNGDVKVVIG
Lphl_Ven_GGT_2  409 RTGLIFNNAMDGFWIPTITGTTDKLTEIDGNRIDANKRPLSSMVPSIITDSNGDVKVVIG
HSAP_GGT1       412 VSGILFNNEMDDFSSPSITNEFG-VPPSPANFIQPGKQPLSSMCPTIMVGQDGQVRMVVG
                               *

Aerv_Ven_GGT_1  443 ATGGAKITTAVSSVLARYLWSKQGLKEAVDAPRLHQEIFPMELSYEE-SSDVITQLKEVY
Lphl_Ven_GGT_1  440 ATGGPKITTAVSLVLARYLWLKQGLQEAVDAPRIHVSLFPMELSYEK-SDDIISKLKTVY
Aerv_Ven_GGT_2  471 GTGGTKIITSVSFVLARYLWMGEDMKTAIDADRIHYQDIIMRVGAEK-MDDVVQNLKRKY
Lphl_Ven_GGT_2  469 GTGGTRILTSVSFVLARYLWMGEDMKTAIDANRIHYRPNVMILRTET-IDDIIKYMKS-Y
HSAP_GGT1       471 AAGGTQITTATALAIIYNLWFGYDVKRAVEEPRLHNQLLPNVTTVERNIDQAVTAALETR

Aerv_Ven_GGT_1  502 KHNTVEMKGITSAICALSREGDSILGVADGRRGGSVQGSN-
Lphl_Ven_GGT_1  499 GHNTVEMKGITSAVCALAREGDHILGVADGRRGGSVKGSN-
Aerv_Ven_GGT_2  530 GHRVVLLKEPHSAICSLSKENGVINGVADGRRGGNVAGIDE
Lphl_Ven_GGT_2  527 GHRTALLKDPDSSICSLTKENNIINGVADGRRGGNIAGIDE
HSAP_GGT1       531 HHHTQIASTFIAVVQAIVRTAGGWAAASDSRKGGEPAGY--
```

<u>Cuticular hydrocarbon profiles of *A. ervi* and *L. fabarum*</u>

Supplementary Figure 21: Characteristic chemical profile based on a pool of 135 individual Lysiphlebus fabarum (in 60ul) and table summarizing average composition of CHC compounds.

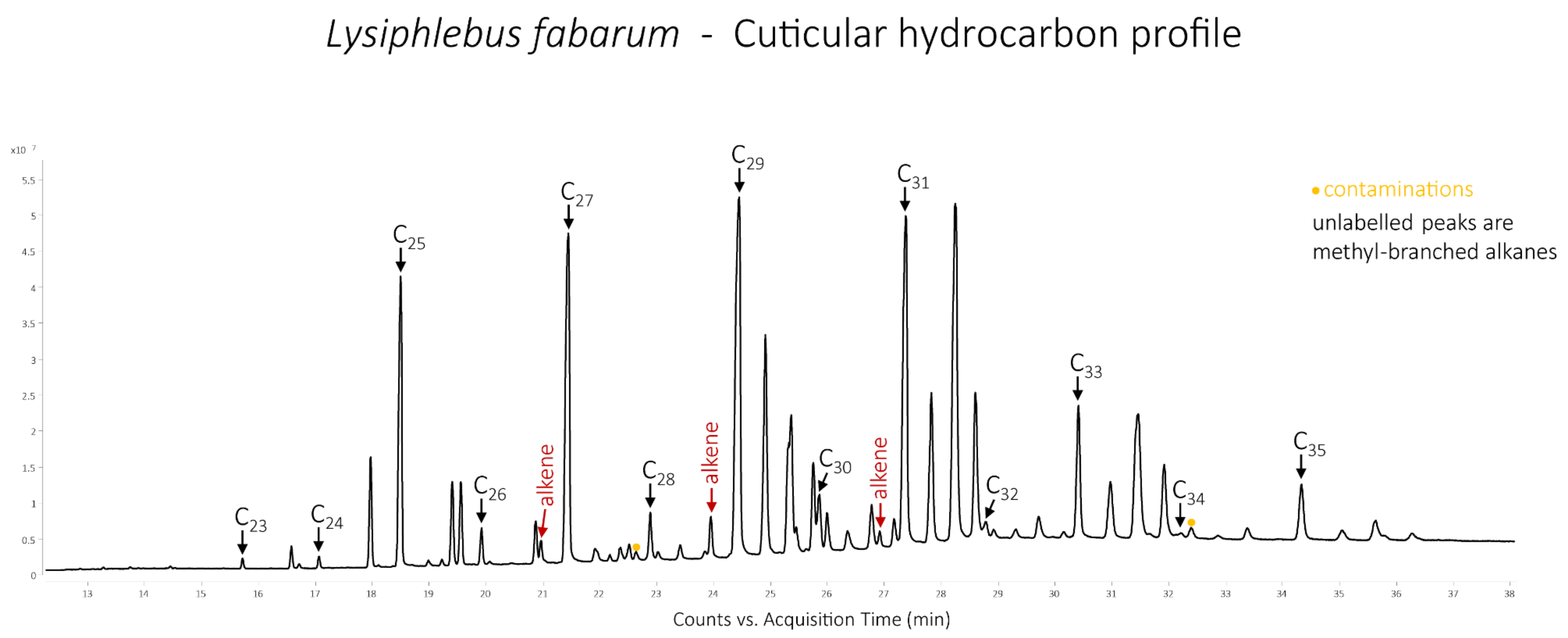

Average CHC compound class ratios (%)

Alkanes	Methyl-branched alkanes	Alkenes	Dienes
54.17	44.32	1.5	0

Supplementary Figure 22: Characteristic chemical profile of an individual Aphidius ervi *female with identifications of the most abundant compounds, and table summarizing average composition of CHC compounds.*

Aphidius ervi - Cuticular hydrocarbon profile

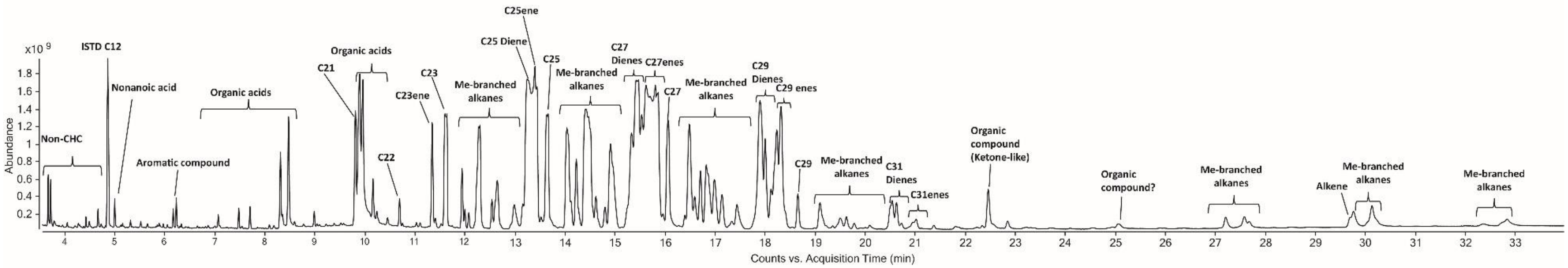

Average CHC compound class ratios (%)

Alkanes	Methyl-branched alkanes	Alkenes	Dienes
9.77	18.23	29.73	38.48

Supplementary Figure 23: Comparison of the above CHC profiles from A. ervi *and* L. fabarum*. Note, that these are aligned for comparative purposes only and as such, the abundance axis has been removed. The traces were produced on different machines and using pooled individuals for* L. fabarum *and a single individual for* A. ervi.

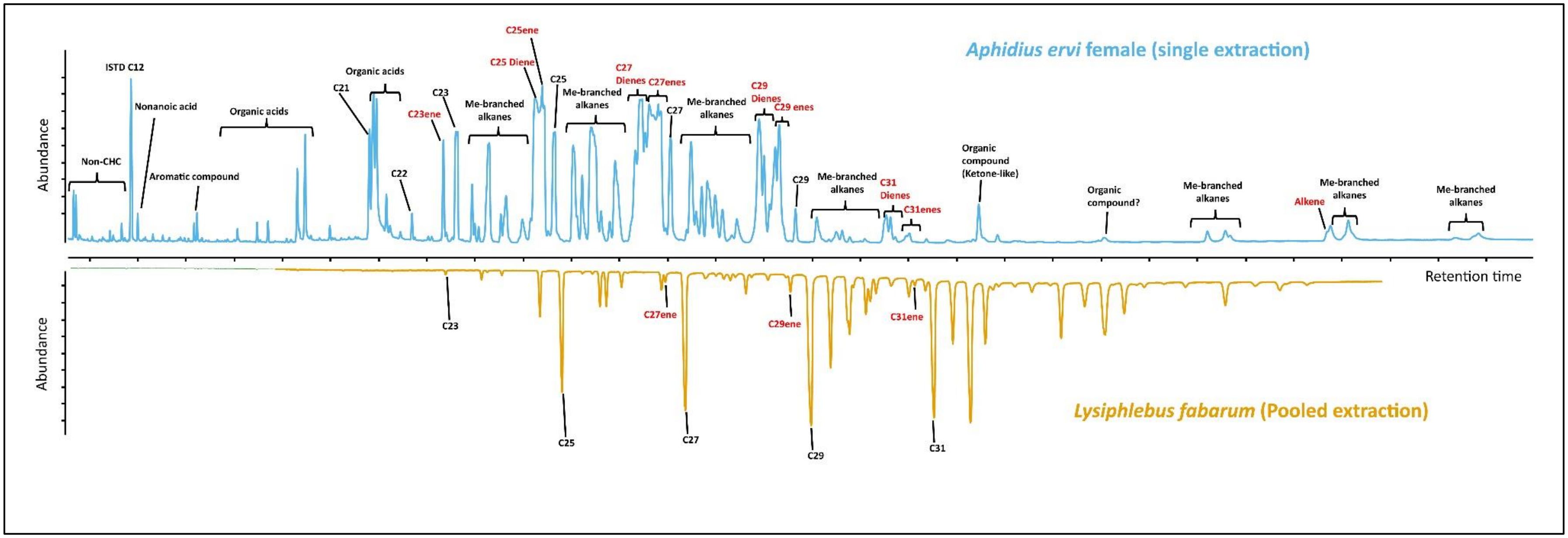

Community annotation of individual gene families

Desaturase genes

Membrane-bound desaturases are essential for their role in biological processes including lipid metabolism, cell signaling, and lipid membrane fluidity (Hazel and Williams 1990; Miyazaki and Ntambi 2003; Pyne and Pyne 2017) by introducing carbon-carbon double bonds into fatty acyl chains (Los and Murata 1998). In addition to these basic functions, desaturase genes are also well characterized for their role in semiochemical biosynthesis in solitary insects where they contribute to the production of cuticular alkenes involved in courtship behavior in *Drosophila* (Dallerac, Labeur et al. 2000; Fang, Takahashi et al. 2002; Labeur, Dallerac et al. 2002; Chertemps, Duportets et al. 2006; Bousquet, Nojima et al. 2012) as well as the synthesis of volatile sex pheromones used for mate attraction in numerous moth species (Roelofs and Rooney 2003; Moto, Suzuki et al. 2004; Xue, Rooney et al. 2007). Given their role in insect recognition systems, they make up an important gene family for understanding the evolution and diversity of insect semiochemicals possessing carbon-carbon double bonds. Here we investigated the diversity of acyl-CoA desaturase genes in *A. ervi* and *L. fabarum* for the purpose of understanding basic features of their evolution (e.g., gene numbers, gene synteny, and homology) in comparison with thirteen other insects. Moreover, notable differences in cuticular hydrocarbon (CHC) profiles have been observed between *A. ervi* and *L. fabarum* implicating their potential role in host-parasite evolution. In particular, the CHC profiles of *L. fabarum* mimic their aphid host, *Aphis fabae*, which is hypothesized to allow *L. fabarum* to evade attacks by honeydew-collecting ants that tend the aphids (Liepert and Dettner 1993; Liepert and Dettner 1996). *Lysiphlebus fabarum,* CHCs are characterized predominantly by saturated hydrocarbons and contain less than 2% alkenes (Supplementary Figure 21). *Aphidius ervi* females, on the other hand, are oligophagous parasites of Macrosiphini aphids (Monticelli, Nguyen et al. 2019) and their CHCs are composed of nearly 60% alkenes (Supplementary Figure 22). These strong CHC differences between the two parasitoids (Supplementary Figure 23) may help highlight candidate desaturase genes involved in cuticular alkene variation and, in turn, potential characteristics of host-parasite evolution, among other functions.

We identified a total of 16 and 15 prospective desaturase genes in the genomes of *A. ervi* and *L. fabarum* respectively (Supplementary Table 12). Of these, two genes in *A. ervi* were only partial (i.e., <25% gene coverage), and both contained premature stop codons indicating that they are likely pseudogenes. Similarly, in *L. fabarum* we identified four partial genes, two of which are likely pseudogenes due to premature stop codons, and the other two of which may be either pseudogenes or were poorly constructed due to missing sequence data or assembly errors. Searches of the unassembled genomic data did not reveal additional desaturase gene sequences for either species. Taken together, our annotation analyses resulted in 14 full-length desaturase genes for *A. ervi* and 11 for *L. fabarum*. Searches against the conserved domain database (CDD) determined that both species possess single, full-length copies of three desaturase gene subfamilies: infertile crescent (*ifc*), with Delta4-sphingolipid-FADS-like domains; Cytochrome b5-related (*Cyt-b5-r*), with Cyt-b5 & FA_desaturase domains; and Ergosterol $\Delta^{5,6}$-Desaturase (*ERG3*), with ERG3 domains. Annotation of *L. fabarum* also identified an additional *Cyt-b5-r* gene fragment (i.e., 63 AA, or <15% coverage), but this fragment is poorly supported by transcript evidence and is missing the fatty acid desaturase domain, suggesting that it is likely a pseudogene. The remaining

24 genes (including two partial genes and three pseudogenes) belong to the First Desaturases (Hashimoto, Yoshizawa et al. 2008) which possess Delta9-FADS-like domains.

The organization of desaturase genes in the genomes of *A. ervi* and *L. fabarum* showed two regions of synteny, with *A. ervi* exhibiting the larger cluster of desaturases (four genes and one pseudogene within a 14.9 Kbp region of scaffold61) compared with only two genes occurring together in a 5.4 Kbp region of tig00002181 in *L. fabarum*. In both species, the remaining desaturases are scattered across separate linkage groups throughout the genome, although the lack of chromosome-level mapping of these scaffolds prevents us from knowing if some genes may occur at different region(s) within the same chromosome.

Phylogenetic reconstruction of the First Desaturases of *A. ervi* and *L. fabarum* revealed that eight putatively functional genes and one partial gene fall into four well-supported subfamilies (Desat E, Desat D, Desat C, and Desat B) and the remaining 11 putatively functional genes, one partial gene, and one pseudogene fall into the poorly supported paraphyletic subfamilies, Desat A1 and Desat A2 (Supplementary Figure 24, Helmkampf *et al.* 2015). *Aphidius ervi* and *L. fabarum* mirror the simple homologies previously described in subfamilies Desat E and Desat D, as each possess single desaturase genes for both clades. Likewise, both species show losses in the subfamily Desat C, which has a more complex homology represented by gene losses, single-copies, and small expansions across this group. The remaining three First Desaturase subfamilies, Desat A1, Desat A2, and Desat B, are characterized by gene numbers ranging from single copies in some species up to 9 putatively functional copies in others (Helmkampf, Cash et al. 2015). In subfamily Desat A2, *A. ervi* and *L. fabarum* both possess single copies, which is also true for the jewel wasp, *Nasonia vitripennis* and several other insects (e.g., *Bombus terrestris*, *Bombyx mori*, and *Drosophila melanogaster*). The homology relationships represented in subfamilies Desat A1 and Desat B, however, differ notably between the two parasitoids. In both cases, *A. ervi* possesses more putatively functional copies of genes in these two subfamilies (five copies of Desat A1 and three copies of Desat B) compared with *L. fabarum* (four copies of Desat A1 and one copy of Desat B). Whether these differences in gene numbers were the result of gains in the lineage containing *A. ervi* or losses in the lineage containing *L. fabarum* could not be conclusively determined, and future analyses could potentially resolve this with the addition of other closely related taxa. Nevertheless, these results suggest that differences in gene number may partially contribute to variation in cuticular alkene presence and abundance in *A. ervi* and *L. fabarum*, although other factors such as mechanisms regulating gene expression and alternative splicing cannot be ruled out. Future work on desaturase genes in *A. ervi* and *L. fabarum* and their roles in CHC biosynthesis will benefit from investigating these open questions and experimental studies may be of particular interest for genes in the Desat A1 and Desat B subfamilies where these copy number differences occur.

Supplementary Table 12: Annotation table of acyl-CoA and sterol desaturase genes in Aphidius ervi *and* Lysiphlebus fabarum

Species (Genome assembly)	Official Gene Set / Transcript ID	Gene name[1]	Peptide length [AA]	Linkage group(s)	Region [bp], strand	Protein domain (CD-Search)
Aphidius ervi	AE3019260	Aerv_desatE_a	320	scaffold61 & 5487	154512-156329, -; 355-1034, +	Delta9-FADS-like (cd03505)
(Ae_genome_v3.0)	None	Aerv_desatE_b *,†	70	scaffold13	540600-541033, -	Delta9-FADS-like (cd03505)
	None	Aerv_desatE_c *,†	32	scaffold61	155051-155149, -	Delta9-FADS-like (cd03505)
	AE3010961	Aerv_desatD	345	scaffold138	130913-133548, +	Delta9-FADS-like (cd03505)
	AE3008423	Aerv_desatB_a	309	scaffold61	144776-146415, +	Delta9-FADS-like (cd03505)
	RNA5663_R0_00001	Aerv_desatB_b	308	scaffold61	141393-143379, +	Delta9-FADS-like (cd03505)
	AE3008695	Aerv_desatB_c	333	scaffold59	51684-63834, -	Delta9-FADS-like (cd03505)
	AE3014422	Aerv_desatA2	372	scaffold188	86452-88130, -	Delta9-FADS-like (cd03505)
	AE3010398	Aerv_desatA1_a	338	scaffold43	201155-202828, +	Delta9-FADS-like (cd03505)
	RNA10122_R0_00001	Aerv_desatA1_b	353	scaffold61	150998-152393, +	Delta9-FADS-like (cd03505)
	AE3005642	Aerv_desatA1_c	341	scaffold25	145319-146784, +	Delta9-FADS-like (cd03505)
	AE3010275	Aerv_desatA1_d	353	scaffold116	77281-80483, +	Delta9-FADS-like (cd03505)
	AE3005215	Aerv_desatA1_e	354	scaffold51	486930-490404, -	Delta9-FADS-like (cd03505)
	AE3009224	Aerv_ifc ‡	321	scaffold44	23655-25563, +	Delta4-sphingolipid-FADS-like (cd03508)
	AE3008566	Aerv_Cyt_b5_r ‡	424	scaffold60	234079-235878, +	Cyt-b5 & FA_desaturase (pfam00173 & 00487)
	AE3008531	Aerv_ERG3 ‡	448	scaffold67	332267-339125, -	ERG3 (COG3000)
Lysiphlebus fabarum	LF008097	Lfab_desatE	323	tig00002181	32817-34667, -	Delta9-FADS-like (cd03505)
(Lf_genome_v1.0)	LF007787	Lfab_desatD	357	tig00002219	15120-17025, +	Delta9-FADS-like (cd03505)
	LF008181	Lfab_desatB_a	333	tig00000279	110474-115994, +	Delta9-FADS-like (cd03505)
	LF014464	Lfab_desatB_b †	151	tig00000955	60-806, +	Delta9-FADS-like (cd03505)
	LF011096	Lfab_desatA2	372	tig00000766	19248-21004, +	Delta9-FADS-like (cd03505)
	LF000460	Lfab_desatA1_a	318	tig00001871	269575-270922, +	Delta9-FADS-like (cd03505)
	LF006691	Lfab_desatA1_b †	200	tig00002054	141926-142652, -	Delta9-FADS-like (cd03505)
	LF008096	Lfab_desatA1_c	353	tig00002181	29279-30712, +	Delta9-FADS-like (cd03505)
	LF003271	Lfab_desatA1_d	354	tig00000512	223440-226094, -	Delta9-FADS-like (cd03505)
	LF009934	Lfab_desatA1_e	355	tig00001943	45123-46546, -	Delta9-FADS-like (cd03505)
	None	Lfab_desatA1_f *,†	81	tig00000524	67863-68194, +	Delta9-FADS-like (cd03505)
	LF004999	Lfab_ifc ‡	321	tig00000335	214481-216184, +	Delta4-sphingolipid-FADS-like (cd03508)
	LF004369	Lfab_Cyt_b5_r_a ‡	425	tig00000307	267035-268762, -	Cyt-b5 & FA_desaturase (pfam00173 & 00487)
	LF008817	Lfab_Cyt_b5_r_b *,‡	63	tig00001895	39192-39380, +	Cyt-b5 (pfam00173)
	LF003650	Lfab_ERG3 ‡	448	tig00000377	52559-54934, -	ERG3 (COG3000)

[1] Following the phylogeny-based desaturase gene nomenclature of Helmkampf et al. (2015).

* Putative pseudogenes designated as such due to a combination of premature stop codons, missing sequence, and inconclusive evidence of gene expression.

† Partial genes that were excluded from the First Desaturase phylogenetic analysis due to too little sequence data (i.e., <250 AA).

‡ Putatively functional desaturase genes that were excluded from the First Desaturase phylogenetic analysis due to gene subfamily differences.

Supplementary Figure 24: Phylogenetic reconstruction of first desaturase genes in A. ervi and L. fabarum in comparison with 13 other insect species. Unrooted maximum likelihood tree based on 157 genes in 15 species, with support values (>50%) based on 200 rapid bootstrap replicates. Gene names follow the updated nomenclature proposed in Helmkampf et al. *(2015), except for genes that have been previously characterized in other studies (these gene names are in black bold). Species are indicated by four-letter prefixes as follows: Acpi = Acyrthosiphon pisum, Aerv = Aphidius ervi (gene names in bold color), Amel = Apis mellifera, Agam = Anopheles gambiae, Bmor = Bombyx mori, Bter = Bombus terrestris, Cflo = Camponotus floridanus, Dmel = Drosophila melanogaster, Hsal = Harpegnathos saltator, Lfab = Lysiphlebus fabarum (gene names in bold color), Lhum = Linepithema humile, Pbar = Pogonomyrmex barbatus, Nvit = Nasonia vitripennis, Tcas = Tribolium castaneum, and Znev = Zootermopsis nevadensis.*

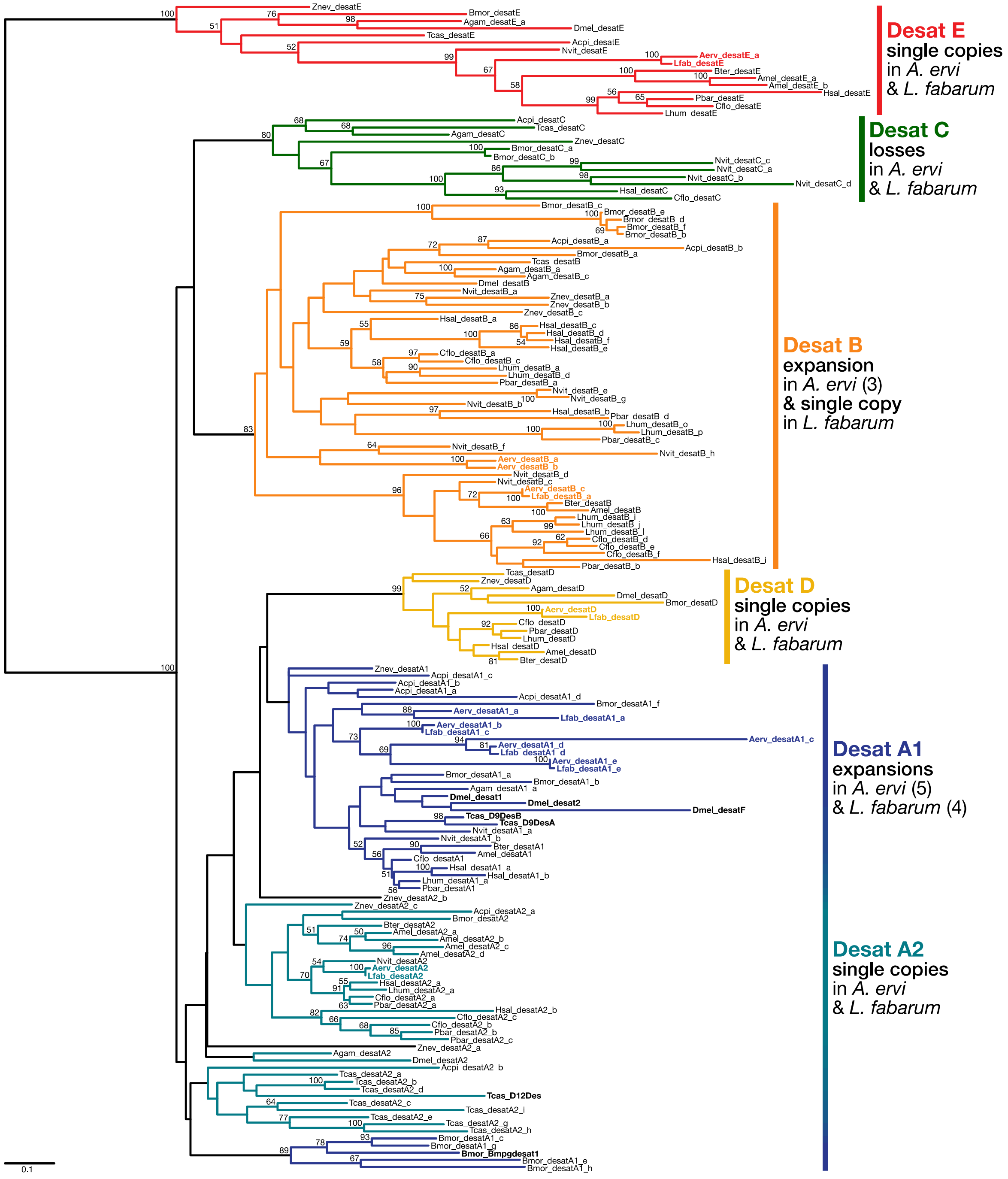

Immune genes

The *Drosophila melanogaster* Toll pathway (Supplementary Figure 25), initially identified as a developmental pathway acting via the nuclear factor kappa B (NF-κB), is essential for the response to fungi and Gram-positive bacteria (Valanne, Wang et al. 2011) and it acts via the nuclear factor kappa B (NF-κB, Supplementary Figure 25). Toll-like receptors (TLRs) in this pathway are activated by a cleaved form of an extracellular cytokine-like polypeptide, *spätzle*. Proteolytic cleavage of *spätzle* is the result of a cascade that is initiated by recognition of micro-organisms via pattern-recognition receptors (PRRs, e.g. peptidoglycan recognition proteins, or PGRPs and β-glucan recognition proteins, or βGRPs).

The Imd/NF-kappa-B pathway (named for the immune deficiency *imd* gene) is pivotal in the humoral and epithelial immune response to Gram-negative bacteria (Supplementary Figure 25). Signaling through *imd* (a death domain protein) ultimately activates the transcription of specific antimicrobial peptides (AMPs, Myllymäki, Valanne et al. 2014). It is activated when peptidoglycan recognition proteins of the *PGRP-LC* and *PGRP-LE* receptors bind to the bacterial peptidoglycan. This triggers signaling to the NF-κB transcription factor *relish*, via the Fas-associated protein with death domain (FADD), the death-related ced-3/Nedd2-like protein (*DREDD*), and the transforming growth factor beta (TGF-β)-activated kinase 1 (*TAK1*), inhibitor of κB kinase (*IKK*) pathways (Lemaitre and Hoffman 2007; Charroux and Royet 2010; Buchon, Silverman et al. 2014). DREiDD cleaves *imd*, which enables its association and activation by the ubiquitin E3-ligase *DIAP2*. *DREDD* also cleaves the Relish precursor, allowing its phosphorylation and translocation into the nucleus where it activates the transcription of specific antimicrobial peptides (AMP).

The JAK-STAT pathway is involved in the humoral and cellular immune response (Morin-Poulard, Vincent et al. 2013). The JAK-STAT pathway is activated after binding of cytokine-like proteins called unpaired (*upd*, *upd2*, and *upd3*) to the receptor *Domeless* (Dome). Activated JAKs phosphorylate each other and provide binding sites for the Src homology 2 (*SH2*) domains of *STAT* molecules. The *STATs* are then phosphorylated by the *JAKs* and translocate into the nucleus, where they bind the promoters of target genes. Supplementary Table 13 compares the actors of these major pathways between *A. ervi*, *L. fabarum* and well-studied insect models.

Supplementary Table 13: Comparison of some immune associated genes in A. ervi, L. fabarum *and selected insect models. Known copy number for each gene (adapted from: Evans, Aronstein et al. 2006; Gerardo, Altincicek et al. 2010; Arp, Hunter et al. 2016).*

		D. melanogaster	*A. mellifera*	*A. pisum*	*A. ervi*	*L. fabarum*
Recognition	PGRP	13	4	0	0	0
	GNBP	3	2	2	1	1
	DSCAM	1	1	1	1	1
Toll	Toll	9	5	7	5	5
	MyD88	1	1	1	1	0
	Tube	1	1	1	0	1
	Pelle	1	1	1	1	1
	Cactus	1	3	1	1	1
	Dif/Dorsal	2	2	2	1	1
	Spätzle	6	2	10	1	1
Imd	Imd	1	1	0	0	0
	Dredd	1	1	0	1	1
	Tak1	1	1	1	1	1
	FADD	1	1	1	1	1
	Relish	1	1	0	1	1
JAK/STAT	Basket (JNK)	1	1	1	1	1
	Hopscotch (JAK)	1	1	1	1	1
	STAT	1	1	2	1	1
Effectors	PPO	3	1	2	3	3
	Defensin	1	2	0	1	0
	Cecropin	5	0	0	0	0
	Lysozyme	13	3	3	2	2
	TEP	6	4	2	5	5
TOTAL		74	40	39	26	25

Supplementary Figure 25: Comparison with the D. melanogaster *IMD and Toll pathways components. Permission to reprint pathway image from 04624 granted by the Kanehisa Laboratories (Kanehisa and Goto 2000). Modifications are our own: Red crosses indicate genes absent in both species; Blue cross, indicate genes absent in* L. fabarum; *Yellow cross, genes absent in* A. ervi; *Green circle indicate genes found present in both species. (KEGG pathways also available at: www.genome.jp/kegg/pathway.html)*

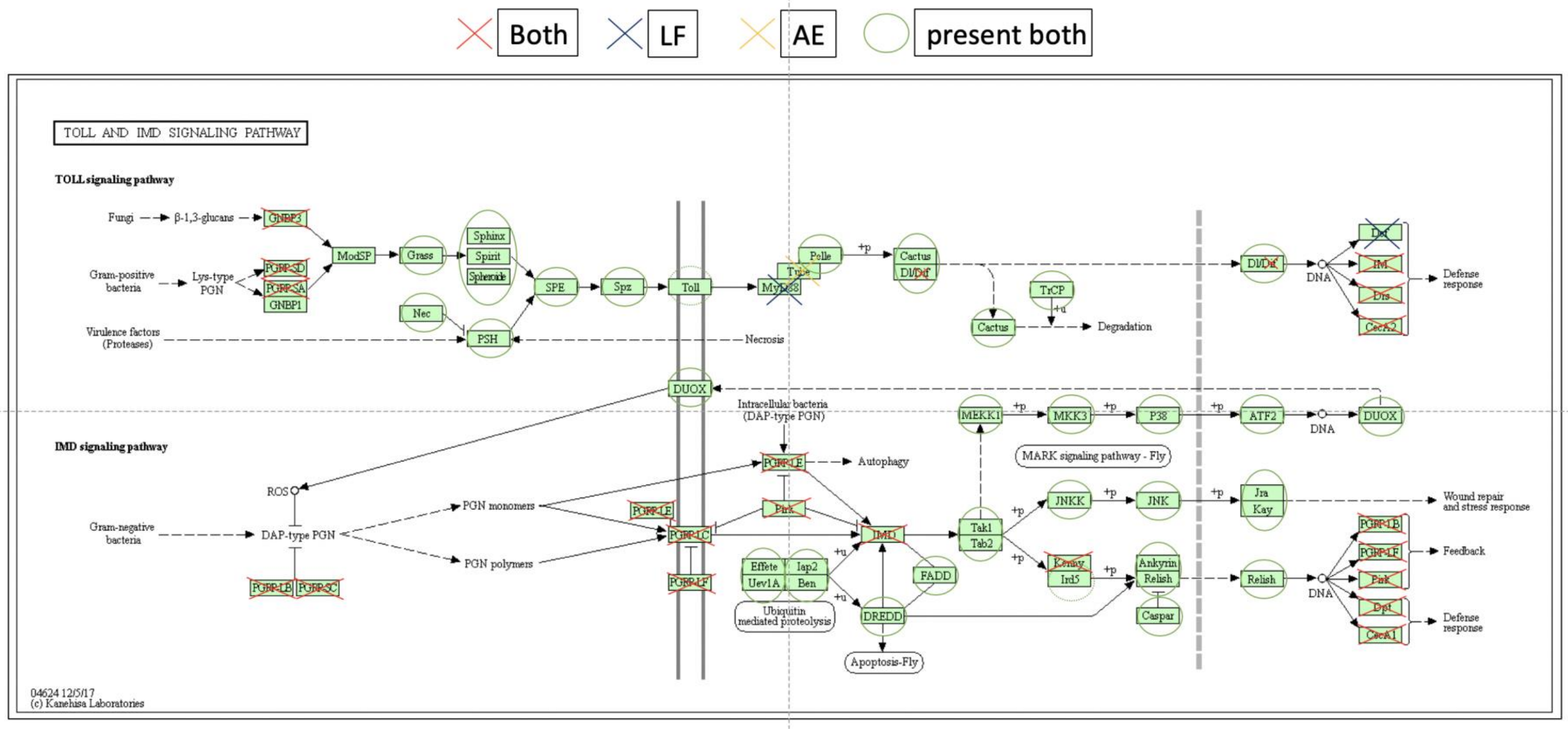

Osiris genes

The Osiris genes are an insect-specific gene family that underwent multiple tandem duplications early in insect evolution. All studied lineages of insects have approximately 20 Osiris genes, and most of these occur in a cluster, at least in well-assembled insect genomes (Shah, Dorer et al. 2012; Smith, Morandin et al. 2018). These genes are characterized by a domain of unknown function (DUF1676), a transmembrane domain, a signal peptide and a 3' AQXLAY motif (Shah, Dorer et al. 2012). These genes are essential for proper embryogenesis (Smoyer, Dorer et al. 2003) and for pupation (Schmitt-Engel, Schultheis et al. 2015; Andrade López, Lanno et al. 2017). The genes are also tied to immune and toxin-related responses (e.g. Andrade López, Lanno et al. 2017; Greenwood, Milutinovic et al. 2017), as well as to developmental polyphenism (Vilcinskas and Vogel 2016; Smith, Morandin et al. 2018). Developmental expression patterns are highly conserved for these genes, with major peaks during late embryogenesis and late pupal development (Smith, Morandin et al. 2018), and these genes are co-expressed with suites of genes associated with the regulation of chitin, leading to the hypothesis that these genes are associated with chitin remodeling via the endolytic pathway (Smith, Morandin et al. 2018). Expression levels for all Osiris genes are positively correlated, and correlations increase with both physical proximity and evolutionary distance. The three genes at the end of the cluster, in particular (Osi18-20) tend to be time-shifted in developmental expression relative to the other Osiris genes, suggesting that they are differently regulated (Smith, Morandin et al. 2018); this set of three genes maintains microsynteny in the cluster despite the many inversions and translocations seen within the Osiris cluster across insect genomes (Smith, Morandin et al. 2018).

We found 21 and 25 putative Osiris genes in each the *A. ervi* and *L. fabarum* genomes, respectively (Table 4). In *A. ervi,* these are spread across eight scaffolds, and in *L. fabarum*, seven scaffolds; the position and synteny of genes typically found in the main Osiris cluster are shown in Supplementary Figures 27 and 28. In insects with well assembled genomes, there is a consistent synteny of approximately 20 Osiris genes, and this cluster usually occurs in a ~150kbp stretch. These are, in order, Osi24, Osi1 – Osi20, though there have been some large inversions and translocations, such as in the beetle *T. castaneum* (Smith, Morandin et al. 2018). Gene synteny is conserved in the known Hymenoptera genomes. The middle of this conserved cluster (Osi7 – Osi15) is present as a syntenic group on scaffold237 in *A. ervi*, with Osi16-17 on scaffold397, and Osi18-20 on scaffold759. These scaffolds are relatively short and maybe physically linked. The situation is similar in *L. fabarum*, but the majority of the genes, Osi2-17, are together on scaffold tig00001007 (Supplementary Figure 28). Interestingly, the three genes at the end of the cluster, Osi18-20, appear to have undergone a duplication. These three genes are ancient paralogs (Smith, Morandin et al. 2018) and have a different timing of gene expression during development compared to other Osiris genes (Smith, Morandin et al. 2018). It is hypothesized that these three genes are co-regulated because of their highly correlated levels of gene expression and because their synteny seems to always be preserved despite rearrangements in other parts of the cluster. The Osiris cluster is largely devoid of non-Osiris genes in most of the Hymenoptera, but the assemblies of *A. ervi* and *L. fabarum* suggest that, if the cluster is actually syntenic in these species, there are interspersed non-Osiris genes (those are black boxes in Supplementary Figures 27 and 28).

In support of their role in defense, these genes were much more highly expressed in larvae than in adults of *L. fabarum* (Supplemental Table 14) – larvae live within the adult and we hypothesize that Osiris genes may be part of an adaptive response to dealing with hostile host environment. In both species, transcription in adults was very low, with fewer than 10 raw reads across all cDNA libraries sequenced, and often fewer than one read per library. Across the six sequenced transcriptomic libraries in *A. ervi*, just seven of the 22 putative Osiris genes had non-zero read-counts mapped to them; the highest of these had only 121 mapped reads (Supplemental data 11). In *L. fabarum*, the pattern was similar in adults: across the 59 sequenced adult cDNA libraries, 21 of the 26 predicted Osiris genes had non-zero read-counts, but only six of these had > 10 reads (Supplemental data 11). Patterns of expression were different in developing larvae of *L. fabarum*. Here, across 51 cDNA libraries sequenced from larvae all putative Osiris genes had non-zero read-counts, and many had thousands of reads mapped. Among these, 19 of the 26 annotated Osiris genes were significantly differentially expressed in larvae over adults, with fold changes up to 15x (Supplemental data 11).

Supplementary Table 14: Annotated Osiris genes (or outgroups) from A. ervi *as used in phylogenetic reconstruction. The Osi FastTree group refers to Fig. S20. Raw reads mapped came from six cDNA libraries (total reads approximately 1.83 x 10^8 pairs)*

A. ervi Protein	Osi (FastTree Group)	Raw reads mapped*
AE3004881	Out	4
AE3004882	OsiC	20
AE3015979	Osi15	0
AE3015980	Osi14	0
AE3015981	Osi11A	0
AE3015984	Osi10+	0
AE3015985	Osi10+	0
AE3015986	OsiE1/10	0
AE3015987	Osi9	0
AE3015989	Osi7	0
AE3017223	Osi16a	0
AE3017224	OsiL	0
AE3017226	Osi17	0
AE3017262	Osi1	10
AE3017642	Osi6	70
AE3017643	Osi5	0
AE3017820	Osi18	0
AE3017821	Osi19	0
AE3017822	Osi20	0
AE3018336	Osi2	121
AE3018337	Osi3	10
AE3019692	Osi24	41

Supplementary Table 15: Annotated Osiris genes (or outgroups) from L. fabarum *as used in phylogenetic reconstruction. The Osi FastTree group refers to Supplementary Figure 26. Raw reads mapped came from six cDNA libraries (total reads approximately 2.18 x 10^9 single end). Full results of expression analysis are in Supplemental Data 11.* ns*, no-significant difference.*

L. fabarum Protein	Osi (FastTree Group)	Raw reads mapped larvae	Raw reads mapped adults	Adjusted p-value in expression significance (adults vs larvae)
LF003979	Out	11932	545	2.6 x 10^{-29}
LF003980	OsiC	7921	81	1.2 x 10^{-49}
LF010203	Osi18	16	0	*ns*
LF010204	Osi19	25731	3	9.4 x 10^{-24}
LF010205	Osi20	11	0	*ns*
LF011148	Osi17	2872	5	3.2 x 10^{-22}
LF011149	OsiL	16	1	*ns*
LF011150	Osi16a	3590	9	2.1 x 10^{-17}
LF011151	Osi14	842134	7	5.8 x 10^{-98}
LF011152	Osi11A	9	0	*ns*
LF011153	Osi10+	2966	3	6.8 x 10^{-21}
LF011154	Osi11B	25	4	*ns*
LF011155	Osi10+	1090	1	4.2 x 10^{-8}
LF011156	Osi9	367397	44	8.7 x 10^{-102}
LF011157	Osi8	70487	361	2.1 x 10^{-39}
LF011158	Osi7	36599	1	7.0 x 10^{-25}
LF011159	Osi6	311721	45	2.5 x 10^{-95}
LF011160	Osi5	677	7	1.4 x 10^{-10}
LF011161	Osi3	12190	45	3.3 x 10^{-25}
LF011162	Osi2	5364	3	1.3 x 10^{-62}
LF011915	Osi23	38	6	*ns*
LF012925	Osi1	8	0	*ns*
LF013026	Osi20	16631	5	5.8 x 10^{-22}
LF013027	Osi19	11321	1	4.2 x 10^{-13}
LF013028	Osi18	8381	5	1.9 x 10^{-16}
LF015016	Osi17	1406	0	2.8 x 10^{-17}

Supplementary Figure 26: Approximate maximum likelihood phylogeny for holometabolan Osiris genes. All colored groupings have local support values > 0.9 except for Group A, Group C, and Osi22. Group names are from Smith et al. (in review) and Shah et al. (2012).

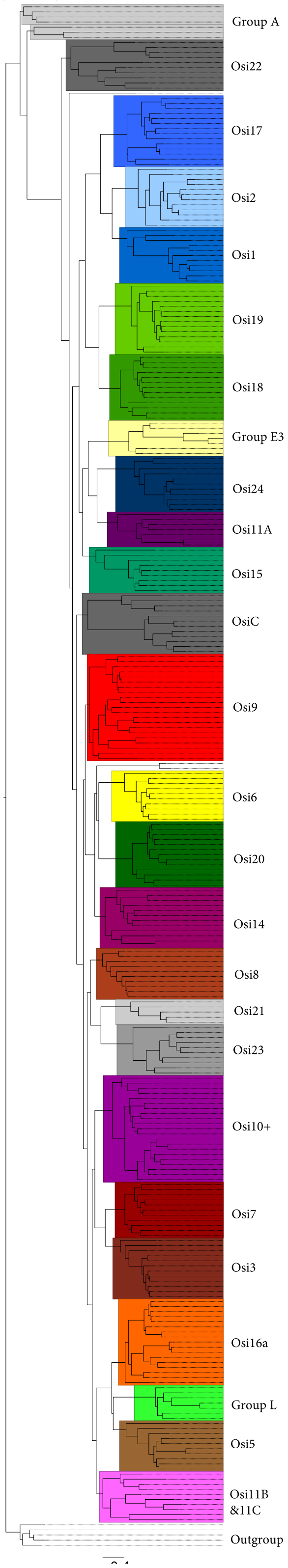

Supplementary Figure 27: Scaffolds from the A. ervi *genome assembly containing Osiris genes that are normally part of the conserved syntenic Osiris cluster. Most of these scaffolds are short and contain relatively few genes. The conserved synteny of this cluster in most insects suggests that these scaffolds may be contiguous. Color coding as in Supplementary Figure 26; non-black and numbered boxes are Osiris genes. Note difference in scaling of scaffolds.*

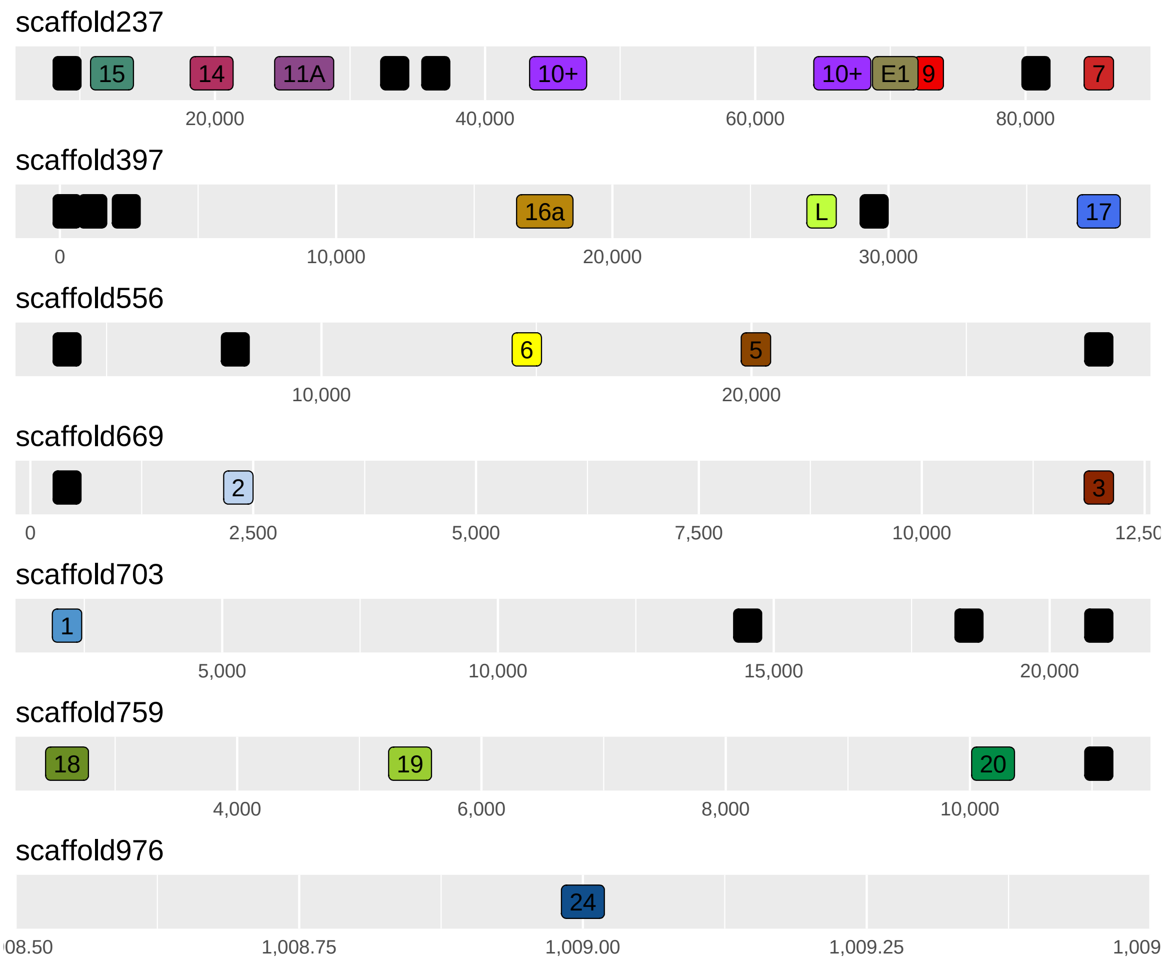

Supplementary Figure 28: Scaffolds from the L. fabarum *genome assembly, showing the Osiris genes that are normally part of the conserved syntenic Osiris cluster. As with* A. ervi*, the conserved synteny of these genes suggests that these relatively short scaffolds may be contiguous. Vertical separation of genes is to avoid overlap and has no biological meaning. Color coding is as in Supplementary Figure 26 and 27; non-black and numbered boxes are Osiris genes. Note differences in scaling of scaffolds.*

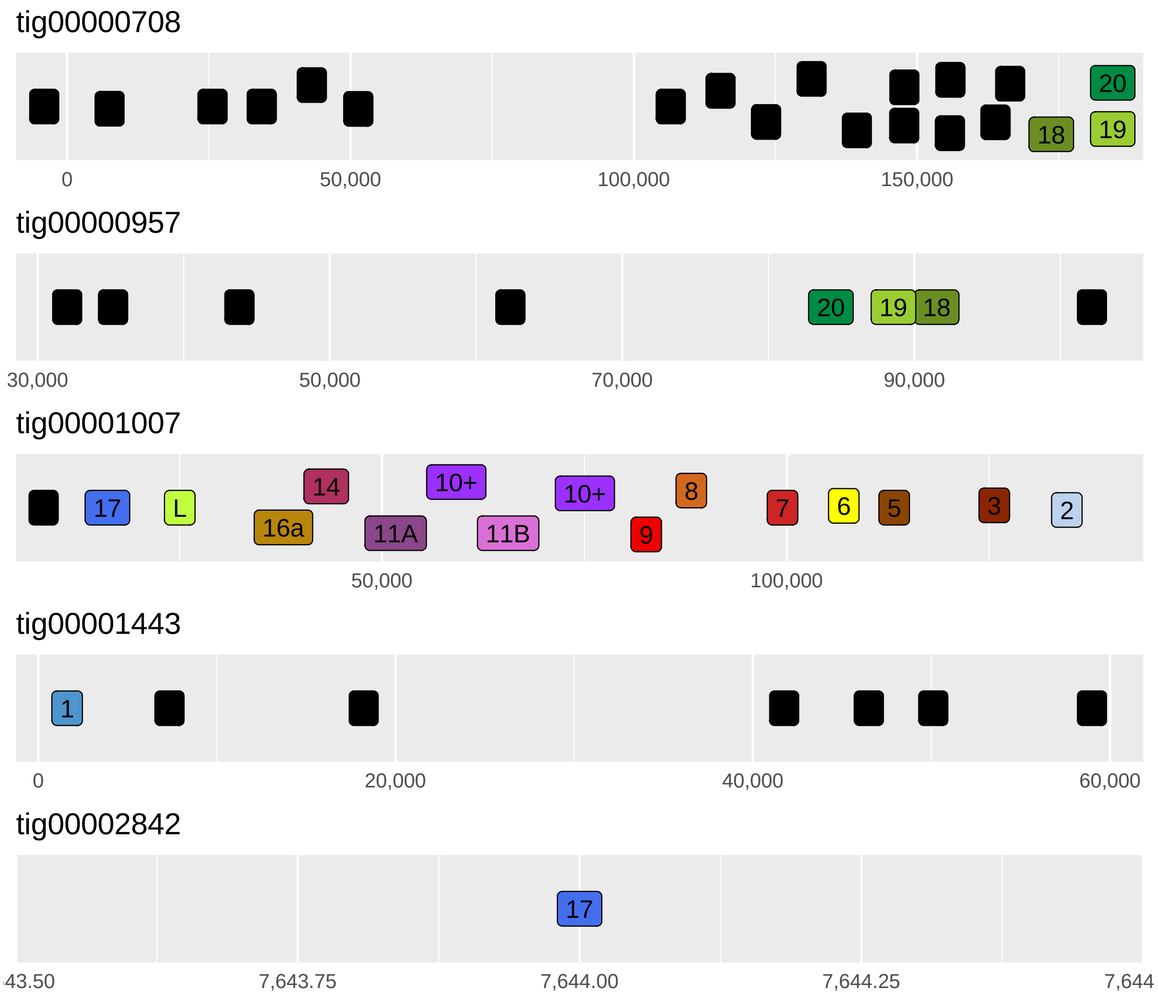

Oxidative phosphorylation (OXPHOS) genes

In most eukaryotes, mitochondria provide the majority of cellular energy (in the form of adenosine triphosphate, ATP) through the oxidative phosphorylation (OXPHOS) pathway. This pathway is composed of five protein complexes that utilize high energy electrons to produce a proton gradient across the inner mitochondrial membrane and the potential energy of this gradient is then used to phosphorylate ADP (adenosine diphosphate) into ATP. The OXPHOS pathway is conserved across eukaryotes and is unique because the complexes are comprised of both nuclear- and mitochondrial-encoded proteins. There are 13 protein-coding genes in nearly all eukaryotic mitochondrial genomes, all of which are used in the OXPHOS complexes, and there are ~67 "core" nuclear-encoded proteins that are used in these complexes (Porcelli, Barsanti et al. 2007). Unlike the mitochondrial-encoded genes, the nuclear-encoded genes vary in copy number across many organisms.

There were a total of 91 protein sequences of the nuclear-encoded oxidative phosphorylation genes from *Drosophila melanogaster* in the MitoDrome dataset (Barahimipour, Strenkert et al. 2015). Of these 91 total genes, 20 are *Drosophia* duplications of some of the remaining 71 "core" genes. We found 69 of these 71 core genes in the *A. ervi* genome, with four of them duplicated and one with three copies, making a total of 75 nuclear-encoded oxidative phosphorylation genes (Supplemental Table 16). In the *L. fabarum* genome, we found 69 of these 71 genes, with five of them duplicated, for a total of 74 nuclear-encoded oxidative phosphorylation genes (Supplemental Table 16). The gene sets of *A. ervi* and *L. fabarum* contained the same genes, and the same genes were duplicated in each, implying duplication events that occurred prior to the split from their most recent common ancestor. One of these duplicated genes appears to be duplicated again in *A. ervi*, or the other copy has gone missing in *L. fabarum*.

In addition to the duplications inferred above, additional duplications were found that are likely errors in the assembly. The *L. fabarum* genome also appears to have many scaffolds with portions of nearly identical sequence. The gene models in these regions encode identical proteins and these may represent sequence assembly errors. There were 16 models that showed this pattern, 14 had one extra identical copy and two had two extra identical copies. *A. ervi* also had two gene models with this same pattern with one extra identical copy. Future work should resolve these duplications in the assembly.

Supplementary Table 16: Summary of annotated OXPHOS genes in both genomes

	A. ervi	*L. fabarum*
Total genes (including duplicates)	75	74
Core OXPHOS genes	69	69
Missing "core genes"	2	2
Duplicated in *A. ervi* and *L. fabarum*	5	5
Unique duplicates	1	0
Models with identical copies (possible assembly errors)	2	16

Chemosensory Genes- Ionotropic receptors (IRs):

Supplementary Figure 29: Bootstrap tree built from predicted Ionotropic receptors (IRs) from A. ervi *and* L. fabarum *(this study),* Apis melifera, Nasonia vitripennis*, and Diachasma alloeum. Rooted using the well-conserved IR co-receptors (including Ir8a and IR25a). Branches with 90% bootstrap support are indicated by grey dots. Naming follows the convention described by Croset* et al. *(2010) for those with unclear orthology (e.g AerviIR101).*

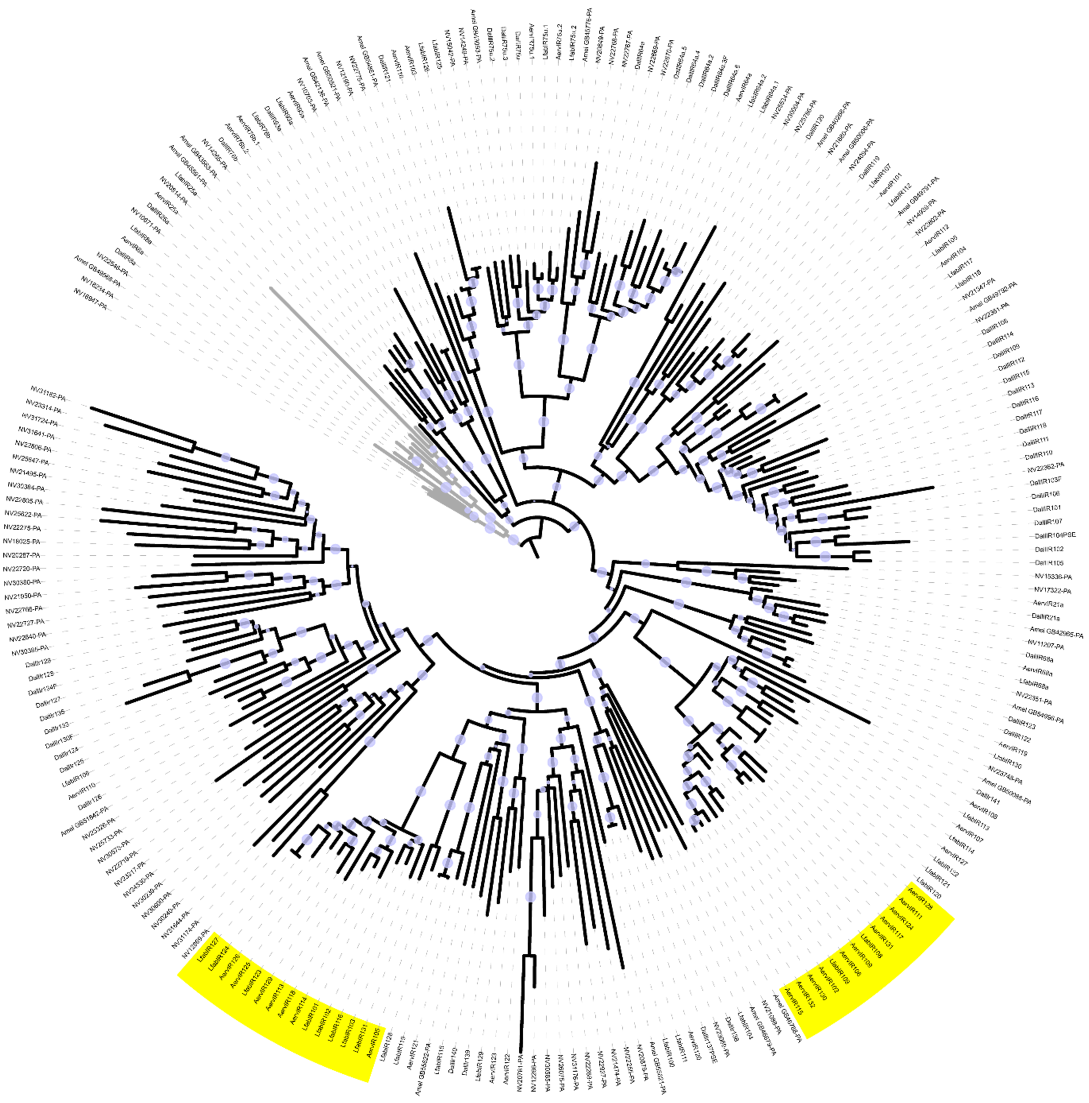

Sex Determination

Supplementary Table 17: Core sex determination genes

Core sex determination genes					
Species	*Gene*	*Scaffold*	*plus/minus*	*Region (approx)*	*E-value of hit with Asobara homolog*
A. ervi	transformer	scaffold17	plus	10000-15000	4.0×10^{-5}
A. ervi	transformerB	scaffold2824	minus	whole scaffold*	1.7
A. ervi	transformer-2	scaffold259	plus	33000-35000	3.0×10^{-27}
A. ervi	doublesex	scaffold43	minus	236000-225000	2.0×10^{-31}
L. fabarum	transformer	tig00000389	plus	105000-110000	0.008
L. fabarum	transformer-2	tig00001999	plus	60000-62000	4.0×10^{-27}
L. fabarum	doublesex	tig00000015	plus	14000-25000	2.0×10^{-30}

* Incomplete, only CAM-domain plus RS/P-rich regions, first (more conserved half) of gene not on scaffold

*Supplementary Table 18: Annotation of genes related to sex determination. *This second homolog of RBP1 on the same scaffold mirrors what is seen in the* Microplitis *and* Fopius *genomes.*

Genes related to sex determination					
Species	***Gene***	***Scaffold***	***plus/minus***	***Region (approximate)***	***e-value (to* Nasonia *homolog)***
A. ervi	*fruitless*	scaffold24	plus	589500-613000	1.0×10^{-91}
A. ervi	sex-lethal homolog	scaffold3	plus	151000-152500	4.0×10^{-63}
A. ervi	*CWC22 (aka nucampholin)*	scaffold18	minus	122000-119500	0
A. ervi	*CWC22* paralog	scaffold18	minus	421000-418000	0
A. ervi	*RBP1* homolog	scaffold39	minus	212500-213500	5.0×10^{-39}
A. ervi	*RBP1* homolog*	scaffold39	minus	204700-205300	3.00×10^{-31}
L. fabarum	*fruitless*	tig00000057	minus	321900-307000	4.00×10^{-88}
L. fabarum	sex-lethal homolog	tig00001640	minus	6500-5000	5.00×10^{-62}
L. fabarum	*CWC22 (aka nucampholin)*	tig00000545	plus	153000-155400	0
L. fabarum	*RBP1* homolog	tig00000173	minus	429000-429300	4.00×10^{-39}
L. fabarum	*RBP1* homolog*	tig00000173	minus	420000-420500	6.00×10^{-38}

Validation of DNA methylation genes

We confirmed these low levels of methylation in *A. ervi* by mapping this previously generated bisulfite sequencing data (Bewick, Vogel et al. 2017) to our genome assembly. Since this data originated from a strain that is genetically differentiated from that which we have sequenced, we allowed for up to 13 alignment mismatches per read between their sequences and ours (Supplementary Figure 30).

Mapping the whole genome bisulfite sequencing data to the reference genome while allowing for 13 alignment mismatches retained 625,765 reads (80.94% of all raw reads, 94.5 Mbp). Sequence coverage was low: 63,554 of all methylation-available cytosines in the reference genome were covered by at least one read, and only 1,216 sites by more than one read (see figure 2). Of all methylation-available cytosines, 63,409 sites were never methylated, 143 sites were always methylated, and two were variably methylated. These methylated sites were roughly equally distributed among methylation-available cytosine classes: CG, CHG and CHH sites were methylated at a rate of 0.154%, 0.179%, and 0.210%, respectively. Because very few sites were methylated >2 times, we did not have the power to estimate variation in per-site methylation.

Supplementary Table 19: Genes related to methylation. No homolog was detected for DNMT1 in either genome.

DNA methylation genes					
Species	***Gene***	***Scaffold***	***plus/minus***	***Region (approx)***	***e-value hit (to Nasonia homolog)***
A. ervi	*EEF1AKMT1* homolog	scaffold94	minus	144000-145500	1.00E-66
A. ervi	*DNMT3*	scaffold45	plus	581000-585100	5.00E-138
L. fabarum	*EEF1AKMT1* homolog	tig00000449	plus	13300-14100	5.00E-63
L. fabarum	*DNMT3*	tig00002022	plus	68000-70600	9.00E-117

Extended methods

Assemblies

We made whole-genome alignments between the *L. fabarum* and *A. ervi* genomes using NUCmer, which is part of the Mummer 2.0 software package. Alignments were made using the default settings (Kurtz, Phillippy et al. 2004), with the six *L. fabarum c*hromosomes and unincorporated scaffolds (1,407 pieces in all) used as the reference genome. To remove potentially erroneous matches and better view the alignment, we filtered to retain only the *A. ervi* scaffolds >1Mbp, and only including instances with three or more consecutive matches. These filtered matches were then visualized using the program Circos (Krzywinski, Schein et al. 2009).

Contamination filtering

We checked the assembled genomes for contamination using two approaches. First, we used blobtools (Laetsch and Blaxter 2017) to examine scaffolds based on their GC content and depth of sequencing coverage. Sequencing coverage was estimated in both species by mapping the cleaned paired-end Illumina reads back to the genome, using *bwa mem* (Li and Durbin 2009; Li 2013). Top BLAST matching was performed as suggested for blobtools, using BLASTn (Camacho, Coulouris et al. 2009), with an e-value cutoff of 1e-25, and retaining only the first match. All blobtools scaffolds that were not labelled as Arthropoda were manually examined via (a) the results from the blast-based screen of predicted genes (below) and (b) BLASTx searches of the predicted genes (default on NCBI webpage, March 2020). When there were no predicted genes, the entire scaffold was searched by BLASTx against the ncbi *nr* database.

Because these parasitoids develop within aphid, and because their host's genomes are also low in GC content, we also performed a search of the predicted genes to identify possible aphid contamination. For this, we used BLASTn to match predicted genes to either aphids or wasps. For the wasp, we used the other species sequenced here (i.e. *L. fabarum* for *A. ervi*, and vice versa). For the aphid, we used a combined reference of the *Acyrthosiphon pisum* (assembly v3, Genbank accession: GCA_005508785.1) and *Aphis glycines* genomes (assembly v6, biotype 1, Wenger, Cassone et al. 2017). This was implemented via a custom perl script that used megaBLAST (e-value 1x10-5) to search both references jointly and retain all matches. Sequences matching to more than one taxon were retained only if the bitscore gain between species was greater than 100. Reads without this clear bitscore difference were designated as "Multi-hit". We manually examined all cases where a scaffold had a gene matching to aphid. Scaffolds or predicted genes with no match were retained.

GC content

Nitrogen and carbon content

Carbon and nitrogen content were quantified separately for each gene in the CDS using the PROTPARAM online platform (Gasteiger, Hoogland et al. 2005), invoked via a perl script (Hussain 2016). These outputs were further manipulated in R (R-Core-Team 2012), and visualized by graphing the first two components of a Principal component analysis, or PCA (R packages factoextra, reshape, and ggplot2, Wickham 2007; Wickham 2009; Kassambara and Mundt 2016).

Differential expression analysis: larvae vs. adult *L. fabarum*

To examine the GC content of transcripts that are expressed at different life-history stages, we compared expression in adult and larval *L. fabarum*. This previously generated RNA-seq data was also utilized in the gene predictions for *L. fabarum*, and is available to view in the online genome server, hosted by bipaa (https://bipaa.genouest.org). This RNA-seq data (all 100-cycle, single end sequencing on an Illumina HiSeq2500) was generated from twelve lineages of parasitoid that had undergone experimental evolution on aphid hosts possessing different strains of *H. defensa* (as described in: Dennis, Patel et al. 2017). In both adults and larvae, the exact same lineages and conditions were sampled, collected in the course of an experimental evolution project at generations 11 (adult) and 14 (larvae). A total of 67 transcriptomic libraries were compared. Of these, 43 libraries came from pools of adults, sampled as 12-24h old virgin females (NCBI SRA PRJNA290156, Dennis, Patel et al. 2017). Larvae were part of a dual transcriptomic study, and were sampled 3-4 days after oviposition; we only used the 24 samples from the study that were deemed successful infections (NCBI SRA Accessions: SAMN10024115- SAMN10024165. Larval success described in: Dennis, Käch et al. in revision). In all cases, raw data was quality filtered and trimmed of potential Illumina primers as in the publications, using Trimmomatic (Bolger, Lohse et al. 2014), and was mapped to the *L. fabarum* draft genome using STAR in the "quantMode" (Dobin, Davis et al. 2013). Differentially expressed genes between adults and larvae were identified using DESeq2 (Love, Huber et al. 2014). To achieve this, we built a model in which samples were blocked by replicate population (4 per treatment), aphid host, and sampling environment (H. defensa free, or not, see: Dennis, Patel et al. 2017), taking into account the replicate cages from the experiment. The full model was ~ age + age:tmt + tmt+ host + cage. Within this, we used a pairwise contrast between the two ages (larvae vs adult) to identify genes with significantly higher expression at either stage (FDR < 0.05). The most highly expressed genes from these subsets were determined by ranking the assembled transcripts according to the total normalized counts for all libraries. We compared the GC content of all subsets of genes (differentially expressed or highly expressed) using a two-sided t-test, implemented in R.

Orphan gene ID

We identified orphan genes as those for which we could not find orthologs in any other sequenced parasitoid genomes. To do this, we generated clusters of orthologous and paralogous genes by comparing the predicted genes (CDS) from the genomes of *A. ervi* and *L. fabarum* to the predicted genes from parasitoids in both the *Braconidae* and *Ichneumonidae* (*Diachasma alloeum*, *Fopius arisanus*, *Macrocentrus cingulum*, *Microplitis demolitor* and *Nasonia vitripennis*). This was done using OrthoFinder (Emms and Kelly 2015) which is based on the analysis of pairwise sequence similarity scores obtained from an all-vs-all BLAST alignment among gene sequences between different species to identify orthogroups of genes. OrthoFinder produces a set of "Unassigned" genes that were not assigned to any orthogroup. We further examined these in two steps. First, we identified the genes that were present in the other genome assembled here using BLASTp (e-value < 0.001). Second, we identified species-specific genes, which we are calling orphan genes, by removing all genes that had hits to any other genes in the NCBI database. For this we identified matches at the protein level to the *nr* database, Swissprot database (using BLASTx), and the *nt* database (BLASTn), and at the DNA level against the *nt* database. In all cases, the e-value cut-off was 1e-10, and the databases were updated in June 2019. Within these putative orphans, we only retained those with transcriptomic support, based on the read counts generated for the GC analysis. We retained predicted genes of all lengths, as long as they had transcriptomic support, because manual inspection of some of these predicted genes suggested that they belonged to a region that actually contained a longer gene. The fasta file of the putative orphan genes for each species is available as Supplemental data 4 and 5.

Identification of *L. fabarum* venom proteins

For proteomic analysis, ten venom glands (see Supplemental Figure 17) were obtained by traction of the female ovipositor, isolated and dilacerated in 20 µl of Insect Ringer (KCl 182 mM; NaCl 46 mM; CaCl2 3 mM; Tris-HCl 10 mM) supplemented with a protease inhibitor cocktail (S8830; Sigma). The extract was centrifuged at 15,000 x g at 4°C for 10 min and the supernatant mixed with 4x Laemmli reducing buffer (Laemmli 1970) and loaded on a 12.5% SDS-PAGE (Supplemental Figure 18). The 16 most visible bands (numbered from the heavier to the lighter, Supplemental Figure 18) after silver staining (Morrissey 1981), were cut and sent for mass spectrometry. The 1D bands were treated with trypsin (Sequencing grade, Sigma) and alkylated reduced as previously described (Colinet, Deleury et al. 2013). Samples were analyzed by mass spectrometry using a hybrid Q-Orbitrap mass spectrometer (Q-Exactive, Thermo Fisher Scientific, United States) coupled to a nanoliquid chromatography (LC) Dionex RSLC Ultimate 3000 system (Thermo Fisher Scientific, United States). Samples were trapped with a C18 PepMap 300 trap column (300 µm × 5 mm, C18, 5 µm, 300 Å) and desalted with solvent A (water with 0.1% formic acid) for 3 min at a flow rate of 20 µL/min. Peptide separation was performed on an Acclaim PepMap RSLC capillary column (75 µm × 15 cm, nanoViper C18, 2 µm, 100 Å) at a flow rate of 300 nl/min. The analytical

gradient was run with various percentages of solvent B (acetonitrile with 0.1% formic acid) in the following manner: (1) 2.5–25% for 57 min, (2) 25–50% for 6 min, (3) 50-90% for 1 min, and (4) 90% for 10 min. Mass spectrometry (MS) spectra were acquired at a resolution of 35,000 within a mass range of 400–1,800 m/z. Ion accumulation was set at a maximum injection time of 100 ms. Fragmentation spectra of the 10 most abundant peaks (Top10 method) were acquired with high-energy collision dissociation (HCD) at a normalized collision energy of 27%.

All raw data files generated by MS were processed to generate mgf files and searched against (i) *L. fabarum* proteome predicted from genome (*Lysiphlebus fabarum* annotation v1.0 proteins) as well as (ii) *L. fabarum* transcriptome (Dennis, Patel et al. 2017; Dennis, Käch et al. in revision) using the MASCOT software (Perkins, Pappin et al. 1999). Search parameters were as follow: variable modifications: methionine oxidation and carbamidomethyl-cysteine, mass tolerance: 10 ppm on parent ion and 0.02 Da on fragment ions, and a maximum of two tryptic missed cleavages.

Venom sequence annotation and analysis

To identify similarities with known proteins, comparisons with NCBI non-redundant protein sequence database were performed using BLASTp with a cut-off e-value of 1e-7 and a cut-off identity of 30%. Signal peptide prediction was performed with SignalP (Emanuelsson, Brunak et al. 2007; Nielsen 2017). Identification of homologous venom proteins between *A. ervi* and *L. fabarum* was performed using BLASTp with a cut-off e-value of 1e-7 and a cut-off identity of 30%. Search for protein domains was performed using PfamScan (Finn, Bateman et al. 2013). Identification of venom protein genes was performed using BLAST tools in Apollo.

Identification of *A. ervi* and *L. fabarum* non-venomous γ-GT proteins was performed using *N. vitripennis* γ-GT sequences as queries in BLASTp and tBLASTn searches against *A. ervi* and *L. fabarum* proteomes predicted from the genomes (*Aphidius ervi* annotation v3.0 proteins and *Lysiphlebus fabarum* annotation v1.0 proteins) and *A. ervi* (Ballesteros, Gadau et al. 2017) and *L. fabarum* transcriptomes respectively. Multiple amino acid sequence alignments of GGT sequences were performed using MUSCLE (Edgar 2004). Phylogenetic analysis of GGT amino acid sequences was performed using maximum likelihood (ML) with PhyML 3.0 (Guindon and Gascuel 2003). SMS was used to select the best-fit model of amino acid substitution for ML phylogeny (Lefort, Longueville et al. 2017).

Community annotation

Desaturases

The desaturase genes of *A. ervi* and *L. fabarum* were analyzed in comparison with 13 other insects including seven Hymenoptera (*Apis mellifera*, *Bombus terrestris*, *Camponotus floridanus*, *Harpegnathos saltator*, *Linepithema humile*, *Nasonia vitripennis*, and *Pogonomyrmex barbatus*), one Coleopteran (*Tribolium castaneum*), two Diptera (*Drosophila melanogaster* and *Anopheles gambiae*), one Lepidopteran (*Bombyx mori*), and two hemimetabolous insects (*Acyrthosiphon pisum* and *Zootermopsis nevadensis*). All comparative species were previously analyzed in Helmkampf *et al.* (2015), except *Z. nevadensis*, which was analyzed recently as part of another study (Oeyen *et al*. in review). Gene annotations were carried out as follows: First, both the *A. ervi* and *L. fabarum* official genes sets and assembled genomes were searched for significant desaturase genes/regions using tBLASTn (Altschul, Madden et al. 1997) with an e-value cut-off of 0.001. For this purpose, desaturase gene peptide sequences of the jewel wasp, *Nasonia vitripennis*, representing nine acyl-CoA desaturase gene clades were used as queries to find homologous sequences in *A. ervi* and *L. fabarum*. These were: Desat A1, Desat A2, Desat B, Desat C, Desat D, Desat E, *ifc* (infertile crescent), *Cyt-b5-r* (Cytochrome b5-related), and *ERG3* (Ergosterol $\Delta^{5,6}$-Desaturase gene). The resulting OGS peptide sequences of *A. ervi* and *L. fabarum* were aligned to each other and the desaturase genes of *N. vitripennis* using MAFFT version 7 (Katoh and Standley 2013) to check for peptide sequence completeness (i.e., start and stop codons, exon number, and functional domains represented by three histidine motifs–HxxH, HxxxH, and HxxxH– that are characteristic of desaturase genes). Furthermore, all *A. ervi* and *L. fabarum* scaffold and contig regions containing significant matches to desaturase genes were extracted (+/- 5kb). In cases where no satisfactory gene model was available for a particular region, the extracted genome region (+/- 5kb) was analyzed with the gene prediction software, FGENESH+ (Solovyev 2007), using *N. vitripennis* as a basis for gene-finding parameters. The resulting additional peptide sequence predictions were aligned and checked as before, and efforts to identify missing regions were made by searching all available sequence data (including transcripts, unassembled scaffolds, or contigs) to determine the best possible gene model. Finally, where official gene set sequences were not available or were incorrectly predicted (i.e., prior to FGENESH+ analysis), manually annotated *A. ervi* and *L. fabarum* desaturase gene coding sequences were identified using the GeneWise genetic structure prediction software (Madeira, Park et al. 2019). After annotation, all putative desaturase genes were searched against NCBI's Conserved Domain Database (CDD; Marchler-Bauer, Bo et al. 2016) to identify protein domain presence and function. Details of all newly annotated desaturase genes examined in this study are compiled in Supplementary Table 12. The details of desaturase genes for 12 of the remaining 13 species are available in Helmkampf *et al*. (2015) and *Z. nevadensis* is available upon request.

Phylogenetic analysis of the *A. ervi* and *L. fabarum* First Desaturases (i.e., genes possessing a Delta9-FADS-like protein domain; Hashimoto, Yoshizawa et al. 2008) was performed to determine their relationships to the remaining six acyl-CoA desaturase gene subfamilies. Briefly, phylogenetic reconstruction was conducted by taking all First Desaturase gene peptide sequences greater than 250 AA (i.e., containing at least 2/3 of the average first desaturase gene) for *A. ervi*, *L. fabarum,* and 13 additional insect species (noted above and in Supplementary Figure 24) and aligning them using L-INS-i in MAFFT version 7 (Katoh and Standley 2013). This resulted in 164 sequences across 15 taxa. Next, poorly aligned positions and highly divergent regions were removed with Gblocks 0.91b, set to lowest stringency parameters to allow for larger blocks (Castresana 2000), which resulted in a final alignment containing 219 amino acid positions. The best fitting model of evolution (LG4X+I) was identified according to Akaike information criterion corrected for small sample size (AICc) with ModelTest-NG version 0.1.2 (Darriba, Posada et al. 2019). Using this model, a maximum likelihood tree was reconstructed with RAxML-NG version 0.6.0 (Kozlov, Darriba et al. 2019) and topology support values were obtained with 200 bootstrap replicates.

Osiris genes

Osiris gene orthologs in *A. ervi* and *L. fabarum* were determined with a two-part approach, candidate gene categorization followed by phylogenetic clustering. Candidate Osiris genes were generated using multiple, complementary methods, hidden Markov model searching, (HMMER3.1b2, Wheeler and Eddy 2013), and local alignment searching (BLAST, Altschul, Gish et al. 1990). A custom HMM was derived using all 24 well annotated and curated Osiris genes of *Drosophila melanogaster*. Next, an HMM search was performed on the *A. ervi* and *L. fabarum* proteomes, extracting all protein models with $P < 0.05$. Similarly, all *D. melanogaster* Osiris orthologs were searched in the annotated proteomes of *A. ervi* and *L. fabarum* using protein BLAST ($e < 0.05$). The top BLAST hit for each ortholog was then searched within each parasitoid genome for additional paralogs ($e < 0.001$). All unique candidates from the above approaches were then aligned using MAFFT (Katoh and Standley 2013), and an approximate maximum-likelihood phylogeny was constructed using FastTree (Price, Dehal et al. 2009) via the CIPRES science gateway of Xsede (Miller, Schwartz et al. 2015). For simplicity, only Osiris orthologs from selected Holometabola were used, mostly other species of Hymenoptera. The species used were: the fruit fly (*D. melanogaste*r), the tobacco hornworm moth (*Manduca sexta*), the silkworm moth (*Bombyx mori*), the flour beetle (*Tribolium castaneum*), the jewel wasp (*Nasonia vitripennis*), the honeybee (*Apis mellifera*), the buff tail bumble bee (*Bombus terrestris*), the red harvester ant (*Pogonomyrmex barbatus*), the Florida carpenter ant (*Camponotus floridanus*), and Jerdon's jumping ant (*Harpegnathos saltator*).

To examine expression in Osiris genes, we used the read-counts that were generated using STAR mapping against the whole genome, as part of the GC analysis (see above). To get an understanding of general level of expression, we compared raw reads that mapped to the putative Osiris genes in both species. To further explore differences between adults and larvae, we looked

at differential expression between the 59 previously generated adult RNA-seq libraries and 51 previously generated larval libraries (4-5 day old larvae). As with the read-mapping, this DE analysis was part of the same analysis used to compare larvae and adults for the GC analysis.

OXPHOS

Annotation of genes involved in the oxidative phosphorylation pathway (OXPHOS) was performed in several steps. Initial blasts were performed on the protein level, and matched predicted genes from the two genomes to a set of nuclear-encoded OXPHOS proteins from *Nasonia vitripennis* (Gibson, Niehuis et al. 2010; J. Gibson unpublished) and a similar set of proteins from *Drosophila melanogaster*, downloaded from the MitoComp website (www.mitocomp.uniba.it, Porcelli, Barsanti et al. 2007). Matches to *N. vitripennis* were taken preferentially and mismatches between the *N. vitripennis* and *Drosophila* matches were manually investigated. After this, we searched for OXPHOS genes that were not identified in the predicted proteins for *A. ervi* and *L. fabarum* by BLAST-searching the *N. vitripennis*/*Drosophila* protein against the entire genome sequences. This was used as evidence to build new gene models.

With all possible OXPHOS genes identified in the two genomes, gene models in *A. ervi* and *L. fabarum* were used to improve one another. This was done using available expression evidence (typically more for *A. ervi*). The protein models from both species were then aligned to one another and to *N. vitripennis* to find missing or extraneous sections. These were also compared to other hymenopteran proteins. Lastly, annotated proteins were BLAST-matched back to the *N. vitripennis* genome, to ensure they were reciprocal-best- BLAST hits.

Genes were named according to the existing *N. vitripennis* nomenclature (which has been extensively curated with NCBI to ensure the *Nasonia* naming was as consistent as possible, J. Gibson pers comm.) This is important to note, because the OXPHOS genes have many different names in different organisms (sometimes 5+ names). These were all added as synonyms.

To detect duplications and assembly errors, the gene models in the BLAST results to *N. vitripennis* were inspected beyond the top match, with additional inspection to ensure that it was an OXPHOS gene. True gene duplications were identified when there were two similar copies in one (or both) genome(s). These genes were given the same gene name, with A or B at the end, and the same gene symbol with A or B appended. Duplicates that were more similar to the *N. vitripennis* gene were given the A designation. There were many cases in the *L. fabarum* genome where two models had the exact same BLAST results to a given OXPHOS gene. Further investigation of the underlying genomic sequence showed that these were either extremely similar, or identical. These are likely assembly errors and differ by just a few SNPs. These were given identical gene names and gene symbols, are annotated as “Alleles” A and B, and are listed as “duplications” in the Supplemental Data 12. Further investigation should resolve true duplications from probable sequencing errors, and this suggests further refinement could benefit other gene models as well.

Validation of DNA methylation genes

To verify the low DNA methylation level of 0.5% reported by Bewick *et al*. (2017) and suggested by the absence of DNMT1, we mapped their low-coverage whole genome bisulfite sequencing data from *A. ervi* (NCBI Short Read Archive Accession GSE83497) against the *A. ervi* reference genome. This data set contains 773,136 unpaired short reads of 151bp length (116.7 Mbp in total). There was no information available on the wasp strain used in this study, however, most likely it was not the same as the strain from which the genome was sequenced. In other words, some genetic divergence should be expected between these two data sets.

Reads were mapped to the *A. erv*i genome with BS-Seeker2 (Guo, Fiziev et al. 2013), a full pipeline explicitly developed for mapping bisulfite sequencing data. Initially we ran BS-Seeker2 with default settings, which allows for a sequence mismatch of 4bp between reads and reference genome during mapping. The resulting 'mappability' (the percentage of all reads that map to a unique location on the genome) of the data was lower than what would be expected for data of the same species (62.79%).

In order to improve the mappability of the data, we repeated the analysis for a range of settings for allowed mapping mismatches (0 to 8 inclusive). Very low mappability is expected at zero mismatches since sequencing error introduces per read a few mismatches between read sequence and reference genome. A preliminary analysis of the output showed that allowing for up to 8 mismatches per read still improved the mappability considerably, and hence the parameter range of allowed mismatches was expanded up to 20 mismatches and the analyses repeated accordingly. At higher number of allowed mismatches the number of uniquely aligned reads asymptotically approached the total number of reads that have a single hit on the genome (Supplemental Figure 30).

Interestingly, the number of reads that have multiple hits on the genome does not change with varying numbers of allowed alignment mismatches (33,793 reads, i.e. 4.37%, Supplemental Figure 30). Note that reads having multiple matches are not informative on DNA methylation levels and hence these are automatically excluded from the analysis. Allowing for a higher number of mapping mismatches might introduce mapping hits on the reference genome that are false positives. Instead, we chose to aim for 95% inclusion of the reads that have a unique hit to the genome, and not to further increase this parameter for the number of allowed mismatches. This percentage was reached when allowing for 13 mismatches, and therefore this number was chosen for downstream analysis of DNA methylation levels.

Supplementary Figure 30: Summary of bisulfite sequencing against the A. ervi *genome. Mapping success was evaluated across a range of allowed mismatches, and 13 was chosen to obtain at least 95% inclusion of the data.*

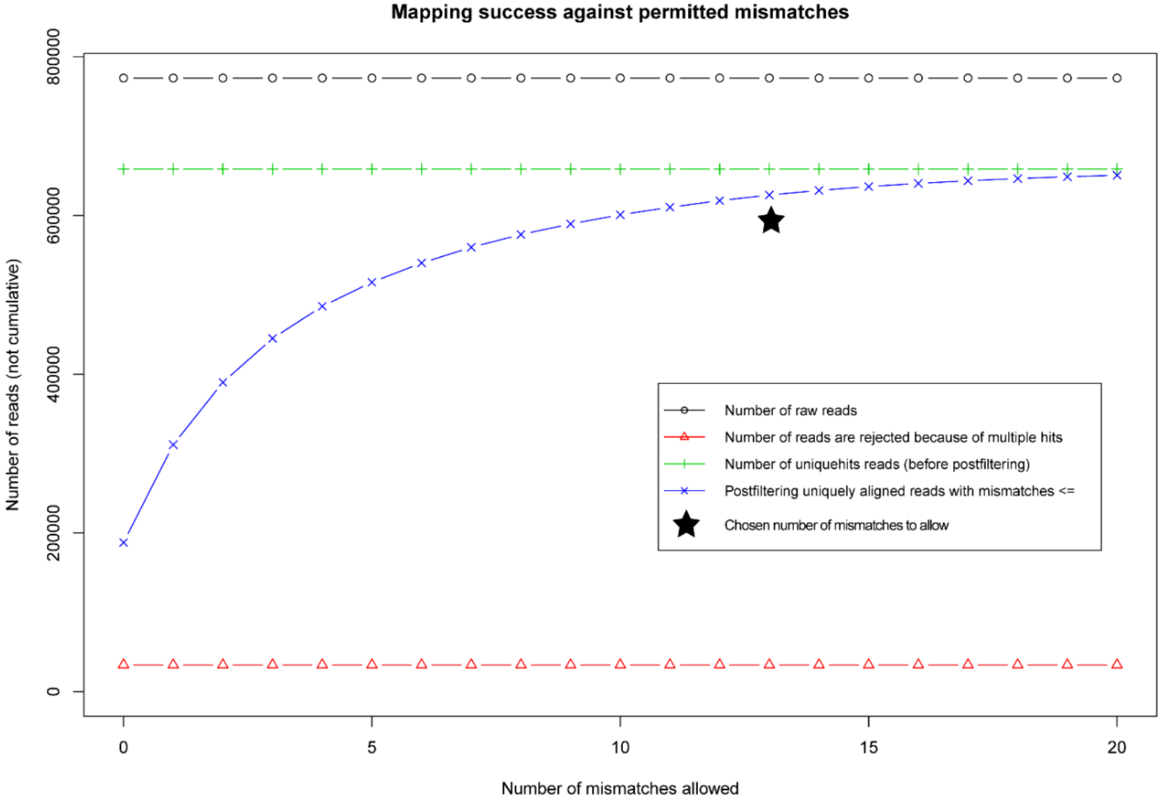

References

Acquisti, C., J. J. Elser, et al. (2009). "Ecological nitrogen limitation shapes the DNA composition of plant genomes." Molecular Biology and Evolution **26**(5): 953-956.

Altschul, S. F., W. Gish, et al. (1990). "Basic local alignment search tool." Journal of Molecular Biology **215**(3): 403-410.

Altschul, S. F., T. L. Madden, et al. (1997). "Gapped BLAST and PSI-BLAST: a new generation of protein database search programs." Nucleic Acids Research **25**(17): 3389-3402.

Andrade López, J. M., S. M. Lanno, et al. (2017). "Genetic basis of octanoic acid resistance in *Drosophila sechellia*: functional analysis of a fine-mapped region." Molecular Ecology **26**(4): 1148-1160.

Arp, A. P., W. B. Hunter, et al. (2016). "Annotation of the Asian Citrus Psyllid Genome Reveals a Reduced Innate Immune System." Frontiers in Physiology **7**: 570-570.

Ballesteros, G. I., J. Gadau, et al. (2017). "Expression differences in *Aphidius ervi* (Hymenoptera: Braconidae) females reared on different aphid host species." PeerJ **5**: e3640.

Barahimipour, R., D. Strenkert, et al. (2015). "Dissecting the contributions of GC content and codon usage to gene expression in the model alga *Chlamydomonas reinhardtii*." The Plant Journal **84**(4): 704-717.

Belshaw, R. and D. L. Quicke (2003). "The cytogenetics of thelytoky in a predominantly asexual parasitoid wasp with covert sex." Genome **46**(1): 170-173.

Bewick, A. J., K. J. Vogel, et al. (2017). "Evolution of DNA methylation across insects." Molecular Biology and Evolution **34**(3): 654-665.

Bolger, A. M., M. Lohse, et al. (2014). "Trimmomatic: a flexible trimmer for Illumina sequence data." Bioinformatics **30**(15): 2114-2120.

Bousquet, F., T. Nojima, et al. (2012). "Expression of a desaturase gene, *desat1*, in neural and nonneural tissues separately affects perception and emission of sex pheromones in *Drosophila*." Proceedings of the National Academy of Sciences **109**(1): 249.

Bragg, J. G. and A. Wagner (2009). "Protein material costs: single atoms can make an evolutionary difference." Trends in Genetics **25**(1): 5-8.

Buchon, N., N. Silverman, et al. (2014). "Immunity in *Drosophila melanogaster* — from microbial recognition to whole-organism physiology." Nature Reviews Immunology **14**: 796.

Camacho, C., G. Coulouris, et al. (2009). "BLAST+: architecture and applications." BMC Bioinformatics **10**: 421.

Cassone, B. J., J. A. Wenger, et al. (2015). "Whole genome sequence of the soybean aphid endosymbiont *Buchnera aphidicola* and genetic differentiation among biotype-specific strains." Journal of Genomics **3**: 85-94.

Castresana, J. (2000). "Selection of conserved blocks from multiple alignments for their use in phylogenetic analysis." Molecular Biology and Evolution **17**(4): 540-552.

Chaney, J. L. and P. L. Clark (2015). "Roles for synonymous codon usage in protein biogenesis." Annual Review of Biophysics **44**(1): 143-166.

Charroux, B. and J. Royet (2010). "*Drosophila* immune response: From systemic antimicrobial peptide production in fat body cells to local defense in the intestinal tract." Fly **4**(1): 40-47.

Chertemps, T., L. Duportets, et al. (2006). "A female-specific desaturase gene responsible for diene hydrocarbon biosynthesis and courtship behaviour in *Drosophila melanogaster*." Insect Molecular Biology **15**(4): 465-473.

Colinet, D., C. Anselme, et al. (2014). "Identification of the main venom protein components of *Aphidius ervi*, a parasitoid wasp of the aphid model *Acyrthosiphon pisum*." BMC Genomics **15**(1): 342.

Colinet, D., E. Deleury, et al. (2013). "Extensive inter- and intraspecific venom variation in closely related parasites targeting the same host: the case of *Leptopilina* parasitoids of *Drosophila*." Insect Biochemistry and Molecular Biology **43**(7): 601-611.

Croset, V., R. Rytz, et al. (2010). "Ancient protostome origin of chemosensory ionotropic glutamate receptors and the evolution of insect taste and olfaction." PLoS Genetics **6**(8): e1001064.

Dallerac, R., C. Labeur, et al. (2000). "A Δ9 desaturase gene with a different substrate specificity is responsible for the cuticular diene hydrocarbon polymorphism in *Drosophila melanogaster*." Proceedings of the National Academy of Sciences **97**(17): 9449.

Darriba, D., D. Posada, et al. (2019). "ModelTest-NG: A new and scalable tool for the selection of DNA and protein evolutionary models." Molecular Biology and Evolution **37**(1): 291-294.

Degnan, P. H., Y. Yu, et al. (2009). "*Hamiltonella defensa*, genome evolution of protective bacterial endosymbiont from pathogenic ancestors." Proceedings of the National Academy of Sciences **106**(22): 9063-9068.

Dennis, A. B., H. Käch, et al. (in revision). "Dual RNA-seq in an aphid parasitoid reveals plastic and evolved adaptation." BioRxiv.

Dennis, A. B., V. Patel, et al. (2017). "Parasitoid gene expression changes after adaptation to symbiont-protected hosts." Evolution **71**(11): 2599-2617.

Dobin, A., C. A. Davis, et al. (2013). "STAR: ultrafast universal RNA-seq aligner." Bioinformatics **29**.

Drosophila 12 Genomes, C., A. G. Clark, et al. (2007). "Evolution of genes and genomes on the *Drosophila* phylogeny." Nature **450**: 203.

Edgar, R. C. (2004). "MUSCLE: multiple sequence alignment with high accuracy and high throughput." Nucleic Acids Research **32**(5): 1792-1797.

Elser, J. J., W. F. Fagan, et al. (2006). "Signatures of Ecological Resource Availability in the Animal and Plant Proteomes." Molecular Biology and Evolution **23**(10): 1946-1951.

Elsik, C. G., K. C. Worley, et al. (2014). "Finding the missing honey bee genes: lessons learned from a genome upgrade." BMC Genomics **15**(1): 86.

Emanuelsson, O., S. Brunak, et al. (2007). "Locating proteins in the cell using TargetP, SignalP and related tools." Nature Protocols **2**(4): 953-971.

Emms, D. M. and S. Kelly (2015). "OrthoFinder: solving fundamental biases in whole genome comparisons dramatically improves orthogroup inference accuracy." Genome Biology **16**(1): 157.

Evans, J. D., K. Aronstein, et al. (2006). "Immune pathways and defence mechanisms in honey bees Apis mellifera." Insect Molecular Biology **15**(5): 645-656.

Fang, S., A. Takahashi, et al. (2002). "A mutation in the promoter of desaturase 2 is correlated with sexual isolation between *Drosophila* behavioral races." Genetics **162**(2): 781-784.

Finn, R. D., A. Bateman, et al. (2013). "Pfam: the protein families database." Nucleic Acids Research **42**(D1): D222-D230.

Gan, H. M., S. M. Linton, et al. (2019). "Two reads to rule them all: Nanopore long read-guided assembly of the iconic Christmas Island red crab, *Gecarcoidea natalis* (Pocock, 1888), mitochondrial genome and the challenges of AT-rich mitogenomes." Marine Genomics **45**: 64-71.

Gasteiger, E., C. Hoogland, et al. (2005). Protein identification and analysis tools on the ExPASy server. The Proteomics Protocols Handbook. J. M. Walker. Totowa, NJ, Humana Press**:** 571-607.

Gerardo, N. M., B. Altincicek, et al. (2010). "Immunity and other defenses in pea aphids, *Acyrthosiphon pisum*." Genome Biology **11**(2): R21.

Gibson, J. D., O. Niehuis, et al. (2010). "Contrasting patterns of selective constraints in nuclear-encoded genes of the oxidative phosphorylation pathway in holometabolous insects and their possible role in hybrid breakdown in *Nasonia*." Heredity **104**: 310.

Greenwood, J. M., B. Milutinovic, et al. (2017). "Oral immune priming with *Bacillus thuringiensis* induces a shift in the gene expression of *Tribolium castaneum* larvae." BMC Genomics **18**(1): 329.
Guindon, S. and O. Gascuel (2003). "A simple, fast, and accurate algorithm to estimate large phylogenies by maximum likelihood." Systematic Biology **52**(5): 696-704.
Guo, W., P. Fiziev, et al. (2013). "BS-Seeker2: a versatile aligning pipeline for bisulfite sequencing data." BMC Genomics **14**: 774-774.
Hashimoto, K., A. C. Yoshizawa, et al. (2008). "The repertoire of desaturases and elongases reveals fatty acid variations in 56 eukaryotic genomes." Journal of Lipid Research **49**(1): 183-191.
Hazel, J. R. and E. E. Williams (1990). "The role of alterations in membrane lipid composition in enabling physiological adaptation of organisms to their physical environment." Progress in Lipid Research **29**(3): 167-227.
Helmkampf, M., E. Cash, et al. (2015). "Evolution of the insect desaturase gene family with an emphasis on social Hymenoptera." Molecular Biology and Evolution **32**(2): 456-471.
https://bipaa.genouest.org. "BioInformatics Platform for Agroecosystem Arthropods (BIPAA)." from https://bipaa.genouest.org.
Hussain, M. K. (2016). Automate-the-protparam-web-server.
Jiang, Z., D. H. Jones, et al. (2013). "Comparative analysis of genome sequences from four strains of the *Buchnera aphidicola* Mp endosymbiont of the green peach aphid, Myzus persicae." BMC Genomics **14**: 917-917.
Kanehisa, M. and S. Goto (2000). "KEGG: kyoto encyclopedia of genes and genomes." Nucleic Acids Res **28**.
Kassambara, A. and F. Mundt (2016). Factoextra: extract and visualize the results of multivariate data analyses. r. package.
Katoh, K. and D. M. Standley (2013). "MAFFT Multiple Sequence Alignment Software Version 7: Improvements in performance and usability." Molecular Biology and Evolution **30**(4): 772-780.
Kozlov, A. M., D. Darriba, et al. (2019). "RAxML-NG: a fast, scalable and user-friendly tool for maximum likelihood phylogenetic inference." Bioinformatics **35**(21): 4453-4455.
Krzywinski, M., J. Schein, et al. (2009). "Circos: An information aesthetic for comparative genomics." Genome Research **19**(9): 1639-1645.
Kurtz, S., A. Phillippy, et al. (2004). "Versatile and open software for comparing large genomes." Genome Biology **5**(2): R12.
Labeur, C., R. Dallerac, et al. (2002). "Involvement of Desat1 gene in the control of *Drosophila melanogaster* pheromone biosynthesis." Genetica **114**(3): 269-274.
Laemmli, U. K. (1970). "Cleavage of structural proteins during the assembly of the head of bacteriophage T4." Nature **227**: 680.
Laetsch, D. R. and M. L. Blaxter (2017). "BlobTools: Interrogation of genome assemblies [version 1; peer review: 2 approved with reservations]. ." F1000Research **6**(1287).
Lefort, V., J.-E. Longueville, et al. (2017). "SMS: Smart Model Selection in PhyML." Molecular Biology and Evolution **34**(9): 2422-2424.
Legeai, F., S. Shigenobu, et al. (2010). "AphidBase: a centralized bioinformatic resource for annotation of the pea aphid genome." Insect Molecular Biology **19 Suppl 2**(0 2): 5-12.
Lemaitre, B. and J. Hoffman (2007). "The host defence of *Drosophila melanogaster*." Ann Rev Immunol **25**.
Li, H. (2013). "Aligning sequence reads, clone sequences and assembly contigs with BWA-MEM." arXiv preprint: 1303.3997.
Li, H. and R. Durbin (2009). "Fast and accurate short read alignment with Burrows-Wheeler transform." Bioinformatics **25**.

Liepert, C. and K. Dettner (1993). "Recognition of aphid parasitoids by honeydew-collecting ants: The role of cuticular lipids in a chemical mimicry system." Journal of Chemical Ecology **19**(10): 2143-2153.

Liepert, C. and K. Dettner (1996). "Role of cuticular hydrocarbons of aphid parasitoids in their relationship to aphid-attending ants." Journal of Chemical Ecology **22**(4): 695-707.

Los, D. A. and N. Murata (1998). "Structure and expression of fatty acid desaturases." Biochimica et Biophysica Acta (BBA) - Lipids and Lipid Metabolism **1394**(1): 3-15.

Love, M., W. Huber, et al. (2014). "Moderated estimation of fold change and dispersion for RNA-seq data with DESeq2." Genome Biology **15**(12): 550.

Madeira, F., Y. m. Park, et al. (2019). "The EMBL-EBI search and sequence analysis tools APIs in 2019." Nucleic Acids Research **47**(W1): W636-W641.

Mapleson, D., G. Garcia Accinelli, et al. (2016). "KAT: a K-mer analysis toolkit to quality control NGS datasets and genome assemblies." Bioinformatics **33**(4): 574-576.

Marchler-Bauer, A., Y. Bo, et al. (2016). "CDD/SPARCLE: functional classification of proteins via subfamily domain architectures." Nucleic Acids Research **45**(D1): D200-D203.

Miller, M. A., T. Schwartz, et al. (2015). "A RESTful API for access to phylogenetic tools via the CIPRES science gateway." Evolutionary bioinformatics online **11**: 43-48.

Miyazaki, M. and J. M. Ntambi (2003). "Role of stearoyl-coenzyme A desaturase in lipid metabolism." Prostaglandins, Leukotrienes and Essential Fatty Acids **68**(2): 113-121.

Monticelli, L. S., L. T. H. Nguyen, et al. (2019). "The preference-performance relationship as a means of classifying parasitoids according to their specialization degree." Evolutionary Applications **0**(ja).

Morin-Poulard, I., A. Vincent, et al. (2013). "The *Drosophila* JAK-STAT pathway in blood cell formation and immunity." JAK-STAT **2**(3): e25700.

Morrissey, J. H. (1981). "Silver stain for proteins in polyacrylamide gels: a modified procedure with enhanced uniform sensitivity." Anal Biochem **117**(2): 307-310.

Moto, K. i., M. G. Suzuki, et al. (2004). "Involvement of a bifunctional fatty-acyl desaturase in the biosynthesis of the silkmoth, *Bombyx mori*, sex pheromone." Proceedings of the National Academy of Sciences of the United States of America **101**(23): 8631.

Myllymäki, H., S. Valanne, et al. (2014). "The *Drosophila* imd signaling pathway." The Journal of Immunology **192**(8): 3455.

NCBI, N. C. f. B. I., Bethesda (MD). "NCBI nr database, available from ftp.ncbi.nlm.nih.gov/blast/db/."

Nielsen, H. (2017). Predicting Secretory Proteins with SignalP, available at: http://www.cbs.dtu.dk/services/SignalP/. Protein Function Prediction: Methods and Protocols. D. Kihara. New York, NY, Springer New York**:** 59-73.

Perkins, D. N., D. J. C. Pappin, et al. (1999). "Probability-based protein identification by searching sequence databases using mass spectrometry data." Electrophoresis **20**(18): 3551-3567.

Porcelli, D., P. Barsanti, et al. (2007). "The nuclear OXPHOS genes in insecta: a common evolutionary origin, a common cis-regulatory motif, a common destiny for gene duplicates." BMC Evol Biol **7**: 215.

Price, M. N., P. S. Dehal, et al. (2009). "FastTree: Computing large minimum evolution trees with profiles instead of a distance matrix." Molecular Biology and Evolution **26**(7): 1641-1650.

Pyne, N. J. and S. Pyne (2017). "Sphingosine 1-Phosphate receptor 1 signaling in mammalian cells." Molecules (Basel, Switzerland) **22**(3): 344.

R-Core-Team (2012). R: A language and environment for statistical computing. R Foundation for Statistical Computing, Vienna, Austria., R Development Core Team.

Rago, A., D. G. Gilbert, et al. (2016). "OGS2: genome re-annotation of the jewel wasp *Nasonia vitripennis*." BMC Genomics **17**(1): 678.

Rhoads, A. and K. F. Au (2015). "PacBio Sequencing and its applications." Genomics, proteomics & bioinformatics **13**(5): 278-289.
Roelofs, W. L. and A. P. Rooney (2003). "Molecular genetics and evolution of pheromone biosynthesis in Lepidoptera." Proceedings of the National Academy of Sciences of the United States of America **100**(Suppl 2): 14599-14599.
Schmitt-Engel, C., D. Schultheis, et al. (2015). "The iBeetle large-scale RNAi screen reveals gene functions for insect development and physiology." Nature Communications **6**: 7822.
Shah, N., D. R. Dorer, et al. (2012). "Evolution of a Large, Conserved, and Syntenic Gene Family in Insects." G3: Genes|Genomes|Genetics **2**(2): 313.
Shigenobu, S., H. Watanabe, et al. (2000). "Genome sequence of the endocellular bacterial symbiont of aphids *Buchnera sp.* APS." Nature **407**: 81.
Smith, C. R., C. Morandin, et al. (2018). "Conserved roles of Osiris genes in insect development, polymorphism and protection." Journal of Evolutionary Biology **31**(4): 516-529.
Smoyer, L. K., D. R. Dorer, et al. (2003). "Phenotype of the Triplo-lethal locus of *Drosophila melanogaster* and its suppression by hyperoxia." Genet Res **82**(3): 163-170.
Thorpe, P., C. M. Escudero-Martinez, et al. (2018). "Shared transcriptional control and disparate gain and loss of aphid parasitism genes." Genome Biology and Evolution **10**(10): 2716-2733.
Valanne, S., J.-H. Wang, et al. (2011). "The *Drosophila* toll signaling pathway." The Journal of Immunology **186**(2): 649.
Vilcinskas, A. and H. Vogel (2016). "Seasonal phenotype-specific transcriptional reprogramming during metamorphosis in the European map butterfly *Araschnia levana*." Ecol Evol **6**(11): 3476-3485.
Wenger, J. A., B. J. Cassone, et al. (2017). "Whole genome sequence of the soybean aphid, *Aphis glycines*." Insect Biochemistry and Molecular Biology.
Wenger, J. A., B. J. Cassone, et al. (2017). "Whole genome sequence of the soybean aphid, Aphis glycines." Insect Biochemistry and Molecular Biology: 102917.
Wheeler, T. J. and S. R. Eddy (2013). "nhmmer: DNA homology search with profile HMMs." Bioinformatics **29**(19): 2487-2489.
Wickham, H. (2007). "Reshaping data with the reshape package." Journal of Statistical Software; Vol 1, Issue 12 (2007).
Wickham, H. (2009). Ggplot2 elegant graphics for data analysis. New York, Springer.
Xue, B., A. P. Rooney, et al. (2007). "Novel sex pheromone desaturases in the genomes of corn borers generated through gene duplication and retroposon fusion." Proceedings of the National Academy of Sciences **104**(11): 4467.
Yin, C., M. Li, et al. (2018). "The genomic features of parasitism, polyembryony and immune evasion in the endoparasitic wasp *Macrocentrus cingulum*." BMC Genomics **19**: 420.
